# Genome shuffling enables quantitative trait locus mapping in *Bacillus subtilis*

**DOI:** 10.1038/s41467-026-72929-0

**Published:** 2026-08-05

**Authors:** Delyana P. Vasileva, Hari B. Chhetri, Leah H. Hochanadel, Jared C. Streich, John H. Lagergren, Matthew J. Lane, Apurv Mhatre, Oumar Sacko, Marco N. Allemann, Edanur Oksuz, Dana L. Carper, Benjamin Rubino, Sadie Chitwood, Zachary D. Schmitz, Dawn M. Klingeman, Paul E. Abraham, Richard J. Giannone, Daniel A. Jacobson, Joshua K. Michener

**Affiliations:** 1 Biosciences Division, Oak Ridge National Laboratory, Oak Ridge, TN USA; 2 Center for Bioenergy Innovation, Oak Ridge National Laboratory, Oak Ridge, TN USA; 3 Bredesen Center for Interdisciplinary Research and Graduate Education, University of Tennessee, Knoxville, TN USA

**Keywords:** Bacterial genetics, Bacteriology, Genetic association study

## Abstract

Genetic mapping is a powerful tool for eukaryotic genetics that has only been applied to bacteria in limited circumstances. Quantitative trait locus (QTL) mapping generally relies on sexual recombination to break linkages between genes, yet bacteria rarely undergo sufficient homologous recombination to generate suitable mapping populations. In this work, we used iterative biparental genome shuffling by protoplast fusion in *Bacillus subtilis* to generate a population of bacteria with substantial random recombination throughout their genomes. Individual shuffled progeny were arrayed in well plates, resequenced, and characterized for a range of complex phenotypes, including spore germination and swarming motility. Genetic mapping of the resulting phenotypes identified high-confidence QTLs of moderate size (~15 kb), and these associations were validated through targeted genetic swaps. This *B. subtilis* QTL population can easily be used to map additional phenotypes and the general approach for QTL mapping is applicable to a wide range of Gram-positive and Gram-negative bacteria using diverse methods for genome-wide recombination.

## Abstract (web summary)

Genetic mapping methods that link phenotypes with causal genetic variants have been difficult to apply in bacteria since they require intraspecific recombination. Here, Vasileva et al. construct a panel of recombinant bacteria that enables genetic mapping of diverse phenotypes.

## Introduction

Advances in next-generation DNA sequencing technologies have provided an unprecedented view of the vast genetic landscape of bacteria. However, linking DNA sequences to observable physical traits remains challenging. Even in the best-studied model bacteria, many genes have unknown functions^1^, and little is known about the genetic networks underlying complex phenotypes or the functional effects of natural sequence variation in bacterial genes and regulatory elements^2^. Arrayed and pooled genetic screens, including genome-wide deletion libraries, transposon insertion sequencing, clustered regularly interspaced short palindromic repeats (CRISPR) methods and expression libraries, have revolutionized bacterial functional genomics and identified genetic determinants underlying multiple phenotypes^3–8^. However, these approaches are limited to inferring gene function based on loss-of-function or gain-of-function experiments and fail to account for more subtle genetic variation or to capture the complex genetic architecture of quantitative polygenic traits.

Genome-wide association studies (GWAS) and QTL mapping are highly effective techniques in eukaryotic genetics that link genetic variation to phenotypic traits^9,10^. GWAS typically uses genetically diverse natural populations, whereas QTL mapping relies on experimentally generated recombinant populations derived from controlled crosses between defined parents. Most commonly applied in macroscopic sexually recombining eukaryotes, such as humans and plants, these methods have provided substantial insight into the genetic basis of complex phenotypes and uncovered multiple novel biological mechanisms^11,12^. Similar approaches have been developed in yeast to enable fine-scale genetic mapping and analysis of epistatic interactions^13,14^. Unfortunately, the use of quantitative genetic mapping techniques in bacteria has been limited by the lack of native systems for sexual recombination and the generally low recombination rates within bacterial populations^15^. While the GWAS approach has been successfully applied to extant bacterial populations in several instances, these studies have predominantly focused on pathogenic species with large collections of clinical isolates that enabled the assembly of suitably diverse mapping populations^16–19^. Even then, the mapping approach was most successful when applied to genetic variants that arise under strong selection pressure in recombination hotspots, including variants linked to antibiotic resistance^17^. Since isolating non-pathogens in sufficient numbers is difficult and most bacteria do not undergo sufficient recombination for GWAS, QTL mapping would be more broadly applicable. Genetic mapping with experimentally generated inter-strain recombinants has been attempted in some specific cases, where chromosome regions can be transferred between strains through conjugal mechanisms, including distributive conjugal transfer in mycobacteria^20^ and mass allelic exchange (MAE) in *Escherichia coli*^21^. However, a comprehensive experimental framework for QTL mapping is lacking in bacteria.

**Fig. 1:**
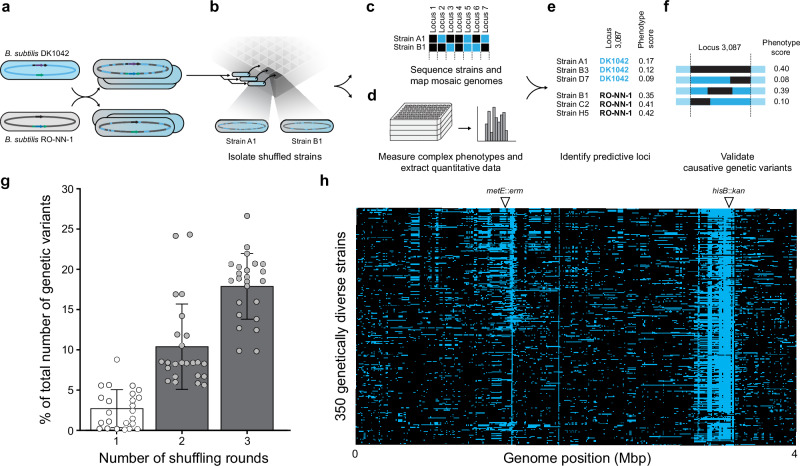
Overview of bacterial QTL mapping. **a** Replacing amino acid biosynthesis genes with antibiotic resistance markers in genetically divergent parents allows for selection of recombinant hybrid strains containing different marker allele combinations following genome shuffling by protoplast fusion (Supplementary Fig. 1). **b** Repeated rounds of shuffling increase genetic diversity, generating mapping populations of highly recombinant progeny. **c** The progeny are sequenced to identify genetic contributions from each parent and **d** phenotyped. **e** Statistical mapping methods are then used to identify genetic loci that correlate with a phenotype of interest. **f** These correlations are experimentally validated through CRISPR-mediated markerless genetic swaps. **g** The fractions of recombined genome after one round of shuffling between *B. subtilis* RO-NN-1 and 168^22^ (white bar) and after two and three rounds of shuffling between strains RO-NN-1 and DK1042 (gray bars) are shown. The number of genetic variants was determined through whole-genome sequencing. *n* = 24 recombinant strains from each shuffle. Data represent mean ± standard deviation (SD). **h** A fine-scale recombination map of the *B. subtilis* QTL population. Each row represents a sequenced individual strain from the 350-strain mapping panel, with blue bars indicating recombined fragments originating from strain DK1042 and black regions representing the genomic portions inherited from strain RO-NN-1. Arrows in (**h**) indicate locations of the selection markers Δ*metE*::*erm* and Δ*hisB*::*kan*. Source data are provided as a Source data file.

We have previously shown that genome shuffling by protoplast fusion between genetically diverse *Bacillus* strains generates frequent, unbiased, genome-wide recombination that mimics the effects of sexual recombination^22^. In this study, we leveraged protoplast fusion to establish a bacterial QTL mapping platform (Fig. 1a–f). We demonstrated the utility of this approach by identifying causal genetic loci with high statistical power, as well as its potential to reveal the genetic basis of a broad range of complex phenotypes.

## Results and discussion

### Development of a highly recombinant bacterial QTL mapping panel

Our previous work showed that a single round of genome shuffling between pairs of *Bacillus* strains generates recombinant strains with genomes primarily derived from one of the parents but containing multiple unselected DNA fragments originating from the second parent that are randomly recombined throughout the chromosome^22^. For example, when *B. subtilis* strains 168 and RO-NN-1 were shuffled, DNA fragments from *B. subtilis* 168 replaced, on average, 2.7% of the genome of *B. subtilis* RO-NN-1 (Fig. 1g) and the sizes of the recombined regions were broadly distributed, including short inserts (<100 bp) and large multigene fragments (>60 kb)^22^. These two strains vary phenotypically^23^ and differ in their core genomes at 68,538 single-nucleotide polymorphisms (SNPs) (~98% nucleotide identity), averaging ~1 SNP every 50 bp. In addition, 189 genes are unique to strain RO-NN-1, and 496 genes are unique to the chromosome of strain DK1042. We therefore reasoned that a high frequency of recombination between the chromosomes of strains with this level of genetic divergence would generate sufficient admixture of both core genes and accessory pathways to potentially drive mappable variation in phenotypes of interest.

To construct a mapping population, we selected *B. subtilis* RO-NN-1 as the first parent due to its success at genome shuffling in our previous work, and DK1042 as the second parent, a genetically competent strain that exhibits phenotypes lost in the domesticated strain 168^24^. The statistical confidence to identify causal loci is determined by the degree of recombination and the resulting decrease in linkage between genetic variants in the mapping population. To reduce linkage between genetic loci, we aimed to maximize recombination through repeated cycles of genome shuffling using a backcrossing approach (Supplementary Fig. 1). We replaced amino acid biosynthesis genes (*hisB* and *metE*), located roughly in the opposite regions of the chromosome, with antibiotic resistance markers in RO-NN-1 and DK1042, and then crossed the resulting auxotrophic strains with wild-type DK1042 and RO-NN-1, respectively. In each cross, we selected for recombinant strains containing all possible combinations of markers from both parents, generating S1 progeny. A pool of S1 strains was then backcrossed to strain DK1042, creating S2 progeny and the same process was repeated once more to generate S3 progeny. Recursive protoplast fusion efficiently increased genome-wide recombination with strains from the S3 progeny inheriting, on average, ~18.0% of genomic fragments from strain DK1042, creating extensive genetic diversity across their chromosomes (Fig. 1g). Successive rounds of shuffling and selection introduced long recombination fragments originating from strain DK1042 flanking the selected markers. To introgress these regions, we generated a set of S4 recombinant strains by backcrossing a pool of S3 strains to strain RO-NN-1 (Supplementary Figs. 1 and 2). We determined the genome architecture of 573 individual S3 strains and 50 S4 strains. After removing nearly identical strains, we retained a total of 350 genetically diverse strains with 59,571 SNPs as a mapping population (Fig. 1h, Supplementary Figs. 3 and 4). Due to our crossing and selection scheme (Supplementary Fig. 1), most shuffled strains in the QTL population had recombination around the *hisB* locus, and some had recombination near the *metE* locus (Fig. 1h). All strains, however, showed extensive random genome-wide unselected recombination. Future efforts may aim to achieve more balanced distribution of selected recombination events across the genome by diversifying selection strategies.

### Bacterial QTL mapping identifies causal genetic variants in coding regions and regulatory elements

**Fig. 2:**
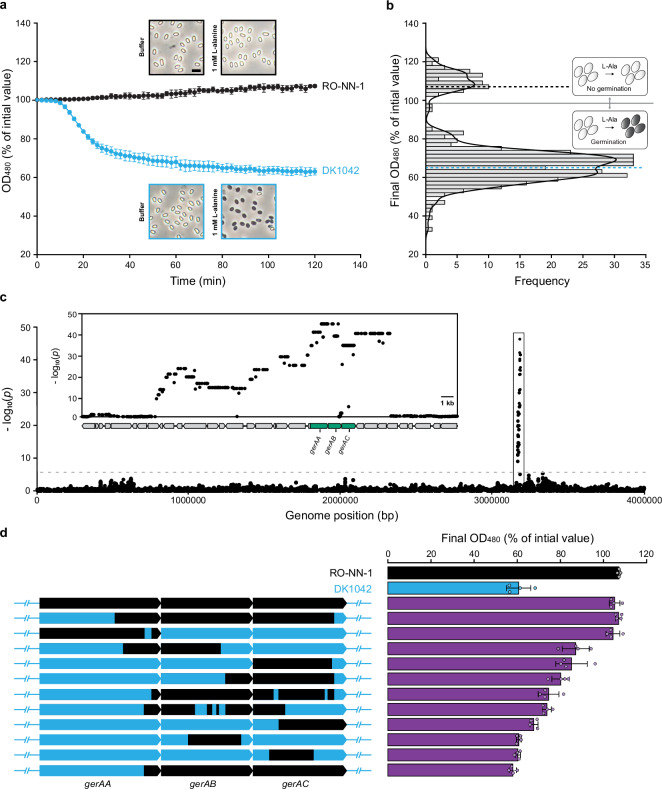
Bacterial QTL mapping identifies genetic variants associated with spore germination. **a** *B. subtilis* DK1042 and RO-NN-1 exhibit different spore germination abilities with L-alanine. Spores of the two strains were incubated with 1 mM L-alanine, and the decrease in optical density was monitored as phase-bright spores transitioned to dark-phase germinated spores. *n* = 4 independent experiments. Data are represented as mean ± SD. Similar results were obtained with 50 mM L-alanine (Supplementary Fig. 5b). Representative phase-contrast images of DK1042 and RO-NN-1 spores after 120 min incubation with buffer and 1 mM L-alanine are shown. Scale bar, 2 µm. **b** Distribution of spore germination phenotypes across the QTL mapping population. A histogram of the percent reduction in optical density after 120 min of incubation of spores from each strain with 1 mM L-alanine is shown (Supplementary Fig. 6 and Supplementary Data 1). Black and blue dashed lines indicate the spore germination data from RO-NN-1 and DK1042, respectively. The solid grey line indicates the threshold we defined for spore germination. **c** Manhattan plot summarizing the statistical significance of associations between each genetic variant along the genome and spore germination across the *B. subtilis* QTL mapping population. The inset shows a zoomed-in view of the region containing the spore germination QTL. Candidate causal genetic loci from the *gerA* operon are highlighted in green. Association testing was performed using a mixed linear model. Bonferroni correction was applied to account for multiple comparisons. The gray dashed line represents the Bonferroni-corrected significance threshold (*p* ≤ 0.05). **d** Genetic swaps validate causal genetic loci. Gene segments within the *gerA* operon in strain DK1042 were replaced with corresponding segments from strain RO-NN-1 using a CRISPR-based approach, and their spore germination abilities were evaluated. The bar plot shows optical densities measured after incubation of spores of each strain with 1 mM L-alanine for 120 min. Data from the full library of gene-swapped strains is shown in Supplementary Fig. 12. Black regions indicate genetic fragments originating from strain RO-NN-1, and blue regions indicate segments from strain DK1042. *n* = 4 independent experiments. Data are represented as mean ± SD. Source data are provided as a Source data file.

We next tested whether the mapping population could be used to identify the causal alleles responsible for complex phenotypes. Spore germination is an important developmental phenotype with direct implications for the food industry and human disease^25^. Previous studies have identified variation in spore germination between *B. subtilis* RO-NN-1 and 168^23^, prompting us to use this trait as an initial test case for QTL mapping. To analyze the heritability of spore germination, we generated spores from the two QTL parents and the full panel of shuffled strains and then measured their response to L-alanine as the sole germinant. As previously described, we observed significant differences in the spore germination abilities of the two parents (Fig. 2a and Supplementary Fig. 5). Spores of strain DK1042 germinated rapidly, whereas spores of strain RO-NN-1 failed to germinate when incubated with L-alanine for 120 min. The recombinant strains exhibited a high degree of phenotypic variability with a bimodal distribution (Fig. 2b, Supplementary Fig. 6, and Supplementary Data 1). We therefore performed QTL mapping using spore germination as a binary phenotype. To estimate intrinsic noise arising from genetic variation across the QTL population, we first conducted separate permutation analyses using true genotypes with randomized binary spore germination phenotypes, none of which showed any significant association (Supplementary Fig. 7). QTL mapping of the spore germination data identified a 18.5 kb high-confidence locus of strong effect and the most significant genetic variants were located within a three-gene operon encoding the germinant receptor GerA (Fig. 2c and Supplementary Fig. 8)^26,27^. The *gerA* operons of the two parental strains contained 166 sequence variants, corresponding to 46 amino acid differences (Supplementary Figs. 9 and 10). Analysis of the *gerA* region in the recombinant progeny showed that DK1042 variants were more prevalent in shuffled strains that germinate with L-alanine, whereas RO-NN-1 variants were more common in those that did not germinate, demonstrating the significance of the genotype-trait association (Supplementary Fig. 11).

To validate our QTL inference, we performed allele replacement experiments by swapping regions within the DK1042 *gerA* operon with corresponding genetic fragments from strain RO-NN-1 and evaluated spore germination of the resulting strains in response to L-alanine (Fig. 2d, Supplementary Figs. 12 and 13). Spores derived from a strain where the entire DK1042 *gerA* operon was replaced with the corresponding RO-NN-1 allele failed to germinate under these conditions, in a manner similar to RO-NN-1. Strains harboring different *gerA* regions derived from RO-NN-1 showed diverse quantitative phenotypes, including germination rates similar to the QTL parents as well as intermediate phenotypes. Specifically, genetic variants located in the genes encoding the GerAA and GerAC subunits of the germination receptor, as well as interactions between these variants, were associated with different spore germination rates (Supplementary Fig. 12). In contrast to the response with L-alanine, spores of strain RO-NN-1 germinated in response to L-valine in a GerA-dependent manner (Supplementary Figs. 14 and 15), indicating that both strains produce active GerA but with differing sensitivity to common germinants.

By identifying genetic variants within functional genes that affect complex phenotypes, we demonstrated that QTL mapping provides a framework to characterize the complex biological effects of natural genetic variation in bacteria beyond simple loss-of-function mutations. Targeted genetic swaps can be applied to refine the range of candidate genetic variants in a QTL region and distinguish the effects of SNPs within causal genes. Although the majority of the shuffled strains in our QTL population contained either DK1042 or RO-NN-1 variants in the *gerA* locus, seven strains harbored single variants originating from strain DK1042 or different combinations of variants from the two parents (Supplementary Fig. 11). With a larger mapping population, the density of recombination events achieved with our cross design has the potential to facilitate fine-scale mapping without the need for additional experimentation to resolve composite QTLs. While the spore germination phenotype produced a bimodal signal at the genome scale, we detected the effects of local genetic interactions arising from coevolution within the *gerA* operon, suggesting that our QTL mapping approach has the potential to reveal epistasis in complex phenotypes.

**Fig. 3:**
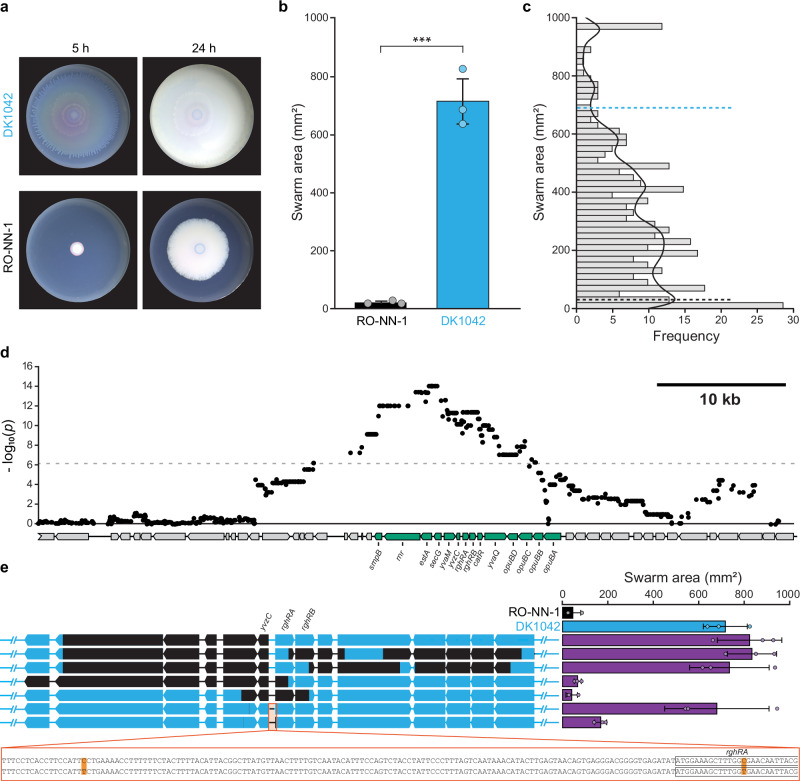
Regulatory genetic variants drive phenotypic differences in swarming motility. **a** Comparison of the swarming motility of *B. subtilis* DK1042 and RO-NN-1 over the course of 5 h and 24 h. Representative data from three biological experiments is shown. **b** Areas covered by the DK1042 and RO-NN-1 swarms measured after 5 h of incubation. *n* = 3 independent experiments. Data are represented as mean ± SD and were analyzed using a two-tailed Student’s *t* test. ***p = 0.00024. **c** Distribution of swarming motility phenotypes across the QTL population. A histogram of swarm areas after incubation for 5 h is shown (Supplementary Data 2). **d** Inset of a Manhattan plot (Supplementary Fig. 18) shows a 50-kb genomic region, including the QTL for swarming motility. Candidate causal genetic loci are highlighted in green. The gray dashed line represents the Bonferroni-corrected significance threshold (*p* ≤ 0.05). **e** Genetic swaps within the QTL validate the association and identify two candidate causal genetic variants. The DNA sequence corresponding to the genomic region containing the two candidate causal genetic variants is highlighted in orange. Black regions indicate genetic fragments originating from strain RO-NN-1, and blue regions indicate segments from strain DK1042. *n* = 3 independent experiments. Data are represented as mean ± SD. Source data are provided as a Source data file.

We next tested the performance of our QTL platform using an additional phenotype, swarming motility—a model multicellular trait that varies substantially among different *Bacillus* isolates (Supplementary Fig. 16)^28^. Our analysis revealed significant differences in the swarming motility of the two QTL parents, with DK1042 exhibiting a robust swarming phenotype, whereas RO-NN-1 was relatively immotile (Fig. 3a, b). The QTL population displayed a broad continuous distribution of swarming motility (Fig. 3c and Supplementary Data 2). Consistent with the spore germination test case, permutation analyses using randomized continuous swarming motility phenotypes produced no statistically significant signal (Supplementary Fig. 17), and mapping of the trait identified a highly significant QTL of moderate size (14.9 kb) (Fig. 3d and Supplementary Fig. 18). We then systematically screened loci within the QTL by introducing genetic regions from RO-NN-1 into strain DK1042 using our allele replacement approach (Fig. 3e and Supplementary Fig. 19). These experiments narrowed the candidate causal variants down to two SNPs: one located in the regulatory region of the transcriptional regulator *rghR*, and the other a synonymous mutation within the coding sequence of *rghR*. A modified DK1042 strain containing the two candidate causal SNPs showed reduced swarming motility, similar to that of the RO-NN-1 strain (Fig. 3e). RghR is known to repress the expression of *rapG* and *rapH*, which encode inhibitors of the surfactin biosynthetic cluster *srfA*^29^. Proteomic and metabolomic analyses showed that both wild-type RO-NN-1 and the DK1042 strain containing the two candidate causal SNPs exhibited significantly lower expression of RghR and SrfA, as well as reduced surfactin production, compared to strain DK1042 (Supplementary Fig. 20a, b, and Supplementary Data 3). Furthermore, the addition of surfactin to the growth medium enabled swarming motility in strain RO-NN-1 (Supplementary Fig. 20c, d), suggesting that differences in surfactin levels underlie the phenotypic variation. These results highlight that our QTL mapping approach can uncover subtle regulatory mechanisms and genetic factors contributing to phenotypes related to public goods production, which would likely be overlooked in traditional pooled genetic screens.

### Genome shuffling creates mappable variation across a wide range of bacterial traits

**Fig. 4:**
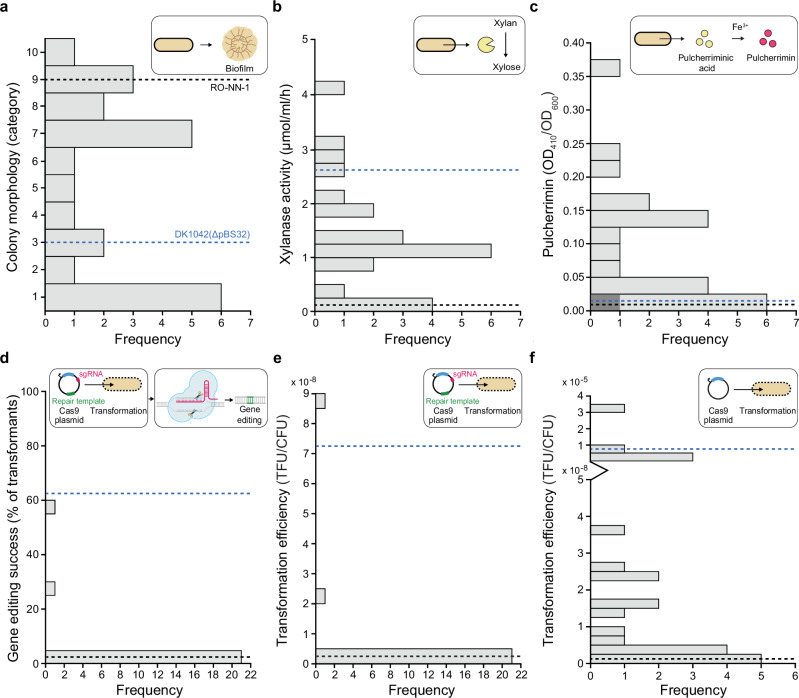
Bacterial QTL mapping is applicable to a wide range of complex phenotypes. Phenotypic variability was tested across the 23 most genetically diverse strains from the QTL population (Supplementary Fig. 3 and Supplementary Data 4) for a variety of traits. **a** Distribution of colony morphology phenotypes under biofilm-promoting conditions. A histogram summarizing colony morphology category frequencies (Supplementary Fig. 21) is shown. **b** Distribution of extracellular xylanase activity phenotypes. A histogram of xylanase activities (Supplementary Fig. 22) is shown. **c** Distribution of pulcherrimin biosynthesis phenotypes. A histogram of pulcherrimin production (Supplementary Fig. 23) is shown. All strains except RO-NN-1 and one shuffled strain indicated by a dark gray bar carried the pulcherrimin biosynthesis cluster. **d** Distribution of CRISPR-Cas9 gene editing efficiency phenotypes. A histogram of gene editing efficiencies (Supplementary Fig. 26a) is shown. **e**, **f** Distributions of transformation efficiencies using either a CRISPR-Cas9 plasmid carrying sgRNA targeting *trpC* and a repair template (Supplementary Fig. 26b), or an empty CRISPR-Cas9 plasmid (Supplementary Fig. 26c). Black and dark blue dashed lines indicate phenotypic data from RO-NN-1 and the cured DK1042 strain JMB300, respectively. CFU colony-forming units, TFU transforming units. Source data are provided as a Source data file.

For a phenotype to be mapped, it must vary across the mapping population. To explore further the breadth of phenotypes that can be mapped, we selected the 23 most genetically diverse shuffled strains from the QTL population (Supplementary Fig. 3 and Supplementary Data 4) and evaluated phenotypic variability across this panel (Fig. 4). We reasoned that any phenotype which varied in this subset of strains could, if desired, be mapped in the larger population. We tested both phenotypes relevant to microbial ecology and traits of interest for bacterial engineering. Since none of the shuffled strains in the QTL population carried the DK1042-derived plasmid pBS32 and the effects of this plasmid on some of the tested phenotypes is not known, we used a cured strain as the parental control in these experiments.

Biofilm formation is a complex phenotype that involves multiple genes and genetic interactions^30^. Colony biofilms grown on agar surfaces are a widely used experimental model for studying biofilm formation. We determined colony biofilm architectures across the 23-strain panel and the parental controls and categorized the colonies based on their morphology (Fig. 4a and Supplementary Fig. 21). Our analysis revealed that genome shuffling generated extensive variability in colony patterns. In addition, we evaluated enzyme activity and secondary metabolite production phenotypes, including extracellular xylanase activity (Fig. 4b and Supplementary Fig. 22) and biosynthesis of pulcherrimin, an iron-binding pigment (Fig. 4c and Supplementary Fig. 23). Finally, we assessed phenotypes relevant to genetic engineering, including promoter leakiness (Supplementary Fig. 24), transformation efficiency (Supplementary Fig. 25) and CRISPR-Cas9 gene editing efficiency (Fig. 4d and Supplementary Fig. 26a). These assays revealed substantial phenotypic variability among the 23 shuffled strains, with individual strains showing distinct phenotypes across the different assays (Supplementary Fig. 27). We frequently identified shuffled strains exhibiting phenotypes that are not intermediate of the parental strains but rather represent quantitatively or qualitatively new phenotypes. These findings indicated that the tested traits are complex and that the progeny represent a new mixture of alleles, enabling the discovery and mapping of novel phenotypes. Furthermore, our QTL population could be used to map composite phenotypes, such as CRISPR-Cas9 editing efficiency, and to dissect the relationship between their underlying component phenotypes, such as transformation efficiency and Cas9 toxicity (Fig. 4e, f, and Supplementary Fig. 26b, c).

While our mapping population used only two parents, multiparental crosses have been applied to improve the power and resolution of QTL studies in eukaryotic genetics by introducing a broader spectrum of genetic and phenotypic variation^31^. To test whether a similar multiparental approach could be applied in bacterial QTL mapping populations, we shuffled three genetically divergent *Bacillus* strains, *B. subtilis* RO-NN-1, *B. subtilis* DK1042, and *B. spizizenii* TU-B-10 (Supplementary Fig. 28). The resulting progeny showed diverse inheritance patterns with multiple recombination events from all three parents, demonstrating that a more complex mapping population could be constructed if desired.

### The bacterial QTL mapping approach can be extended to a wide range of bacteria

**Fig. 5:**
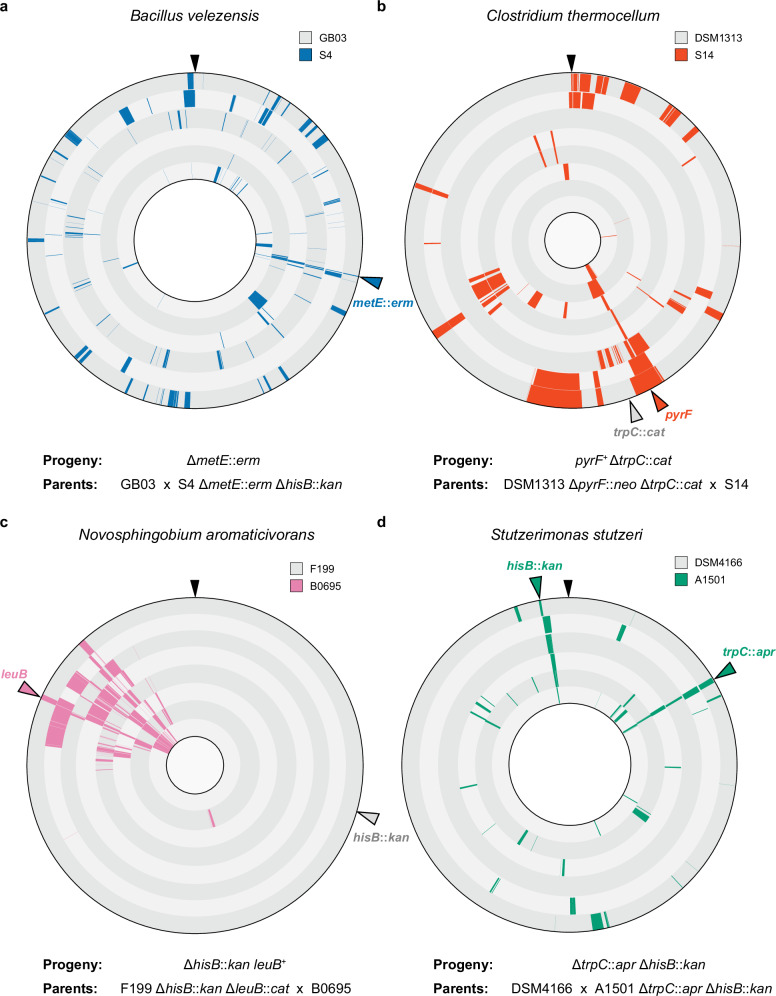
Genome-wide mappable genetic variation can be generated in diverse bacterial species. A single round of genome shuffling via protoplast fusion was performed between closely related strains of **a** *B. velezensis* and **b** *C. thermocellum*. For *B. velezensis*, strain GB03 was crossed with a double-resistant auxotrophic mutant of S4, and Δ*metE*::*erm* progeny were selected. The blue bars indicate sequences recombined from S4, with the remaining genomic sequence derived from GB03. For *C. thermocellum*, a double-resistant auxotrophic strain of DSM1313 was crossed with wild-type S14, and *pyrF*^+^ Δ*trpC*::*cat* progeny were selected. The orange bars indicate genomic regions recombined from S14, with the remaining genomic sequences derived from DSM1313. **c** A double-resistant auxotrophic strain of *N. aromaticivorans* F199 was crossed with wild type B0695 via protoplast fusion, and Δ*hisB*::*kan leuB*^+^ progeny were selected. The pink bars indicate sequences recombined from B0695, with the remaining genomic sequences derived from F199. **d** *S. stutzeri* DSM4166 was transformed with genomic DNA from a double-resistant auxotrophic mutant of A1501, and double-resistant auxotrophic recombinants were selected. The green bars indicate sequences recombined from A1501 via natural competence, with the remaining genomic sequences derived from DSM4166. Each concentric circle represents a different resequenced recombinant strain from the four crosses. Blue, orange, pink, and green arrows indicate the locations of the selection markers. Black arrows indicate the origin of replication.

To assess whether our QTL mapping method could be broadly applied across diverse bacterial species, we attempted to develop genome shuffling methods in different Gram-positive and Gram-negative non-model bacteria of interest for environmental and industrial applications. In each case, we selected parental strains with ~98–99% average nucleotide identity in shared genes, performed a single round of genome shuffling and evaluated genome-wide recombination in the progeny (Fig. 5). Similar to genome shuffling in *B. subtilis*, we replaced amino acid biosynthesis genes with antibiotic resistance markers in the respective parents to allow flexible selection of inter-strain recombinants.

We successfully adapted the protoplast recombination method to other Gram-positive bacteria, including *B. velezensis*, a rhizosphere plant growth-promoting bacterium, and *Clostridium thermocellum*, an anaerobic thermophilic cellulose degrader (Fig. 5a, b). Protoplast fusion in these species promoted genome-wide unselected recombination, demonstrating that this strategy could be applied for creating recombinant libraries for genetic mapping in a variety of Gram-positive bacteria. The protoplast fusion method could also be applied to the Gram-negative *Novosphingobium aromaticivorans*, a soil bacterium capable of degrading a wide range of aromatic compounds. We crossed two *Novosphingobium* strains and isolated recombinant progeny (Fig. 5c). While the shuffled strains showed recombination in a ~200 kb region surrounding the selected marker, this capability could be leveraged to create sufficient genome-wide recombination for genetic mapping using several selection markers distributed across the genome.

The use of protoplast fusion methods in a broad range of Gram-negative bacteria can be potentially challenging due to the complexity of their cell walls, which makes protoplasting less efficient. Therefore, we explored alternative methods to generate genome-wide recombination. Large-scale inter-strain genetic exchange in Gram-negative bacteria can be achieved through approaches like chromosomal conjugation^32,33^. In addition, inter-strain genetic diversity could be introduced through the transformation of genomic DNA in naturally competent bacteria^34,35^. Using this approach, we generated recombinant progeny in *Stutzerimonas stutzeri*, a Gram-negative nitrogen-fixing bacterium (Fig. 5d). Natural transformation yielded multiple unrelated recombination events across the genome, indicating that this method can be applied iteratively to generate genetically diverse mapping populations.

Collectively, our results demonstrate that bacterial QTL mapping populations can be generated in both Gram-positive and Gram-negative species using different recombination and selection strategies.

## Conclusion

The power of phenotype-genotype association studies in bacteria is limited by the relatively low recombination rates in natural bacterial populations. To overcome these limitations, we developed an approach to generate highly recombinant genotype panels, enabling high-confidence genetic mapping. We detected QTLs spanning on average ~15 kb, though our data suggest that genetic mapping resolution in bacteria can be improved either by increasing the number of recombinant strains in the QTL population or the overall recombination frequency across the genome.

In contrast to traditional loss-of-function and gain-of-function genetic methods, our approach enables rapid detection of the effects of natural genetic variation in both coding and noncoding regions on bacterial phenotypes, beyond gene presence or absence. Methods such as adaptive laboratory evolution (ALE) and MAE generate genetic diversity in genes and regulatory regions that can be linked to bacterial traits^21,36^. However, ALE experiments are typically applied to targeted phenotypes under selective pressure, and MAE is largely limited to the transfer of single continuous chromosomal segments. An advantage of bacterial QTL mapping is that a single recombinant panel can be applied to any unselected or selected phenotype of interest, and that multiple unlinked recombination events across the chromosome of individual strains allow detection of epistatic interactions on a genome-wide scale. A limitation of our approach is the initial investment required to generate mapping populations with sufficient genome-wide recombination and scale for genetic mapping. Nonetheless, bacterial QTL mapping, in combination with the traditional deletion and pooled genetic screens, provides a powerful platform technology for comprehensive functional bacterial genomics.

## Method

### Strains and culture conditions

Bacterial strains used in this study are listed in Supplementary Table 1. *B*. *subtilis* subsp. *subtilis* RO-NN-1^37,38^, *B*. *subtilis* subsp. *subtilis* NCIB 3610 ^39,40^*, B. subtilis* DK1042^24^, BKK34900^7^, BKE13180^7^*, B. spizizenii* TU-B-10 ^37,41,42^, *B. velezensis* FZB42^43,44^, and *B. velezensis* GB03^45^ were obtained from the Bacillus Genetic Stock Center (BGSC). *B. subtilis* DSM1970 was obtained from the German Collection of Microorganisms and Cell Cultures (DSMZ). *E*. *coli* DH10B and JM109 were used for plasmid construction and propagation. Unless otherwise indicated, *E. coli, B. subtilis* and *B. velezensis* strains were grown in Lysogeny broth (LB) (casein peptone 10 g/l, NaCl 10 g/l, and yeast extract 5 g/l) or LB broth solidified with 1.5% (w/v) Bacto agar. *C*. *thermocellum* cultures were grown in complex CTFUD or chemically defined CTFUD-NY medium^46^. *N*. *aromaticivorans* strains were grown in R2A medium or minimal 457 medium (DSMZ) with 0.2% glucose as a sole carbon source. *S*. *stutzeri* strains were grown in LB broth or chemically defined minimal succinate medium^47^. *E. coli* cultures were grown at 37 °C, *B. subtilis* and *B. velezensis* cultures were grown at 30 °C or 37 °C, *C. thermocellum* cultures were grown at 50 °C or 60 °C, *N. aromaticivorans* strains were grown at 30 °C and *S. stutzeri* strains were grown at 37 °C. Growth media were supplemented with antibiotics, when appropriate, as follows: kanamycin (50 μg/ml), erythromycin (20 μg/ml) plus lincomycin (12.5 μg/ml), chloramphenicol (25 μg/ml for *E. coli*, 20 μg/ml for *B. velezensis*, and 15 μg/ml for *B. subtilis* and *N. aromaticivorans*), carbenicillin (100 μg/ml), spectinomycin (100 μg/ml), neomycin (300 μg/ml), thiamphenicol (10 μg/ml), apramycin (50 μg/ml) and streptomycin (100 μg/ml). All plasmids and oligonucleotides used for strain construction and verification are summarized in Supplementary Tables 2 and 3.

### Construction of a *B. subtilis* biparental mapping population

Genetically diverse recombinant strains for QTL mapping were created by three and four rounds of genome shuffling through protoplast fusion using as parental strains *B. subtilis* RO-NN-1 and DK1042. The process of construction of the QTL population is illustrated in Supplementary Fig. 1. Protoplasts were prepared, fused, and regenerated as described previously^22^. Cells for genome shuffling were grown overnight in selective media at 37 °C. The following day, cultures were diluted 1:100 in fresh media and grown until an OD_600_ of 0.4–0.6. Cells from 5 ml cultures were harvested by centrifugation (8000 × *g*, 5 min, room temperature) and washed three times with 1 ml SMM buffer (0.5 M sucrose, 20 mM MgCl_2_, and 20 mM maleic acid)^48^. Washed cells were resuspended in 1 ml SMMD buffer (SMM buffer supplemented with 5 μg/ml DNase I) containing 1 mg/ml lysozyme, followed by incubation for 1 h at 37 °C. After protoplasting, 500 μl of each parental cell line were mixed, and cells were harvested by centrifugation (2000 × *g*, 20 min, 12 °C). The mixed protoplast pools were washed once in SMMD buffer, resuspended in 400 μl Protoplast fusion buffer (SMM buffer supplemented with 35% polyethylene glycol (PEG) 6000 and 10 mM CaCl_2_), and incubated for 20 min at room temperature^49^. Protoplasts were then washed in SMMD buffer, resuspended in 100 μl SMMD buffer supplemented with 1% bovine serum albumin (BSA), and spotted onto DM3 regeneration medium^50^ without selection. The following day, cells were scraped from the regeneration plates, resuspended in minimal medium (MM)^51^ and plated on selective LB or MM plates for the isolation of inter-strain recombinants.

In the first round of shuffling (S1), strain RO-NN-1 was crossed with DK1042 Δ*hisB*::*kan* Δ*metE*::*erm* (“DK1042 HK ME”) and strain DK1042 was crossed with RO-NN-1 Δ*hisB*::*kan* Δ*metE*::*erm* (“RO-NN-1 HK ME”). Δ*hisB*::*kan metE*^+^ (“S1 HK”) and Δ*metE*::*erm hisB*^+^ (“S1 ME”) recombinant progeny were selected on minimal medium (MM) agar supplemented with histidine (300 μM) and kanamycin and MM agar supplemented with methionine (1 mM), erythromycin and lincomycin, respectively. S1 HK and S1 ME pools were made by scraping all colonies off the selective plates. The cells were resuspended in 500 μl ddH_2_O, 5 μl of each cell suspensions were then inoculated into 5 ml selective MM and grown overnight at 37 °C. Pools were stored as glycerol stocks. Select individual strains from the S1 HK and S1 ME pools were resequenced, and RO-NN-1 was identified as the major contributor to the offspring genomes.

In the second round of shuffling (S2), the S1 HK pool was crossed with DK1042 Δ*metE*::*erm* (“DK1042 ME”), the S1 ME pool was crossed with DK1042 Δ*hisB*::*kan* (“DK1042 HK”), and the S1 HK was crossed with S1 ME. Prototrophic (*hisB*^*+*^ *metE*^*+*^, “WT”) and double-resistant progeny (Δ*hisB*::*kan* Δ*metE*::*erm*, “HK ME”) from each cross were selected on MM agar and LB agar supplemented with kanamycin and erythromycin, respectively. WT and HK ME pools were created from each cross as described above and stored as glycerol stocks. Individual strains from each pool were resequenced. The number of genetic variants per strain was estimated using the single-nucleotide polymorphism (SNP) call function of Geneious Prime (v2019.0 and v2024.0) and the RO-NN-1 reference genome. S2 WT and S2 HK ME strains resulting from the S1 ME x DK1042 HK cross showed the most genetic diversity and were selected for the next round of shuffling.

In the third round of shuffling (S3), the S2 WT pool was crossed with DK1042 HK ME, giving S3 HK1 and S3 ME1 recombinant progeny, and the S2 HK ME pool was crossed with DK1042, giving S3 HK2 and S3 ME2 shuffled strains. All S3 HK1 and S3 HK2 strains were combined to create the S3 HK pool.

In the fourth round of shuffling (S4), the S3 HK pool was crossed with strain RO-NN-1 ME, giving S4 HK ME recombinant progeny.

Five hundred seventy-three individual S3 HK1, S3 HK2, and S3 ME1 strains and 50 S4 HK ME strains were arrayed in 96-deep-well plates, and their genomes were resequenced using short-read sequencing. Detailed analyses of these genome sequences revealed that approximately 10% of the samples contained mixed reads, with genetic variants from more than one strain at the same positions, suggesting that they did not represent pure cultures. To resolve this issue, cultures from all wells were streaked to single colonies on selective plates. A total of 382 individual shuffled strains, 48 random replicates and parental controls were arrayed in five 96-deep-well plates (Supplementary Fig. 3), and their genomes were resequenced. SNPs were identified, and boundaries of the genomic fragments inherited from each parent were determined as described below. Nearly identical strains, rare genetic variants and plate-specific variants were filtered out, resulting in a final set of 350 genetically diverse strains used for genetic mapping.

### Triparental genome shuffling

Triparental crosses were carried out by protoplast fusion as described above. A pool of 24 S4 HK ME strains from the RO-NN-1 × DK1042 biparental cross was shuffled with wild-type *B. spizizenii* TU-B-10. Strains from both potential recombinant genotypes, either Δ*hisB*::*kan metE*^*+*^ (“HK”) or Δ*met*::*erm hisB*^*+*^ (“ME”), were selected on MM agar supplemented with histidine (300 μM) and kanamycin and MM agar supplemented with methionine (1 mM), erythromycin, and lincomycin, respectively. Individual recombinant strains were isolated and resequenced.

### Genome shuffling of *B. velezensis* strains

Protoplast formation, fusion and regeneration of *B. velezensis* GB03 and S4 Δ*hisB*::*kan* Δ*metE*::*erm* cells for genome shuffling was performed as described previously^22^, with some modifications. Cells were grown in selective LB broth overnight, diluted 100-fold in selective minimal medium^51^, and incubated at 37 °C overnight. The following day, cells from 10 ml cultures adjusted to an OD_600_ of 3 were harvested by centrifugation (7000 × *g*, 5 min, room temperature). Pellets were then washed three times in 1 ml of SM buffer I (1.4% K_2_HPO_4_, 0.5% KHPO_4_, 0.1% sodium citrate, 0.2% (NH_4_)_2_SO_4_ and 0.02% MgSO_4_). Washed cells were resuspended in 1 ml SM buffer II (0.5 M sucrose, 20 mM maleic acid, 20 mM MgCl_2_ and 5 µg/ml DNase I in SM buffer I) supplemented with 10 mg/ml lysozyme, followed by incubation for 1 h at 37 °C. Cells were then washed twice in SM buffer II. Five hundred microliters from each parental cell line were mixed and centrifuged (1000 × *g*, 5 min, room temperature). The mixed protoplast pool was resuspended in Fusion buffer (35% PEG 6000) and incubated at 37 °C for 10 min, followed by an additional 5-min incubation step at room temperature. Protoplasts were washed in SM buffer II and resuspended in SM buffer II containing 1% BSA. The resulting protoplast suspension was spotted on DM3 regeneration medium^50^ without antibiotic selection and incubated overnight at 37 °C. The following day, lawns of regenerated cells were scraped from the regeneration plates, suspended in SM buffer I and plated on minimal medium supplemented with 1 mM methionine and erythromycin for isolation of Δ*met*::*erm* inter-strain recombinants.

### Genome shuffling of *C. thermocellum* strains

*C. thermocellum* DSM1313 *pyrF*::*neo trpC*::*cat* and S14 cells for genome shuffling were grown overnight in CTFUD with appropriate selection at 60 °C. The resulting precultures were then inoculated into 10 ml of fresh CTFUD and incubated overnight at 60 °C. Cells were harvested by centrifugation (7000 × *g*, 5 min, room temperature). Pellets were then washed with 5 ml TM buffer (0.1 M Tris-HCl, 20 mM MgCl_2_, pH 7.5) and resuspended into 1 ml TMS buffer (TM buffer supplemented with 0.5 M sorbitol, 10 µg/ml DNase I and 1 mg/ml lysozyme). The resulting cell suspensions were incubated for 1 h at 50 °C. Protoplasted cells from the parental strains were then combined at 1:1 ratio, and the mixtures were harvested by centrifugation (1000 × *g*, 10 min, room temperature). Pellets were washed with 1 ml of TMS buffer and resuspended in 100 µl of TMS buffer. Protoplast fusion was initiated by adding 400 µl of Fusion buffer (35% PEG 6000, 0.1 M Tris-HCl, 20 mM MgCl_2_, 20 mM CaCl_2_, 0.5 M sorbitol, pH 7.5), followed by incubation for 20 min at room temperature. Then, 900 µl of TMS buffer was added to each sample, cells were harvested by centrifugation (3000  × *g*, 10 min, room temperature), washed with 1 ml TMS buffer, and resuspended in 200 µl of TMS buffer. Cell suspensions were plated in molten CTFUD agar supplemented with 0.1 M sorbitol and 0.02% BSA and incubated for 3 days at 60 °C. The agar was then excised, transferred to a tube containing 5 ml TMS buffer, and vortexed to resuspend the cells. Cells were then washed with TMS buffer and plated in molten CTFUD-NY agar supplemented with 0.05 mM tryptophan and thiamphenicol to select for *pyrF*^+^ Δ*trpC*::*cat* inter-strain recombinants and counterselect against the Δ*trpC*::*cat* Δ*pyrF*::*neo* auxotrophic parent.

### Genome shuffling of *N. aromaticivorans* strains

*N. aromaticivorans* F199 *hisB*::*kan leuB*::*cat* and B0695 strains were grown overnight in 457 medium supplemented with 0.2% glucose at 37 °C, with the appropriate amino acid supplementation and antibiotic selection. The following day, cells were pelleted (1000 × *g*, 10 min, room temperature) and washed twice with 100 mM CaCl_2_ to remove residual antibiotics, then resuspended in CaCl_2_, and incubated on ice for 2 h. Cells were pelleted (1000 × *g*, 10 min, room temperature) and resuspended in Incubation medium (100 mM Tris-HCl, 10 mM glycerol, pH 8.0)^52^ containing 0.1 mg/ml lysozyme, followed by incubation at 37 °C for 20 min. Next, 0.4 mM EDTA, pH 8.0, was added to each sample, and the mixtures were incubated for an additional 20 min at room temperature. Fifty millimolar MgCl_2_ was added to each cell suspension. Roughly equivalent amounts (based on OD_600_) of cells from each strain were then mixed, pelleted, and resuspended in 100 µl of Incubation medium containing 20 mM MgCl_2_ and 35% PEG 6000, followed by incubation at room temperature for 5 min. One milliliter of Incubation medium containing 20 mM MgCl_2_ was added to each sample, and cells were pelleted by centrifugation (1000 × *g*, 10 min, room temperature). The pellets were then resuspended in 2% BHI containing 20 mM MgCl_2_ and incubated at room temperature for 1 h. Cells were then spotted onto the surface of a 2% BHI agar plate containing 20 mM MgCl_2_ and incubated at 37 °C for 24 h. Following incubation, cells were scraped off the surface of the agar plate and resuspended in carbon-free 457 media. The resuspended cells were washed in carbon-free 457 media for a minimum of three times to remove nutrient carryover, then plated on 457 agar supplemented with histidine and kanamycin to select for Δ*hisB*::*kan* inter-strain recombinants and counterselect against the leucine auxotrophic parent F199 Δ*hisB*::*kan* Δ*leuB*::*cat*.

### Genome shuffling of *S. stutzeri* strains

*S. stutzeri* DSM4166 cells for genome shuffling were grown overnight in LB at 37 °C. A 500 μl aliquot of the overnight culture was then inoculated in 25 ml of fresh Competence medium (LB, 10 mM Tris-HCl, pH 7.5, 0.2% glucose and 1 mM MgSO_4_) and grown to early stationary phase. Cells from 5 ml culture were harvested by centrifugation (7000 × *g*, 10 min, room temperature). The pellet was then resuspended in 200 μl of LB and mixed with 8 μg of genomic DNA from strain A1501 Δ*trpC*::*apr* Δ*hisB*::*kan*. The cell suspension was spotted onto a LB agar plate and incubated for 24 h at 37 °C. Cells were scraped off the plate, resuspended in LB and plated on LB agar supplemented with apramycin and kanamycin to select for Δ*trpC*::*apr* Δ*hisB*::*kan* inter-strain recombinants.

### Sequencing

Genomic DNA from the *B. subtilis* QTL panel was extracted using the Mag-Bind® Bacterial DNA 96 Kit (Omega Bio-Tek, Norcross, GA, USA) following the manufacturer’s instructions. DNA was then sequenced by the Joint Genome Institute (JGI) using short-read sequencing.

### Variant calling

Variant calling for the whole-genome resequencing data was performed as described previously^22^. Briefly, raw sequence data with an average sequencing depth of 80× to 100× were processed by trimming low-quality bases based on Phred quality scores using Trimmomatic, such that any leading or trailing sequence bases of the sequence read with Phred quality score less than 3 were removed, and the read less than 70 bases long were discarded (Trimmomatic v0.39^53^). The trimmed reads were aligned to the reference genome using the BWA-MEM algorithm (BWA v0.7.17^54^), and the resulting alignments were converted to compressed BAM files using SAMtools (SAMtools v1.3.1^55^). Genomic coordinates were sorted using Picard Tools (Picard v2.27.4, Broad Institute) and indexed with SAMtools^55^. SNPs were called using the GATK HaplotypeCaller (GATK v4.1.8.1^56^). Hard-filtering criteria, as recommended by the GATK developers, were applied to remove low-quality and potentially biased variants. The following filters were used: QD < 2.0, QUAL < 30.0, SOR > 3.0, FS > 60.0, MQ < 40.0, MQRankSum < −12.5, and ReadPosRankSum < −8.0. Variants failing to meet these criteria were excluded from further analysis.

Genomes of *B. subtilis* (RO-NN-1 × DK1042) S4 HK ME × TU-B-10 shuffled strains were analyzed using Geneious Prime (v2024.0.7). Sequence reads were mapped to the RO-NN-1, NCIB3610, and TU-B-10 reference genomes. SNPs were identified, and boundaries of the genomic fragments inherited from each parent were determined.

Genomes of *B. velezensis* GB03 × S4 Δ*hisB*::*kan* Δ*metE*::*erm* strains were analyzed using Geneious Prime (v2024.0.7). Sequence reads were mapped to the GB03 and S4 reference genomes. SNPs were identified, and boundaries of the genomic fragments inherited from each parent were determined.

Genomes of *C. thermocellum* DSM1313 Δ*pyrF*::*neo* Δ*trpC*::*cat* × S14 shuffled strains were analyzed using Geneious Prime (v2024.0.7). Sequence reads were mapped to the DSM 1313 and S14 reference genomes. SNPs were identified, and boundaries of the genomic fragments inherited from each parent were determined.

Genomes of *N. aromaticivorans* F199 Δ*hisB*::*kan* Δ*leuB*::*cat* × B0695 shuffled strains were analyzed using Geneious Prime (v2024.0.7). Sequence reads were mapped to the F199 and B0695 reference genomes. SNPs were identified, and boundaries of the genomic fragments inherited from each parent were determined.

Genomes of *S. stutzeri* DSM4166 × A1501 Δ*trpC*::*apr* Δ*hisB*::*kan* shuffled strains were analyzed using Geneious Prime (v2024.0.7). Sequence reads were mapped to the DSM4166 reference genomes. SNPs were identified, and boundaries of the genomic fragments inherited from each parent were determined.

### Assignment of recombination segment boundaries

Recombination regions were determined based on the shortest distance between recombined variants. Regions encompassing DNA sequences between at least two consecutive DK1042-derived (biparental and triparental crosses) or TU-B-10-derived (triparental crosses) SNPs were defined as recombination fragments. The same criteria were applied to shuffled strains of *B. velezensis*, *C. thermocellum*, *N. aromaticivorans,* and *S. stutzeri*.

### Phenotypic data preparation and heritability estimation

Phenotypic data were initially screened for outliers using the median absolute deviation (MAD) method. Any data points with a MAD distance greater than 6 were classified as outliers and excluded from all subsequent analyses.

To assess the genetic control of traits and the repeatability of measurements, broad-sense heritabilities (*H*^2^) were estimated. The heritability was calculated using the following formula:

1 $${H}^{2}=\frac{{\sigma }_{G}^{2}\,}{{\sigma }_{G}^{2}\,+\,{\sigma }_{\epsilon }^{2}}$$

Where, ${\sigma }_{G}^{2}$ represents the genotypic variance, attributed to differences between bacterial strains, and ${\sigma }_{\epsilon }^{2}$ represents the residual variance.

Additionally, to adjust for potential confounding due to temporal variation, the time at which measurements were conducted was included as a covariate in the statistical model to account for batch effects.

The random effects of bacterial strains (genotypes) were modeled using best linear unbiased predictors (BLUPs) for each phenotypic trait. These BLUPs were subsequently used in all genome-wide QTL analyses.

### Heritability visualization

Genetic maps of shuffled strains were derived using a Python codebase that can be found at https://github.com/LaneMatthewJ/heritability_illustrator/. In the Python codebase, the code was designed to analyze a user-specified region of genetic data filtered by a start and end position within the genome.

The supplied VCF file was read in as a Pandas Dataframe^57,58^. All SNPs between the loci range of 0 and 4.2 Mb were selected, such that all SNPs within the genome were maintained. All genomic information was transformed to a “major parent match” Boolean, such that if the SNP in the progeny sample matched the SNP in the major parent, the value was converted to 1, otherwise 0. No samples were clustered. All unique samples were plotted as a heatmap using the Seaborn heatmap plot^59^, such that each row represents an individual sequenced shuffled strain, and each column a genetic variant.

### QTL mapping

Association mapping on the phenotypic traits was conducted using the following mixed linear model implemented in the GAPIT package in R^60^:

2 $$Y=X\beta+{Zu}+\,\epsilon$$

where, *Y* is the *n* × 1 vector of phenotypic values, with *n* sample size, *β* is the *p* × 1 vector of fixed additive SNP effects, with *X* as its corresponding *n* × *p* incidence matrix of genotypic data, *u* represents the *n* × 1 vector of random effects due to genotypes, which includes the genetic relationship matrix, estimated using Zhang method^61^ (Supplementary Fig. 4), *Z* is the incidence matrix for random effects, and *ϵ* is the *n* × 1 vector of residuals accounting for unexplained phenotypic variance.

A total of 59,571 high-quality SNPs with a minor allele frequency of at least 0.03 were used in the association analysis, derived from 350 unrelated bacterial strains. The SNP dataset was filtered to remove highly clonal individuals.

The genetic relationship matrix was used to hierarchically cluster the 350 shuffled strains into 23 diverse groups using the hclust function in R. One strain from each group was then randomly selected to assess phenotypic variability across different traits.

### Spore preparation

Spores for spore germination assays were routinely produced using the nutrient exhaustion method in liquid Difco sporulation medium (DSM)^62^. Strains were grown overnight in 96-deep-well plates at 37 °C in LB broth supplemented with antibiotics where necessary. Cells were then washed with 1× phosphate-buffered saline (PBS, pH 7.4), pellets were resuspended in DSM, and cultures were incubated at 37 °C for 72 h. Spores were harvested by centrifugation (1000 × *g*, 15 min, room temperature) and washed five times with 1× PBS buffer. Spore suspensions were stored in 1× PBS at 4 °C until analysis.

### Spore germination assay

Spore germination assays were carried out as described previously^23^, with some modifications. Spores were normalized to an optical density at 480 nm (OD_480_) of 0.6 in ddH_2_O, and 200 µl spore suspensions were then transferred to clear, flat-bottom, 96-well plates. After heat activation at 70 °C for 30 min, spores were cooled at 4 °C for 15 min and harvested by centrifugation (1000 × *g*, 15 min, room temperature). The supernatants were discarded, and the pellets were resuspended in 10 mM Tris-HCl, 20 mM KCl, pH 7.4 (buffer) or buffer supplemented with nutrient germinants (1 or 50 mM L-alanine, 1 or 50 mM L-valine). Germination assays were carried out at 37 °C, and the OD_480_ was monitored every 2 min for 2 h using a Synergy H1 plate reader (BioTek, Winooski, VT, USA) with Gen 5 software (v3.03).

### Microscopy

Spores were concentrated by centrifugation and then immobilized on agarose pads (1.5% (w/v) agarose in 1× PBS buffer). Phase-contrast microscopy was performed using an Axio Imager.A2 Zeiss microscope (Carl Zeiss, Oberkochen, Germany) equipped with Zeiss Plan-Apochromat 100×/1.4 Oil Ph3 immersion objective lens (Carl Zeiss, Oberkochen, Germany) and Canon EOS Rebel T1i camera (Canon, Tokyo, Japan). Image processing was performed using EOS Utility 2 software (Canon, Tokyo, Japan). For each strain, at least three independent spore preparations were subjected to microscopy and representative images are shown.

### Swarm expansion assay

Swarming motility assays were conducted as described previously^63^, with some modifications. Cultures of *B. subtilis* strains were inoculated in LB broth supplemented with antibiotics as needed and grown overnight in a shaking incubator at 37 °C and 250 rpm. The overnight cultures were subcultured into fresh LB in culture tubes or 96-deep-well plates and grown to mid-log phase (OD_600_ ~0.5) at 37 °C and 250 rpm. Cells were then harvested by centrifugation and resuspended to an OD_600_ of 0.6 in 1× PBS buffer containing 0.5% India ink (Higgins). LB containing 0.7% agar was prepared freshly on the day of the experiment in clear, flat-bottom, 6-well plates (5 ml agar per well), and the plates were dried open-faced in a laminar flow hood for 30 min. One microliter of resuspended cells was spotted onto the center of an agar well, allowed to dry for 20 min and incubated at 37 °C for 5 h. For experiments with exogenous surfactin, surfactin (cat#S3523, Sigma-Aldrich, St. Louis, MO, USA) was dissolved in pure ethanol and added to the swarm agar wells at a final concentration of 12.5 μM.

### Biofilm formation

Colony biofilm formation of *B. subtilis* strains was induced using MSgg agar medium (5 mM potassium phosphate (pH 7.0), 100 mM MOPS (pH 7.0), 2 mM MgCl_2_, 700 μM CaCl_2_, 50 μM MnCl_2_, 50 μM FeCl_3_, 1 μM ZnCl_2_, 2 μM thiamine, 0.5% glycerol, 0.5% glutamate, 1.5% (w/v) Bacto agar)^64^, supplemented with amino acids for auxotrophic strains. Cultures of *B. subtilis* strains were inoculated in LB broth and grown overnight in a shaking incubator at 37 °C and 250 rpm. The overnight cultures were subcultured into fresh LB in culture tubes or 96-deep-well plates and grown to mid-log phase (OD_600_ ~0.5) at 37 °C and 250 rpm. MSgg agar was prepared in clear, flat-bottom, 6-well plates (6 ml agar per well). One microliter of mid-log culture was spotted onto the center of an agar well, allowed to dry for 20 min and incubated at 30 °C for 72 h.

### Swarm and biofilm image acquisition

A custom robot was designed and 3D printed to automate the collection of high-resolution image data of swarm expansion assays and biofilms on 6-well plates (manuscript in preparation). The system used microcontrollers to move clear 6-well laboratory plates under a fixed camera in uniform lighting conditions. Each well was systematically positioned under the camera and illuminated using a dark-field approach, in which a strip of white LED lights illuminated the bacterial colony from an angle and did not shine directly into the camera. The system was operated by a computer (connected via USB) and automatically tracked samples using manual-input sample IDs, barcode IDs, and/or radio frequency IDs. The system supports two cameras: first a low-resolution camera (Hotpet 8MP Optical 10X Zoom 5–50 mm Lens Industrial Web Camera, Hotpet, China) which was used to image the population of *B. subtilis* strains, and was later upgraded to a high-resolution camera (Canon EOS 4000D, Canon, Tokyo, Japan) used to conduct the validation of the swarming phenotype and image biofilm colony morphologies.

### Swarm image analysis

Swarm assay phenotyping was formulated as a semantic segmentation problem, in which manually-annotated *B. subtilis* swarm images were used to train a region growing convolutional neural network (CNN) model based on ref. ^65^ to identify pixels along the boundaries of the swarm (Supplementary Fig. 29). Each image was stored in PNG format with a spatial resolution of 896 × 1152 pixels. A training set of 60 images was manually annotated using the GNU Image Manipulation Program^66^, in which we manually traced the boundary of each colony and stored the contour as a black-and-white PNG image. The annotated images and masks were then randomly partitioned into 80% training and 20% validation sets. Each image was further broken into smaller training tiles of size 128 × 128, which were augmented during training using random flips, rotations, Gaussian blur, and color jitter corresponding to brightness, contrast, hue, and saturation. The CNN model was trained to predict the class labels (i.e., boundary vs. background) of the center 3 × 3 square of each tile using a batch size of 512, the focal loss objective function^67^, and Adam optimizer^68^ with default hyperparameters. Early stopping of 20 epochs was used to ensure model convergence and prevent overfitting to the training set. The model weights from the epoch that minimized validation error were selected as the final weights used for inference.

Inference was formulated as a region growing task following^65^, where CNNs iteratively add pixels to a growing colony boundary segmentation. Swarms with high-contrast boundaries were accurately detected, but swarms with low-contrast and diffuse boundaries (especially close to the edge of the well) could result in false positive and false negative predictions. Thus, we developed a segmentation post-processing procedure that detected the longest continuous boundary from a swarm and used the segment to compute the best-fit circle. Note that for swarms with contiguous boundaries, the whole boundary was used as the line segment. Each circle (parameterized by the radius and the row and column location of the center) was optimized by minimizing the Chamfer distance between the circle and the line segment. The best-fit circle was then used to predict the swarming phenotypes (i.e., colony radius and area). A conversion factor based on the size of the 6-well plate was used to convert between pixel units and millimeters. Each result (predicted boundary and best fit circle) was manually inspected for accuracy. Any images with errors were manually corrected and phenotyped.

### Reconstruction of causal genetic variants

To identify and validate causal genetic variants, we functionally profiled regions within each QTL through CRISPR-Cas9-mediated genetic swaps using pJOE9658.1, which carries the *cas9* gene under the control of tetracycline-inducible *tetLM* riboswitch and a single-guide RNA (sgRNA) encoding sequence transcribed from the strong semisynthetic promoter P_VanP*_^69^. Alleles in strain DK1042 were replaced with the alternative variants of these alleles from strain RO-NN-1. Twenty-nucleotide sgRNA spacer sequences targeting the regions of interest were designed using the in-build feature “find CRISPR sites” in Geneious Prime (v2023.0 and v2024.0). The reference genome of strain RO-NN-1 was used as an off-target database. sgRNA spacer sequences were cloned into the BsaI sites of pJOE9658.1 by ligating annealed oligonucleotides^69,70^. Oligonucleotides were designed to include sequences that are complementary to the sticky ends generated by BsaI digestion of the plasmid. Repair templates were generated by PCR amplification of suitable regions from RO-NN-1 genomic DNA. The repair templates were then cloned between the SfiI sites of pJOE9658.1 or cotransformed with the plasmids as PCR products. All pJOE9658.1 derivative constructs were verified by next-generation whole-plasmid sequencing (Plasmidsaurus Inc., Eugene, OR, USA).

Strain DK1042 was transformed with the pJOE9658.1 derivative plasmids and repair templates by natural competence, following standard protocols^71^. Transformants were plated on LB agar plates supplemented with 50 µg/ml kanamycin and 200 ng/ml anhydrotetracycline (to induce Cas9 expression) and incubated for 24 h at 30 °C. The resulting colonies were streaked on LB agar without antibiotics and incubated for 24 h at 45 °C to cure the plasmid. Single colonies were then tested for the loss of the plasmid by patching on LB agar plates with kanamycin. The colonies that failed to grow on these plates were analyzed for the presence of the desired edits using PCR amplification of the targeted region, followed by long-read sequencing (Plasmidsaurus Inc., Eugene, OR, USA). Select strains were also verified by whole-genome sequencing on an Illumina MiSeq instrument (Illumina, San Diego, CA) as described previously^22^. Nextera XT libraries (Illumina, San Diego, CA) were generated from genomic DNA according to the manufacturer’s protocol (15031942 v05). Libraries were validated on an Agilent Bioanalyzer (Agilent, Santa Clara, CA) using a DNA7500 chip and concentration was determined on an Invitrogen Qubit (Waltham, MA) with the broad range double-stranded DNA assay. Barcoded libraries were pooled and prepared for sequencing following the manufacturer’s recommended protocol (15039740 v10, Standard Normalization). One paired-end sequencing run (2 × 301) was completed on an Illumina MiSeq instrument (Illumina, San Diego, CA) using v3 chemistry. Recombination fragment boundaries were determined as described in the *Assignment of recombination segment boundaries* section. When the first and/or the last SNP within a gene was recombined, the entire region between that SNP and the start and/or stop codon was considered part of the recombined region.

### Transformation frequency assays

Competent cells for transformation frequency assays were prepared as described previously^71^, with some modifications. *B. subtilis* strains were grown overnight in 1 ml LB broth in a shaking incubator at 37 °C and 250 rpm. Cells were then harvested by centrifugation, washed in 1 ml 1× PBS, and 200 μl cell suspension was inoculated in 3 ml starvation medium 1 (SM1) containing 0.2% (NH_4_)_2_SO_4_, 1.4% K_2_HPO_4_, 0.6% KH_2_PO_4_, 0.07% sodium citrate, 0.5% glucose, 0.02% MgSO_4_·7H_2_O, 0.2% yeast extract, 0.025% casamino acids. Following incubation for 3 h or for the indicated time period at 37 °C, 3 ml of starvation medium 2 (SM2) containing 0.2% (NH_4_)_2_SO_4_, 1.4% K_2_HPO_4_, 0.6% KH_2_PO_4_, 0.07% sodium citrate, 0.5% glucose, 0.02% MgSO_4_·7H_2_O, 0.1% yeast extract, 0.01% casamino acids, and 0.05% CaCl_2_ was added, and cells were further incubated for 90 min under the same conditions. The cultures were then combined with plasmid DNA at the indicated concentrations and incubated for an additional 1 h. Next, 3 ml of LB was added to the cultures and the cells were incubated for 30 min. The cells were harvested by centrifugation and resuspended in 1× PBS. Serial dilutions were prepared in 1× PBS, and cells were plated onto selective LB plates to determine the number of transformants and onto LB plates to determine the number of viable cells. The transformation frequency was determined by calculating the ratio of transforming units to colony-forming units.

### CRISPR-Cas9 editing frequency assay

CRISPR-Cas9 editing efficiency was assessed by deleting a segment of the *trpC* locus and estimating the number of tryptophan auxotrophs. Strains RO-NN-1, JMB298, JMB299, and JMB353-375 were grown overnight in 1 ml LB broth in a shaking incubator at 37 °C and 250 rpm. Cells were then harvested by centrifugation, washed in 1 ml 1× PBS, and 200 μl cell suspension was inoculated in 3 ml SM1 medium. Following incubation for 3 h at 37 °C, 3 ml of SM2 medium was added, and cells were further incubated for 90 min under the same conditions. The cultures were then combined with 9 µg of pJM607 (for strains that contained the RO-NN-1 *trpC* allele) or pJM608 (for strains that contained the NCIB3610 *trpC* allele) and incubated for an additional 1 h. Next, 3 ml of LB was added to the cultures and the cells were incubated for 30 min. The cells were harvested by centrifugation and resuspended in 1× PBS. Serial dilutions were prepared in 1× PBS, and cells were plated onto LB plates supplemented with kanamycin and 200 ng/ml anhydrotetracycline to determine the number of transformants and onto LB plates to determine the number of viable cells. Transformants were then patched on MM agar plates with and without tryptophan, and with histidine or methionine, where necessary, for the 23 shuffled strains to estimate the genome editing efficiency.

### Xylanase activity assay

Xylanase activity assays were performed as described previously^72^. Cultures of *B. subtilis* strains were inoculated in LB broth supplemented with antibiotics as needed and grown overnight in a shaking incubator at 37 °C and 250 rpm. The overnight cultures were subcultured into fresh LB in 96-deep-well plates and grown for 7 h at 37 °C and 250 rpm. The xylanase activity assays were performed in 96-well clear flat-bottom plates. In each well, 20 µl of culture supernatant and 20 µl of substrate solution (1% beechwood xylan (Neogen, Bray, Ireland) in 10 mM Tris-HCl, pH 8.0) were added. The reactions were carried out at 40 °C for 1 h. Then, 160 µl of dinitrosalicyclic acid (DNS) solution (0.1% 3,5 dinitrosalicyclic acid, 30% sodium potassium tartrate, pH 7.0) was added to each well, and the microplates were incubated at 100 °C for 20 min in a dry oven. Finally, the plates were analyzed using a plate reader (BioTek, Winooski, VT, USA) with Gen 5 software (v3.03) to determine the optical density of each sample at 570 nm. Standard xylose (cat#X1500 Sigma-Aldrich, St. Louis, MO, USA) solutions were prepared in 10 mM Tris-HCl, pH 8.0, and 40 µl of each standard was analyzed using DNS solution as described above.

### Measurement of pulcherrimin

Pulcherrimin measurements were performed as previously described^73^, with some modifications. *B. subtilis* strains were grown overnight in LB broth in a shaking incubator at 37 °C and 250 rpm. The overnight cultures were then subcultured into 500 µl of modified LBGM medium (LB broth supplemented with final concentrations of 0.25% glycerol (v/v), 25 μM MnSO_4_, and 0.2 mM FeCl_3_)^74^ in culture 96-deep-well plates and incubated for 48 h at 37 °C and 250 rpm. The cultures were then centrifuged (1500 × *g*, 10 min, room temperature) and washed with 500 µl 1× PBS. The resulting pellets were resuspended in 500 µl of 2 M NaOH and incubated for 10 min at room temperature. The cell suspensions were then centrifuged (1500 × *g*, 10 min, room temperature). Following centrifugation, 200 µl of the supernatant were transferred to the wells of a clear, flat-bottom, 96-well plate and OD at 410 nm was measured using a Synergy H1 plate reader (BioTek, Winooski, VT, USA) with Gen 5 software (v3.03). OD at 600 nm was measured for each culture and used to normalize the readings.

### Proteomics

Samples for proteomic analysis were collected from swarms incubated at 37 °C for 5 h on LB containing 0.7% agar in clear, flat-bottom, 6-well plates. Cells were scraped off from the agar surface, resuspended in 1 ml 1× PBS buffer and harvested by centrifugation (1000 × *g*, 15 min, room temperature). The pellets were then flash-frozen and stored at −80 °C until analysis. Cells were prepared for proteomic analysis as previously described^75,76^. Briefly, cells were lysed by bead beating with 0.15 mm zirconium oxide beads in 100 mM Tris-HCl, pH 8.0 (Geno/Grinder 2010, SPEX). The crude lysates were adjusted to 4% SDS and 10 mM dithiothreitol, heated to 90 °C, and cleared by centrifugation. Protein concentrations were measured by a NanoDrop OneC spectrophotometer (Thermo Scientific, Waltham, MA, USA) at 205 nm. Cleared lysates were moved to a new tube, and cysteines alkylated with 30 mM iodoacetamide. Proteins were isolated via the protein aggregation capture method^77^, washed as prescribed, and digested in situ with sequencing-grade trypsin at a 1:75 (w/w) trypsin-to-protein ratio in 100 mM ammonium bicarbonate, pH 8.0, at 37 °C overnight and the following day. The resulting tryptic digests were acidified with formic acid, filtered through a 10 kDa MWCO centrifugal concentrator (Vivaspin 500, Sartorius, Goettingen, Germany), and quantified by NanoDrop OneC. For each sample, 2 µg of peptides were analyzed by automated 1D LC-MS/MS using a Vanquish ultra-high-pressure liquid chromatography (UHPLC) system connected directly to a nanoelectrospray source on a Q Exactive Plus mass spectrometer (Thermo Scientific, Waltham, MA, USA). For each sample, 2 µg of peptides was loaded directly on an in-house pulled nanospray emitter (75 micron ID) packed with 15 cm of Kinetex RP C18 resin (1.7 micron; Phenomenex) and separated by UHPLC along a 180-min organic gradient^75^. Eluting peptides were measured and sequenced by data-dependent acquisition on the MS^78^.

MS/MS spectra were searched against the *B. subtilis* RO-NN-1 and *B. subtilis* NCIB3610 reference proteomes from UniProt (along with common protein contaminants) using the SEQUEST HT algorithm in Proteome Discoverer version 2.5 (Thermo Scientific, Waltham, MA, USA), and peptide spectrum matches (PSMs) were scored and filtered with percolator, controlling the false-discovery rate to <1% at the PSM and peptide levels^79^. Peptides were quantified by the chromatographic area under the curve, mapped to their respective proteins, and summed to estimate protein-level abundance. Raw protein files were exported from Proteome Discoverer. Log2-transformation of protein abundances was performed, followed by local regression (LOESS) normalization and mean-centering across the entire dataset in R using scripts from the InfernoRDN software (v1.1.7995)^80^. The abundance values for proteins with missing values were imputed with random values drawn from the normal distribution (width 0.3, downshift 2.5) using R.

### Quantitative analysis of surfactin production

Samples for analysis of surfactin production were collected from swarms incubated at 37 °C for 5 h on LB containing 0.7% agar in clear, flat-bottom, 6-well plates. Cells were scraped off from the agar surface, resuspended in 1 ml 1× PBS and harvested by centrifugation (1000 x *g*, 15 min, room temperature). The pellets were then flash-frozen and stored at −80 °C until analysis. Cell pellets were then lyophilized until completely dry. A biphasic extraction protocol was performed as previously described^81^. Briefly, equal volumes of ice-cold hydrated ethyl acetate and LC-MS grade water were added to the samples. Samples were vortexed for 1 min and incubated overnight at 4 °C. Following a 10-min centrifugation, the ethyl acetate and aqueous fractions were separated. The aqueous fractions were then filtered using a 10 kDa MWCO centrifugal concentrator (Vivaspin 500, Sartorius, Goettingen, Germany) by centrifugation at 12,000 × *g*. The filtered aqueous extracts were freeze-dried and resuspended in an aqueous solvent (5% acetonitrile, 0.1% formic acid), using 30 µL for cell pellets. The ethyl acetate extracts were air-dried in a chemical fume hood and subsequently resuspended in an organic solvent (70% acetonitrile, 0.1% formic acid) using the same volumes as the aqueous fractions.

Following resuspension, both aqueous and organic extracts were analyzed using untargeted metabolomics via UHPLC-MS/MS to profile metabolite composition. Samples were analyzed using a ThermoFisher Q-Exactive Plus mass spectrometer equipped with an in-house-constructed nanospray analytical column (75 µm × 150 mm) packed with a 1.7-µm C18 Kinetex RP C18 resin (Phenomenex). The mobile phase consisted of solvent A (95% water, 5% acetonitrile, and 0.1% formic acid) and solvent B (70% acetonitrile, 30% water, and 0.1% formic acid). Metabolite separation was achieved using a 30-min linear gradient from 5% aqueous solvent to 100% organic solvent at a flow rate of 250 nL/min, with an injection volume of 10 µL followed by mass spectrometric detection in positive electrospray ionization (ESI+) mode. Full-scan MS spectra were acquired using Xcalibur v4.3 from 135 to 2000 *m*/*z* at 70,000 resolution by employing the top *N* data-dependent acquisition method, where *N* is 5. Fragmentation of precursor ions was performed using stepped higher-energy C-trap dissociation at collision energies of 10, 20, and 40 eV. To prevent redundant sampling of abundant metabolites, a dynamic exclusion of 10 s was applied.

Untargeted LC-MS/MS data were processed using Skyline v24.1.0^82,83^ to identify and quantify select surfactin compounds. Raw spectral data were imported into Skyline, where surfactins were detected based on specified *m*/*z* ratios and corresponding fragmented ions. Retention time alignment was performed across all data files to maintain consistency in peak detection and improve comparability between samples. Processed LC-MS/MS data were then further analyzed using R v4.4.1, with surfactin log10-transformed for normalization before statistical analysis.

### Strain construction

*B. subtilis* DK1042 HK (**JMB92**) and DK1042 ME (**JMB90**) strains were generated by amplifying allele replacement constructs from genomic DNA of strains BKK34900 (168 Δ*hisB*::*kan*) and BKE13180 (168 Δ*metE*::*erm*), respectively, and transforming strain DK1042 with these gene targeting constructs through natural competence. Transformants were selected on LB agar supplemented with kanamycin and erythromycin plus lincomycin, respectively.

*B. subtilis* DK1042 HK ME (**JMB91**) was constructed by transforming strain DK1042 HK with a Δ*metE*::*erm* allele replacement construct through natural competence. Transformants were selected on LB agar supplemented with kanamycin, erythromycin, and lincomycin.

*B. subtilis* NCIB3610 ΔpBS32 (**JMB300**) was generated by transforming *B. subtilis* DK1042 with pJM598 through natural competence. Transformants were selected on LB agar plates supplemented with kanamycin and 200 ng/ml anhydrotetracycline at 30 °C. Single colonies were then streaked on LB plates and incubated at 45 °C overnight to cure pJM598. Absence of pBS32 was validated by whole-genome resequencing.

*B. subtilis* RO-NN-1 Δ*gerA*::*kan* (**JMB301**) was constructed by transforming *B. subtilis* RO-NN-1 with a DNA fragment generated by splicing by overlap extension (SOE) PCR of three PCR products: (1) 547 bp fragment upstream of *gerAA* amplified with primers oDV001/oDV002 and genomic DNA from strain RO-NN-1 as template, (2) 562 bp fragment downstream of *gerAC* amplified with primers oDV005/oDV006 and genomic DNA from strain RO-NN-1 as a template, and (3) kanamycin resistance cassette amplified with primers oDV003/oDV004 and genomic DNA from strain BKK34900^7^ as template.

Strains **JMB302-JMB306** were constructed by cotransforming *B. subtilis* DK1042 with pJM599 and a repair template generated by amplification of the *gerAA*-*gerAC* region from genomic DNA of strain RO-NN-1. Transformants were selected on LB agar plates supplemented with kanamycin and 200 ng/ml anhydrotetracycline at 30 °C. Single colonies were then streaked on LB agar plates and incubated at 45 °C overnight to cure pJM599.

Strains **JMB307-JMB309** were constructed by cotransforming *B. subtilis* DK1042 with pJM599 and a repair template generated by amplification of the *gerAA* region from genomic DNA of strain RO-NN-1. Transformants were selected on LB agar plates supplemented with kanamycin and 200 ng/ml anhydrotetracycline at 30 °C. Single colonies were then streaked on LB agar plates and incubated at 45 °C overnight to cure pJM599.

Strains **JMB310-JMB315** were constructed by cotransforming *B. subtilis* DK1042 with pJM600 and a repair template generated by amplification of the *gerAB* region from genomic DNA of strain RO-NN-1. Transformants were selected on LB agar plates supplemented with kanamycin and 200 ng/ml anhydrotetracycline at 30 °C. Single colonies were then streaked on LB agar plates and incubated at 45 °C overnight to cure pJM600.

Strains **JMB316-JMB319** were constructed by cotransforming *B. subtilis* DK1042 with pJM600 and a repair template generated by amplification of the *gerAA*-*gerAC* region from genomic DNA of strain RO-NN-1. Transformants were selected on LB agar plates supplemented with kanamycin and 200 ng/ml anhydrotetracycline at 30 °C. Single colonies were then streaked on LB agar plates and incubated at 45 °C overnight to cure pJM600.

Strain **JMB320** was constructed by transforming *B. subtilis* DK1042 with pJM602. Transformants were selected on LB agar plates supplemented with kanamycin and 200 ng/ml anhydrotetracycline at 30 °C. Single colonies were then streaked on LB agar plates and incubated at 45 °C overnight to cure pJM602.

Strains **JMB321-JMB331** were constructed by cotransforming *B. subtilis* DK1042 with pJM601 and a repair template generated by amplification of the *gerAA*-*gerAC* region from genomic DNA of strain RO-NN-1. Transformants were selected on LB agar plates supplemented with kanamycin and 200 ng/ml anhydrotetracycline at 30 °C. Single colonies were then streaked on LB agar plates and incubated at 45 °C overnight to cure pJM601.

Strain **JMB332** was constructed by cotransforming JMB308 with pJM600 and a repair template generated by amplification of the *gerAA*-*gerAC* region from genomic DNA of strain RO-NN-1. Transformants were selected on LB agar plates supplemented with kanamycin and 200 ng/ml anhydrotetracycline at 30 °C. Single colonies were then streaked on LB agar plates and incubated at 45 °C overnight to cure pJM600.

Strain **JMB333** was constructed by cotransforming JMB332 with pJM601 and a repair template generated by amplification of the *gerAC* region from genomic DNA of strain RO-NN-1. Transformants were selected on LB agar plates supplemented with kanamycin and 200 ng/ml anhydrotetracycline at 30 °C. Single colonies were then streaked on LB agar plates and incubated at 45 °C overnight to cure pJM601.

Strain **JMB334** was constructed by transforming strain JMB333 with pJM602. Transformants were selected on LB agar plates supplemented with kanamycin and 200 ng/ml anhydrotetracycline at 30 °C. Single colonies were then streaked on LB agar plates and incubated at 45 °C overnight to cure pJM602.

Strains **JMB335-JMB338** were constructed by cotransforming *B. subtilis* DK1042 with pJM603 and a repair template amplified by primers oDV029 and oDV030 from genomic DNA of strain RO-NN-1. Transformants were selected on LB agar plates supplemented with kanamycin and 200 ng/ml anhydrotetracycline at 30 °C. Single colonies were then streaked on LB agar plates and incubated at 45 °C overnight to cure pJM603.

Strains **JMB339** and **JMB340** were constructed by cotransforming strain JMB338 with pJM605 and a repair template amplified by primers oDV031 and oDV032. Transformants were selected on LB agar plates supplemented with kanamycin and 200 ng/ml anhydrotetracycline at 30 °C. Single colonies were then streaked on LB agar plates and incubated at 45 °C overnight to cure pJM605.

Strains **JMB342** and **JMB343** were constructed by cotransforming strain JMB338 with pJM604 and a repair template amplified by primers oDV031 and oDV032. Transformants were selected on LB agar plates supplemented with kanamycin and 200 ng/ml anhydrotetracycline at 30 °C. Single colonies were then streaked on LB agar plates and incubated at 45 °C overnight to cure pJM604.

Strains **JMB344–JMB352** were constructed by cotransforming *B. subtilis* DK1042 with pJM606 and a repair template amplified by primers oDV029 and oDV030 from genomic DNA of strain RO-NN-1. Transformants were selected on LB agar plates supplemented with kanamycin and 200 ng/ml anhydrotetracycline at 30 °C. Single colonies were then streaked on LB agar plates and incubated at 45 °C overnight to cure pJM606.

Strains **JMB353–JMB375** were generated by transforming *B. subtilis* P1A7, P1A11, P1B5, P1B12, P1E9, P1F11, P2B1, P2D3, P2D4, P2E11, P2G8, P2G10, P3B5, P3C6, P3D3, P3E3, P3F1, P3F10, P3G3, P3G12, P3H8, P4A9, and P4B5 with pDR244^7^, a temperature-sensitive plasmid expressing the Cre recombinase. Transformants were selected on LB agar plates supplemented with spectinomycin at 30 °C. Single colonies were then streaked on LB agar plates and LB agar plates supplemented with kanamycin (for HK strains) or erythromycin (for ME strains). Strains that grew on LB agar plates, but not on LB agar plates supplemented with antibiotics, were streaked on LB agar plates and grown overnight at 45 °C to cure pDR244. Single transformants were patched onto LB agar plates and LB agar plates supplemented with spectinomycin. Select strains that did no longer grow on spectinomycin plates were validated by whole-genome resequencing.

Strains **JMB376–JMB400** were generated by transforming strains RO-NN-1, JMB300, and JMB353–JMB375 with pJM609. Transformants were selected on LB agar plates supplemented with chloramphenicol at 37 °C.

Strain **JMB413** was generated by transforming *B. velezensis* S4 with **pIDV76** via electroporation. An overnight LB culture of S14 was diluted 100-fold into fresh NCM medium (K_2_HPO_4_ 17.4 g/l, NaCl 11.6 g/l, glucose 5 g/l, tryptone 5 g/l, yeast extract 1 g/l, sodium citrate 0.3 g/l, MgSO_4_.7H_2_O 0.05 g/l, and sorbitol 91.1 g/l, pH 7.2). When the culture reached an OD_600_ of 0.8, the cell-wall weakening agent glycine/DL-threonine was added to a final concentration of 1% (w/v), followed by further incubation for an additional 40 min at 37 °C. Cells were harvested by centrifugation (7000 × *g*, 10 min, room temperature) and washed 5 times with TSMM buffer (0.5 M trehalose, 0.5 M sorbitol, 0.5 M mannitol, and 10% glycerol). The cell pellets were then resuspended into 1/100 of the original culture volume. Aliquots (100 µl) were either used immediately for transformation or stored at −80 °C. For electroporation, electrocompetent cells were mixed with 200 ng plasmid DNA and transferred to a 1-mm gap electroporation cuvette. Cells were subjected to a single pulse at 2.1 kV cm^−^¹. Immediately following the pulse, cells were rescued in 1 ml of mRM medium (1× LB, 0.38 M mannitol, 0.5 M sorbitol, 1.05 mM nitrilotriacetic acid, 0.59 mM MgSO_4_·7H_2_O, 0.91 mM CaCl_2_·2H_2_O, 40 µM FeSO_4_·7H_2_O) and incubated at 37 °C for 6 h. Recovered cells were then plated on mLB agar (1× LB, 1.05 mM nitrilotriacetic acid, 0.59 mM MgSO_4_·7H_2_O, 0.91 mM CaCl_2_·2H_2_O, 40 µM FeSO_4_·7H_2_O) supplemented with chloramphenicol for selection.

Strain **JMB414** (*B. velezensis* S4 Δ*hisB*::*kan*) was generated by transforming JMB413 with a linear *hisB*::*kan* construct via natural competence. The allele replacement construct was synthesized by Twist Bioscience (San Francisco, CA, USA) and amplified using primers oAM001 and oAM002. Genetic competence in JMB413 was induced as described previously^84^. An overnight culture of JMB413 was reinoculated into fresh LB containing chloramphenicol at an initial OD_600_ of 0.1. Cells were grown at 37 °C to an OD_600_ of 0.5, at which point xylose was added to a final concentration of 1% (w/v), followed by incubation for another 1 h. Ten microliters of the linear *hisB*::*kan* construct were added to 200 µl competent cells, and the mixture was incubated at 37 °C for 90 min. Cells were then plated on LB plates containing kanamycin to select for transformants. To cure the pIDV76 plasmid, transformants were grown overnight in LB containing kanamycin, then diluted in LB and plated on LB agar plates containing kanamycin.

Strain **JMB415** (*B. velezensis* S4 Δ*metE*::*erm*) was generated by transforming JMB413 with a linear *metE*::*erm* construct via natural competence. The allele replacement construct was synthesized by Twist Bioscience (San Francisco, CA, USA) and amplified using primers oAM003 and oAM004. Genetic competence in JMB413 was induced as described above.

Strain **JMB416** (*B. velezensis* S4 Δ*metE*::*erm* Δ*hisB*::*kan*) was constructed by genome shuffling using strains JMB414 and JMB415, as described above.

Strain **JMC1** (*C. thermocellum* DSM1313 Δ*trpC*::*cat*) was generated by allele exchange mutagenesis using plasmid pDGO145^85^. DSM 1313 was transformed with **pJM611**, which carries a thiamphenicol resistance gene flanked by 700 bp regions homologous to the upstream and downstream sequences of *trpC*, via electroporation as previously described^46^. Strain DSM1313 was grown in 100 ml CTFUD to an OD_600_ ~1.0 under anaerobic conditions. Cells were then harvested by centrifugation (6000 × *g*, 10 min, room temperature), washed three times with Wash buffer (250 mM sucrose, 10% glycerol) and resuspended in 200 μl wash buffer. Plasmid DNA (400 ng) was then added to 20 μl electrocompetent cells in a 1 mm electroporation cuvette, and a square electrical pulse (1.5 kV, 1.5 ms) was applied. Pulsed cells were resuspended in 3 ml CTFUD and incubated overnight at 50 °C. Single crossovers were selected on CTFUD agar supplemented with thiamphenicol at 50 °C. Individual colonies were then grown in CTFUD and plated on CTFUD agar supplemented with 10 µg/ml 5-fluoro-2′-deoxyuradine (FUDR) for counter-selection, which selects for the loss of a functional *tdk* gene encoding thymidine kinase carried on the plasmid. Double crossovers were verified by whole-genome resequencing.

Strain **JMC2** (*C. thermocellum* DSM1313 Δ*trpC*::*cat* Δ*pyrF*::*neo*) was generated by transforming strain JMC1 with **pJM612** by electroporation as described above^46^. Individual colonies were then grown in CTFUD and plated on CTFUD agar supplemented with 10 µg/ml 5-fluoro-2′-deoxyuradine (FUDR) for counter-selection, which selects for the loss of a functional *tdk* gene carried on the plasmid. Loss of a functional *pyrF* gene was validated by growing the strains in CTFUD-NY medium supplemented with neomycin, 40 µg/ml uracil, and 500 µg/ml 5-fluoroorotic acid. Strain JMC2 was verified by whole-genome resequencing.

Strain **JMN150** (*N. aromaticivorans* F199 Δ*hisB*::*kan*) was generated by allele exchange mutagenesis using the suicide vector plasmid pAK405, as previously described^86,87^. Plasmid **pJM613**, which carries a kanamycin resistance cassette derived from the pAK405 backbone flanked by homologous regions (500–700 bp) upstream and downstream of *hisB*, was mobilized into F199 via biparental conjugation using *E. coli* WM6026, a diaminopimelic acid (DAP) auxotroph, as described previously^86^. Strains F199 and WM6026 carrying pJM613 were grown in LB containing DAP and kanamycin, as appropriate. Strain F199 was then diluted 1:100 into 50 ml fresh LB and grown to mid-log phase. Cultures were pelleted by centrifugation (6000 × *g*, 10 min, room temperature), mixed at a recipient-to-donor ratio of 5:1, and plated on sterile filter paper placed on LB agar containing DAP. Plates were incubated overnight at 30 °C. The filter paper was then transferred to 5 ml of fresh LB and vortexed to resuspend the cells. Cells were pelleted, resuspended in 1 ml fresh LB, and plated on R2A agar supplemented with kanamycin at 30 °C to isolate transconjugants. The selection was performed in the absence of DAP to counterselect against the WM6026 donor strain. Exconjugants were clonally purified by restreaking to the same selection media. Exconjugants were then grown overnight in R2A broth without antibiotics and plated to R2A with streptomycin supplemented with 2.5 g/L NaCl to counterselect against the integrated plasmid. Streptomycin-resistant colonies were screened by patching for histidine auxotrophy on 457 media with 0.2 g/L glucose. Gene replacement was further verified with colony PCR and whole-genome resequencing.

Strain **JMN154** (*N. aromaticivorans* F199 Δ*hisB*::*kan* Δ*leuB*::*cat*) was generated by introducing plasmid **pJM614** into strain JMN150 as described above.

Strain **JMS1** (*S. stutzeri* A1501 Δ*hisB*::*kan*) was generated by transforming strain A1501 with a linear *hisB*::*kan* construct via natural competence. Strain A1501 was grown overnight in LB at 37 °C. A 500 μl aliquot of the overnight culture was then inoculated in 25 ml of fresh Competence medium (LB, 10 mM Tris-HCl, pH 7.5, 0.2% glucose, 1 mM MgSO_4_) and grown to early stationary phase. Cells from 5 ml culture were harvested by centrifugation (7000 × *g*, 10 min, room temperature). The pellet was then resuspended in 200 μl of LB and mixed with 500 ng *hisB*::*kan* construct. The cell suspension was spotted onto a LB agar plate and incubated for 24 h at 37 °C. Cells were scraped off the plate, resuspended in LB and plated on LB agar supplemented with kanamycin.

Strain **JMS2** (*S. stutzeri* A1501 *trpC*::*apr hisB*::*kan*) was generated by transforming strain JMS1 with a linear *trpC*::*apr* construct via natural competence, as described above. The resulting strain was verified by whole-genome resequencing.

### Reporting summary

Further information on research design is available in the Nature Portfolio Reporting Summary linked to this article.

## Supplementary information

Supplementary Information

Peer Review File

Description of Additional Supplementary Files

Supplementary Data 1

Supplementary Data 2

Supplementary Data 3

Supplementary Data 4

Reporting Summary

## Source data

Source Data

## Data Availability

The genome sequencing data generated in this study for the *B. subtilis* mapping population have been deposited in the NCBI BioProject database under accession code PRJNA1330897. The genome sequencing data generated in this study for the *B. velezensis*, *C. thermocellum*, *N. aromaticivorans*, and *S. stutzeri* shuffled strains, as well as recombinant *B. subtilis* strains generated via triparental shuffling, have been deposited in the NCBI BioProject database under accession code PRJNA1451957. The Proteomics data generated in this study have been deposited in the ProteomeXchange Consortium via the MASSIVE repository (MSV000101244) under accession code PXD076164 [https://massive.ucsd.edu/ProteoSAFe/dataset.jsp?task=25bcd3fd3793438c8fdef17d84d0efbb]. The sequencing data of *B. subtilis* shuffled strains derived from strains RO-NN-1 and 168, used in this study, are available in the NCBI BioProject database under accession code PRJNA669142. Other data generated in this study are included in the Supplementary Materials. Source data are provided with this paper.

## Code availability

The source code used to generate a recombination map of the *B. subtilis* QTL population is available at https://github.com/LaneMatthewJ/heritability_illustrator/ and has been deposited in the Zenodo repository at 10.5281/zenodo.19687706. The source code used for genetic mapping is available at https://github.com/harichhetri/Bsubtilis_qtl_mapping and has been deposited in the Zenodo repository at 10.5281/zenodo.19654579.

## Materials availability

Strains and plasmids generated in this study will be provided on request, subject to a material transfer agreement.
