# Supplementary material for: Genome shuffling enables quantitative trait locus mapping in Bacillus subtilis
Source: Nat Commun. 2026 Aug 5;17:7852. doi: 10.1038/s41467-026-72929-0 (PMC13443641; doi:10.1038/s41467-026-72929-0)
Supplement: Supplementary file 1 [file 41467_2026_72929_MOESM1_ESM.pdf]

Supplementary Information for

Genome shuffling enables quantitative trait locus mapping in *Bacillus subtilis*

Delyana P. Vasileva[1,2], Hari B. Chhetri[1,2], Leah H. Hochanadel[1], Jared C. Streich[1,2], John H. Lagergren[1,2], Matthew J. Lane[1,3], Apurv Mhatre[1], Oumar Sacko[1], Marco N Allemann[1], Edanur Oksuz[1,3], Dana L. Carper[1,2], Benjamin Rubino[1,3], Sadie Chitwood[1], Zachary D. Schmitz[1], Dawn M. Klingeman[1,2], Paul E. Abraham[1], Richard J. Giannone[1,2], Daniel A. Jacobson[1,2*], and Joshua K. Michener[1,2*]

[1]Biosciences Division, Oak Ridge National Laboratory; Oak Ridge, TN, 37830, USA.
[2]Center for Bioenergy Innovation, Oak Ridge National Laboratory; Oak Ridge, TN, 37830, USA.
[3]Bredesen Center for Interdisciplinary Research and Graduate Education, University of Tennessee, Knoxville; Knoxville, TN, 37996, USA.

*: Corresponding authors: jacobsonda@ornl.gov, michenerjk@ornl.gov

The PDF file includes:

Supplementary Figs. 1 to 29
Supplementary Tables 1 to 3
References

Other Supplementary Materials for this manuscript include the following:

Supplementary Data 1 to 4

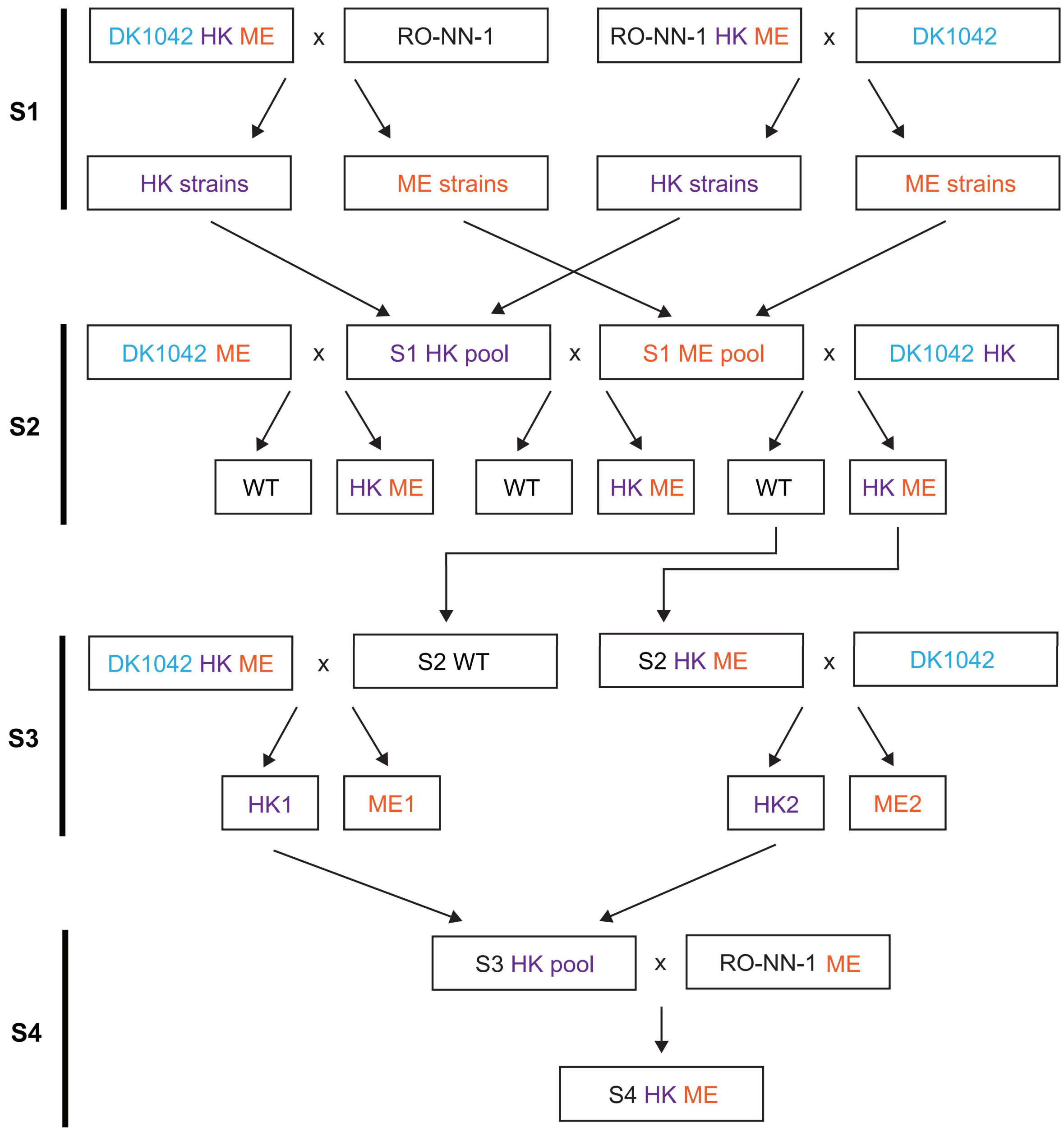

Supplementary Fig. 1: Schematic overview of the construction of the *B. subtilis* QTL mapping population. The mapping population of recombinant strains was generated through three and four rounds of genome shuffling via protoplast fusion, using *B. subtilis* RO-NN-1 and DK1042 as parental strains, as described in the Methods section. S1, S2, S3, and S4 indicate the shuffling rounds. WT: wild type; HK: Δ*hisB*::*kan*; ME: Δ*metE*::*erm*.

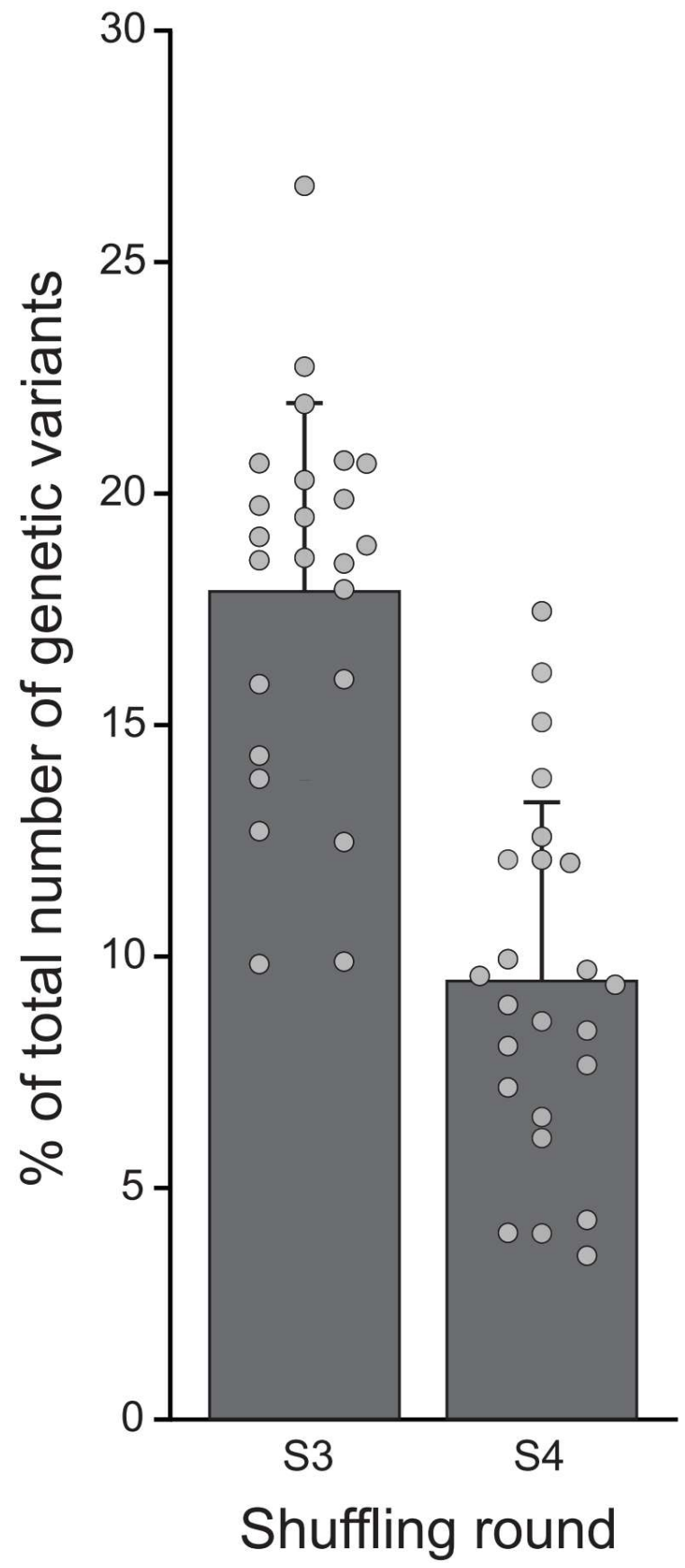

Supplementary Fig. 2: Genome-wide recombination in the *B. subtilis* QTL population. The fraction of the genome that was recombined after the S3 and S4 shuffling rounds between strains RO-NN-1 and DK1042 (Supplementary Fig. 1) is shown. n = 24 recombinant strains. Data are presented as mean ± SD. Source data are provided as a Source Data file.

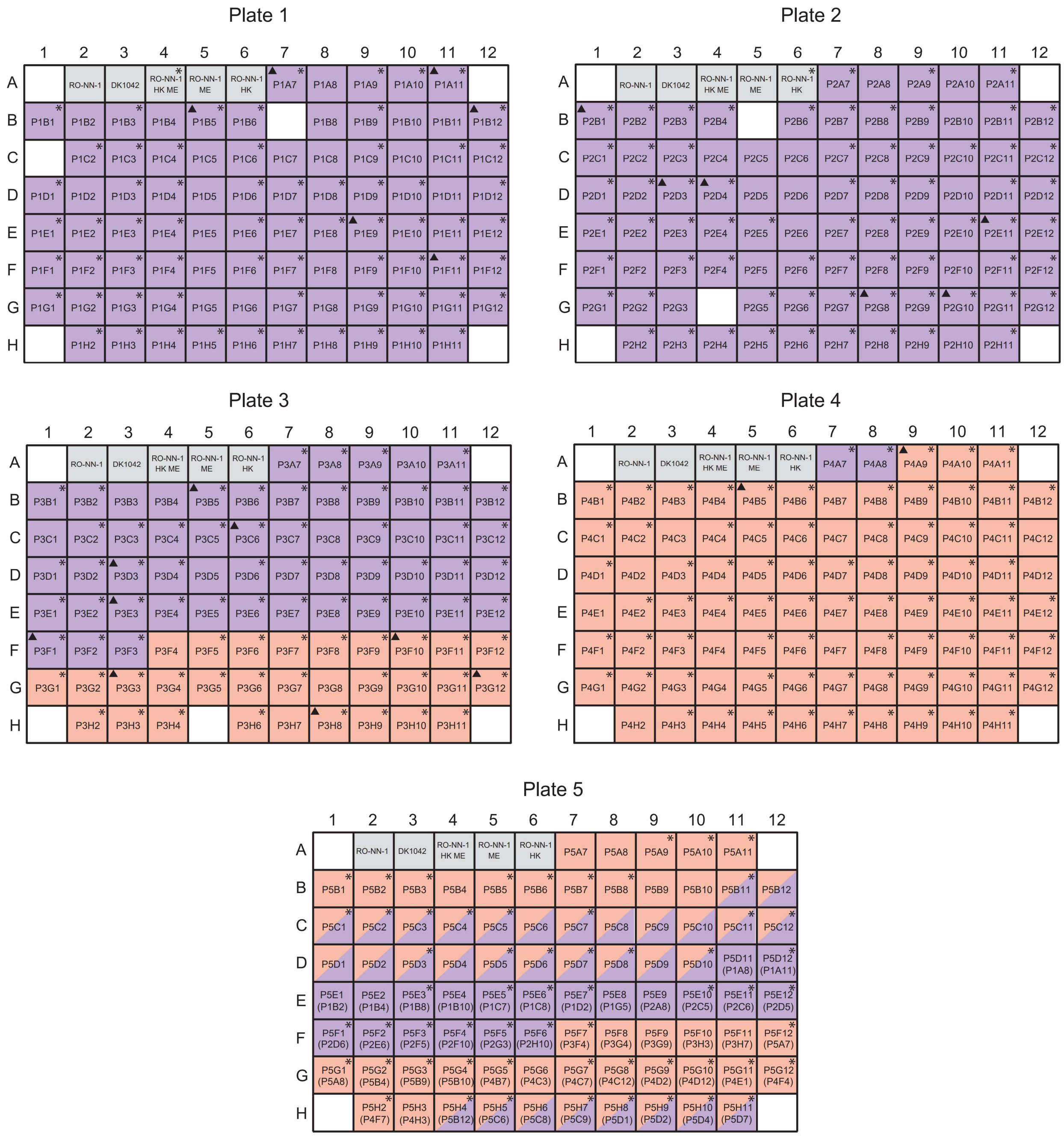

Supplementary Fig. 3: Layout of the 96-well plates with *B. subtilis* shuffled strains generated in this study. Wells containing HK and ME shuffled strains are highlighted in purple and orange, respectively. Wells containing HK ME double-resistant shuffled strains are highlighted using both purple and orange. Blank wells are empty. *B. subtilis* RO-NN-1, DK1042, RO-NN-1 HK ME, RO-NN-1 HK and RO-NN-1 ME are included on each plate, with their corresponding wells highlighted in gray. Plate 5 includes randomly selected replicates, indicated in parentheses. The 350 genetically diverse strains used for genetic mapping are marked with asterisks. The 23 most genetically diverse shuffled strains used for the phenotypic assays in Fig. 4 and Supplementary Figs. 21-26 are marked with triangles. HK: Δ*hisB*::*kan*; ME: Δ*metE*::*erm.*

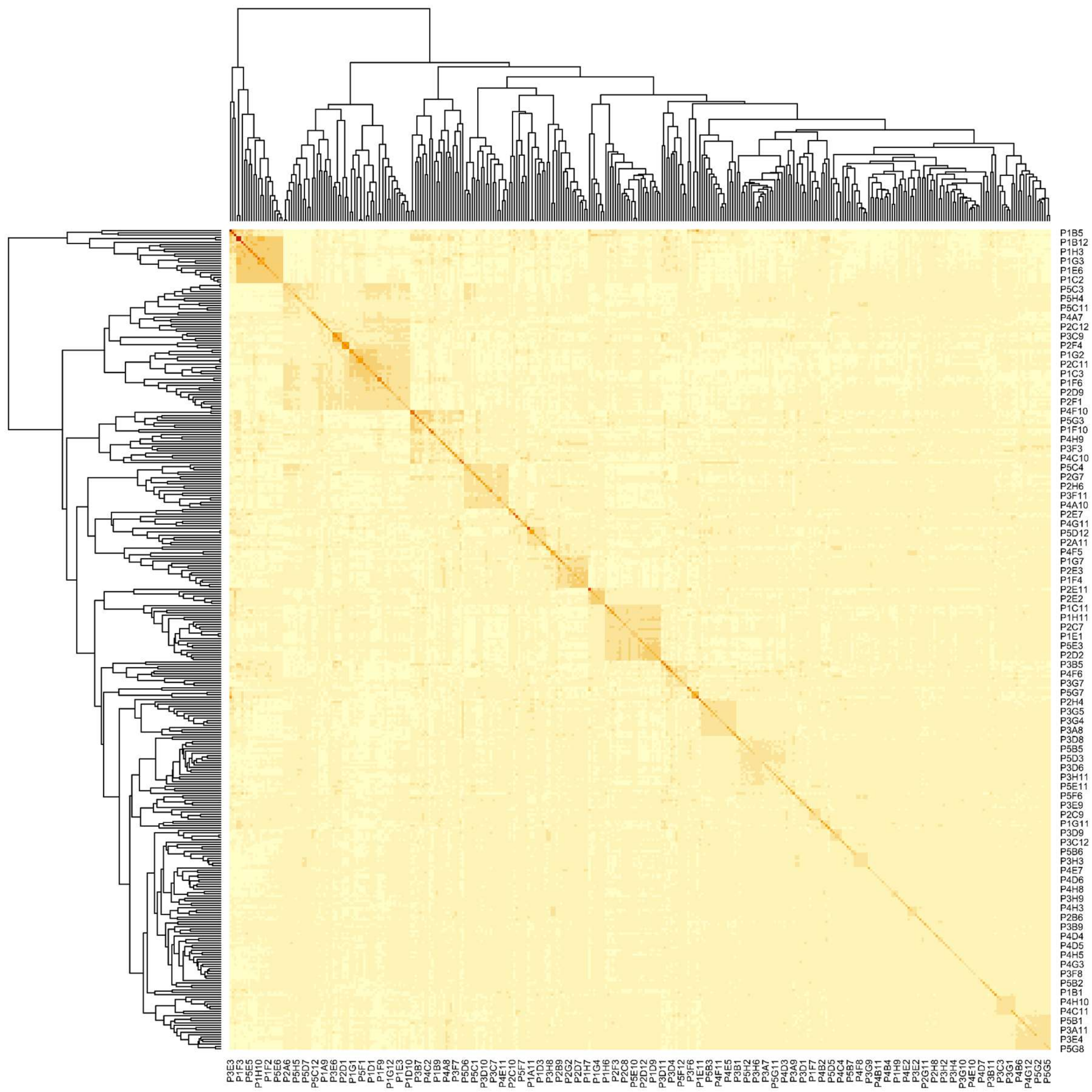

Supplementary Fig. 4: Heatmap and dendrogram of a genetic relationship matrix of the *B. subtilis* QTL population. The kinship matrix was estimated using the Zhang algorithm [1] based on 59,571 SNPs at a minor allele frequency threshold of 0.03 across 350 shuffled strains.

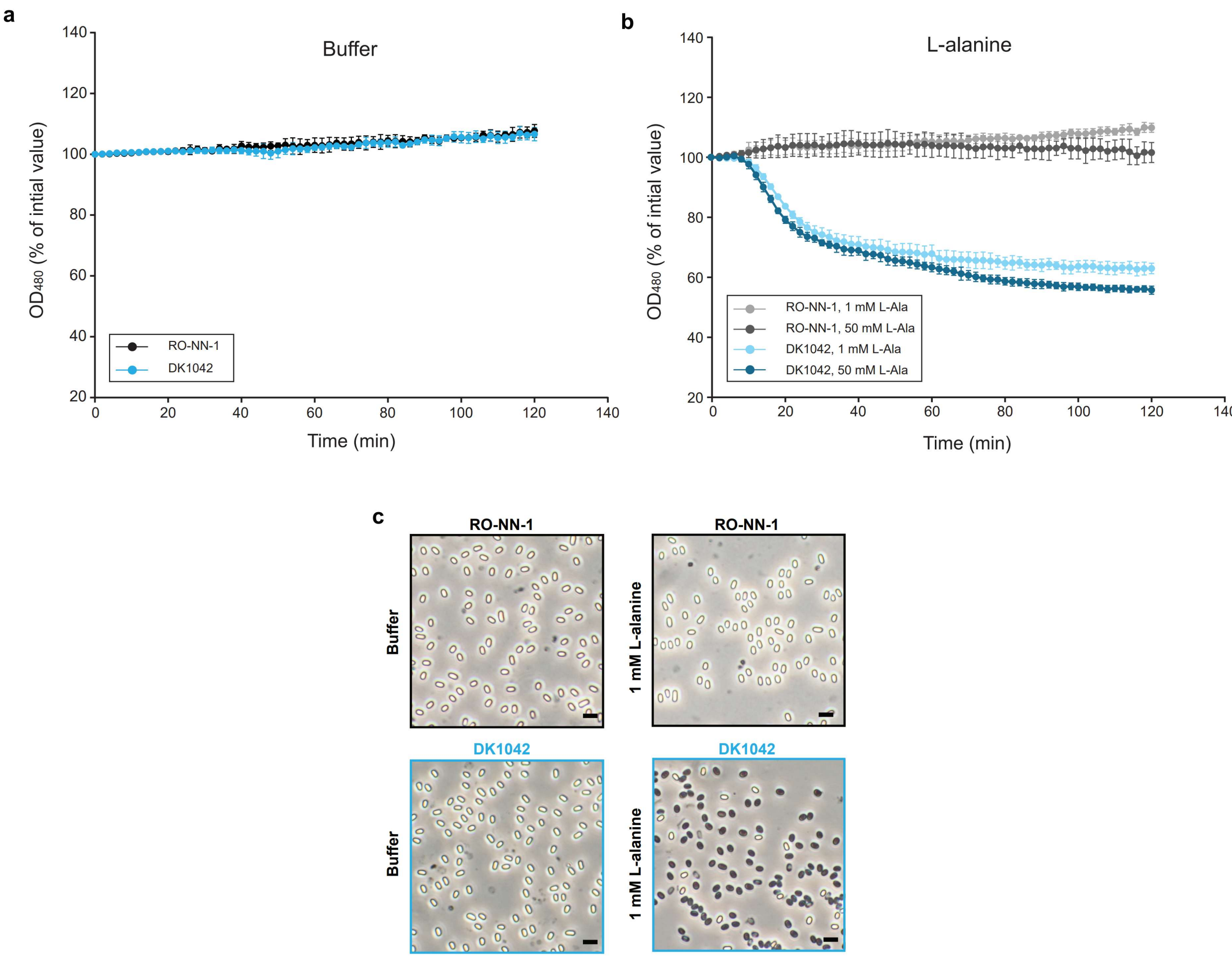

Supplementary Fig. 5: Spore germination of *B. subtilis* RO-NN-1 and DK1042 in response to L-alanine. Spores of strains RO-NN-1 and DK1042 were incubated with (**a**) buffer and (**b**) 1 mM or 50 mM L-alanine, and spore germination was assessed as percent reduction in OD_{480}. (**c**) Representative phase-contrast images of RO-NN-1 and DK1042 spores after 120 min incubation with buffer and 1 mM L-alanine. *n* = 4 independent experiments. Data represent the mean ± SD. Scale bar, 2 µm. Source data are provided as a Source Data file.

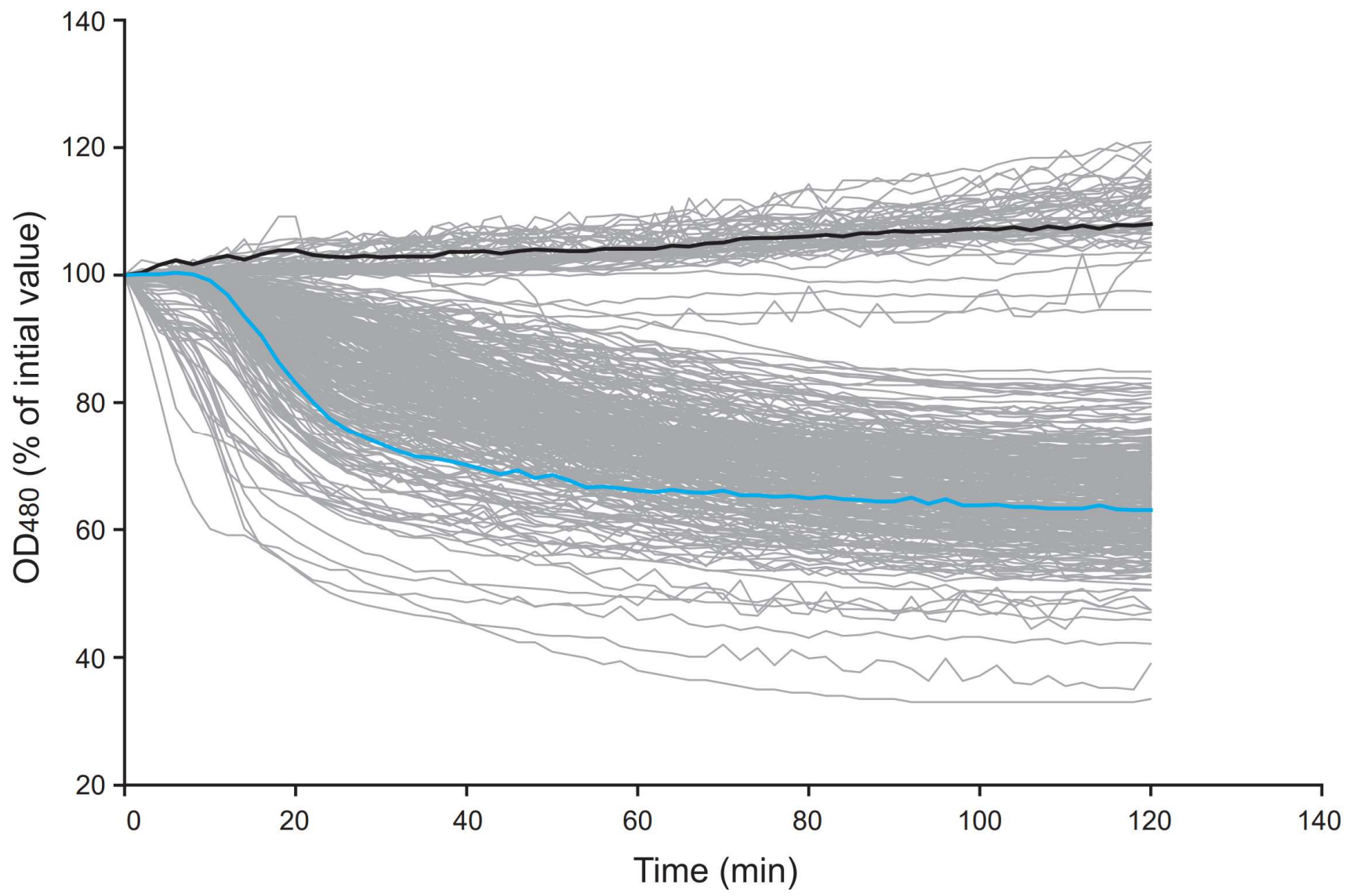

Supplementary Fig. 6: Spore germination of the *B. subtilis* QTL population in response to 1 mM L-alanine. Spores of the 350 shuffled strains and the QTL parents were incubated with 1 mM L-alanine, and spore germination was assessed as percent reduction in initial OD_{480} over the course of 120 min. Gray, black and blue lines indicate data from the 350 shuffled strains, RO-NN-1 and DK1042, respectively. *n* = 1 experiment. Source data are provided as a Source Data file.

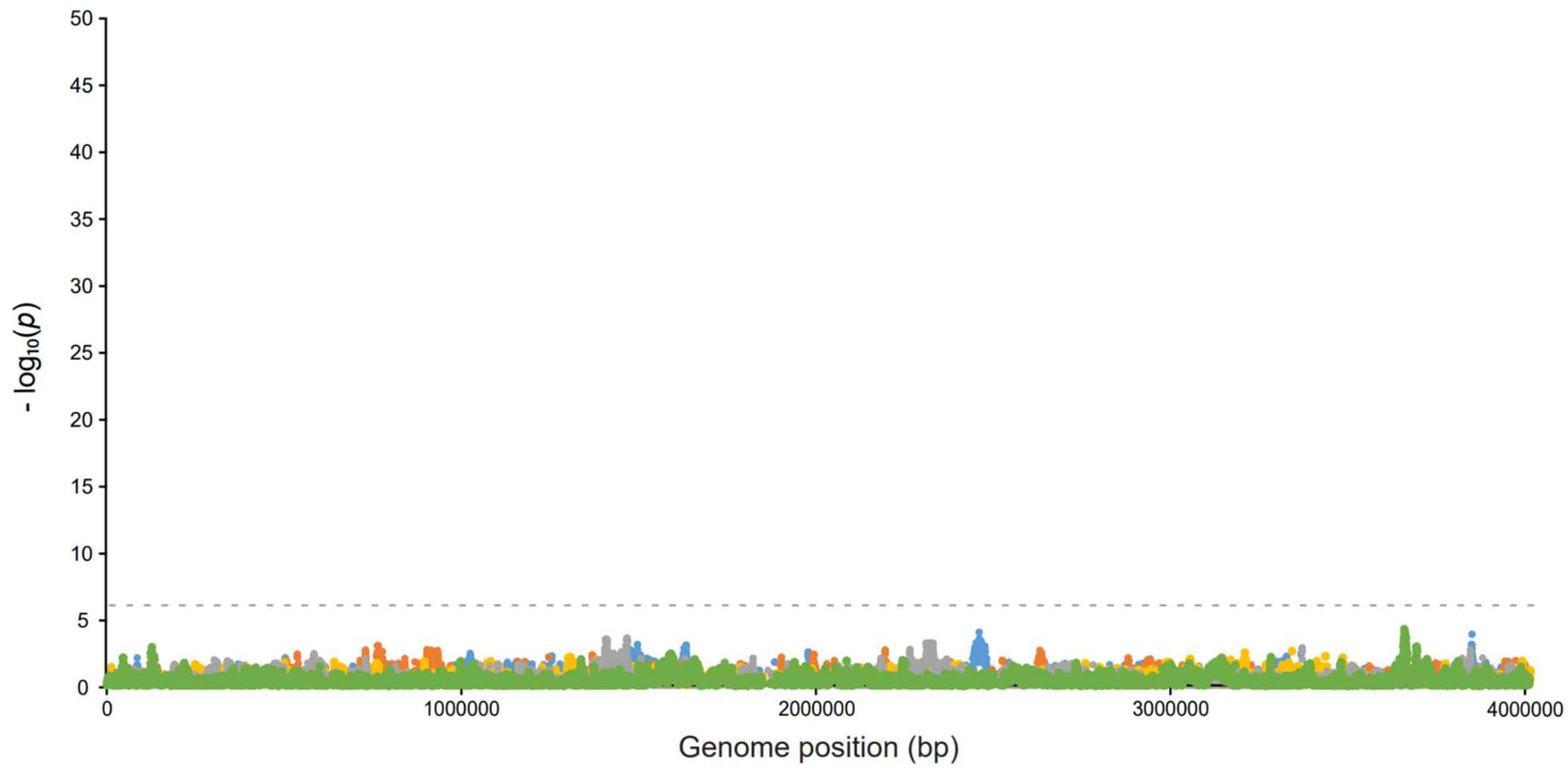

Supplementary Fig. 7: Manhattan plot using randomized spore germination phenotypic data demonstrates no significant association in the *B. subtilis* QTL population. Intrinsic noise from genetic variation was estimated by running QTL mapping permutations with real genotypic data and randomized binary phenotypes. The colors indicate association results from different permutations. The gray dashed line represents the Bonferroni-corrected significance threshold ($p \leq 0.05$).

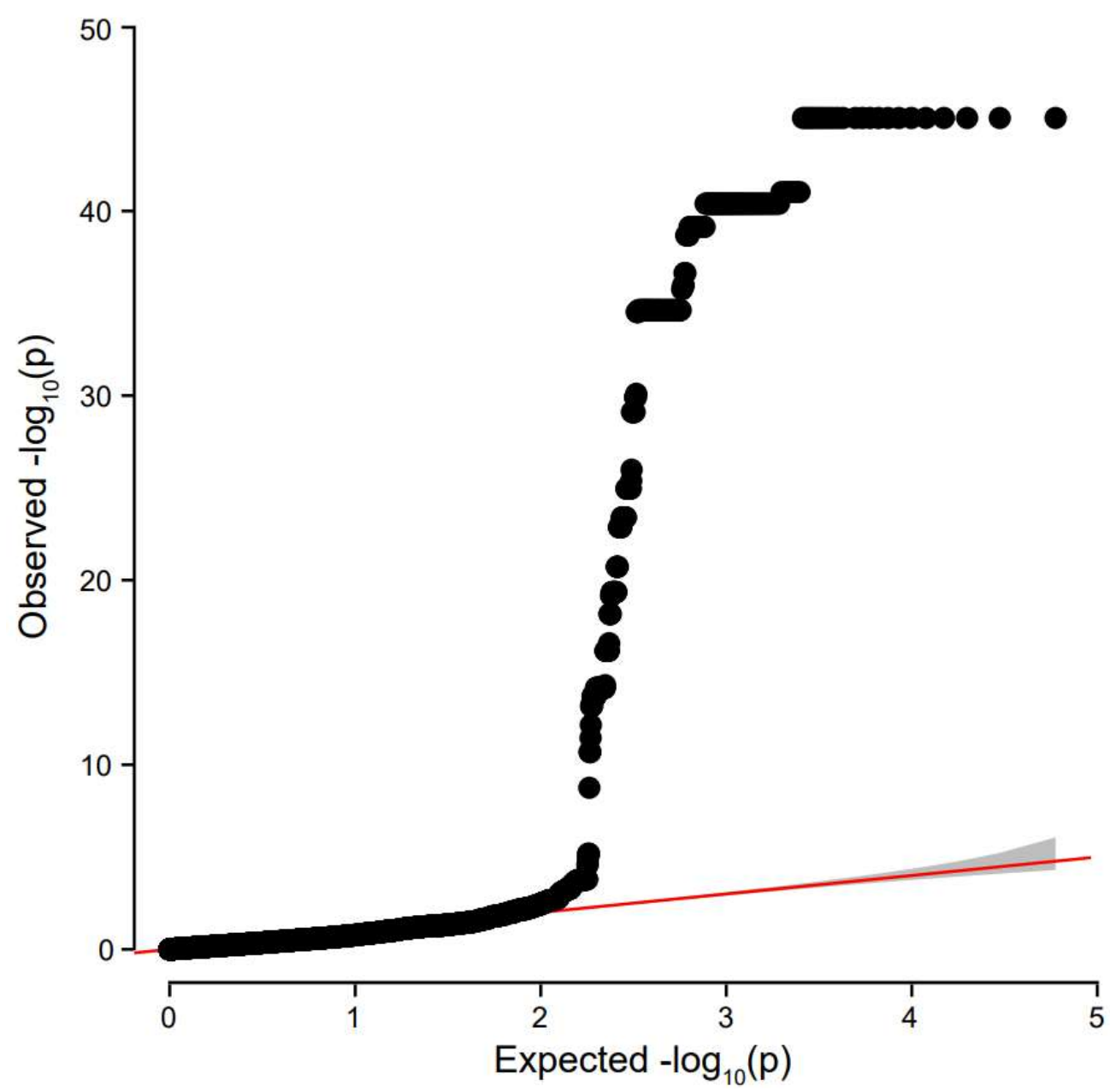

Supplementary Fig. 8: Q-Q plot corresponding to the spore germination Manhattan plot in Fig. 2c.

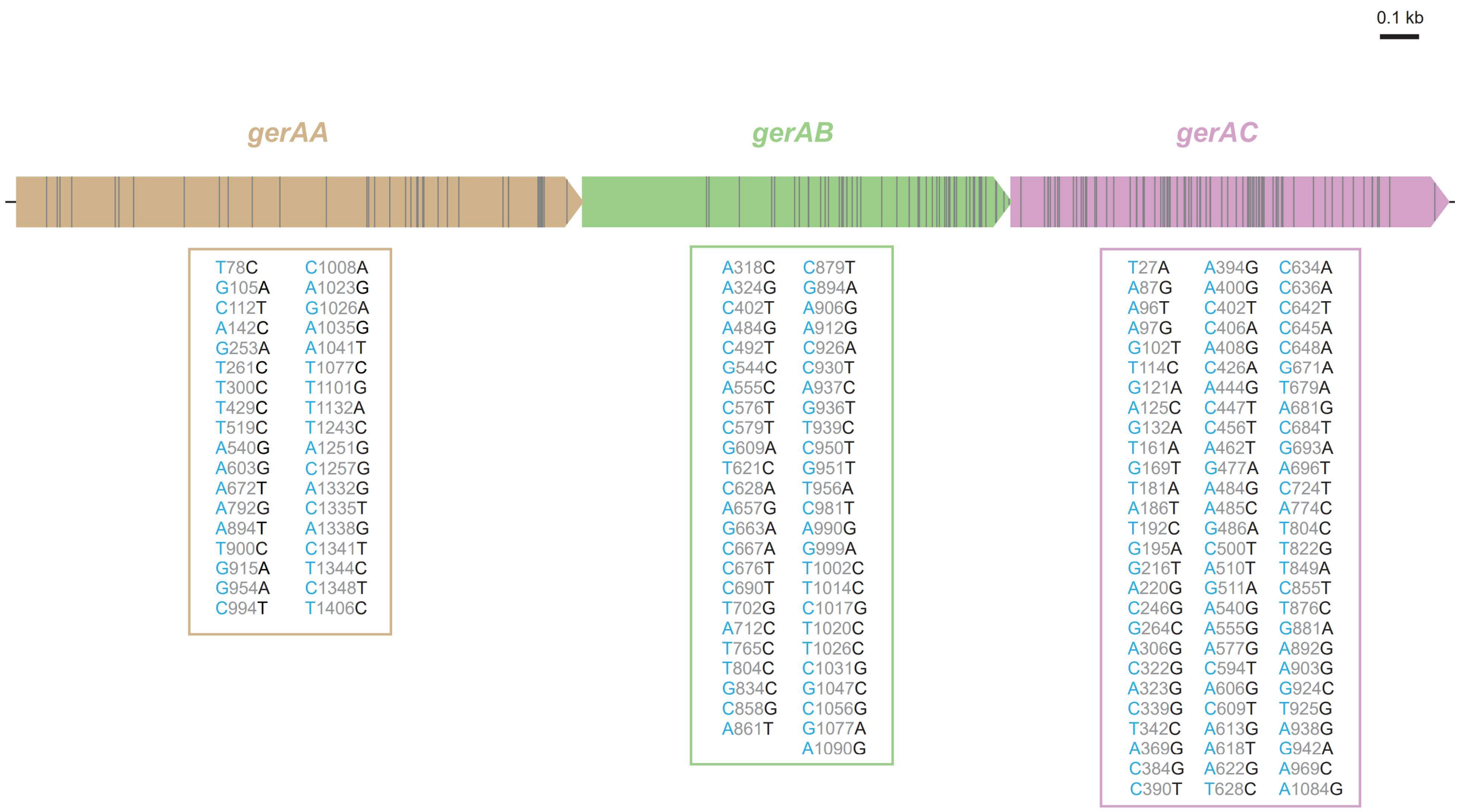

Supplementary Fig. 9: Overview of genetic variants within the *gerAA*-*gerAC* locus in *B. subtilis* DK1042 and RO-NN-1. Locations of the SNPs in *gerAA* (tan), *gerAB* (green), and *gerAC* (pink) are highlighted in gray. Blue and black letters indicate genetic variants in DK1042 and RO-NN-1, respectively.

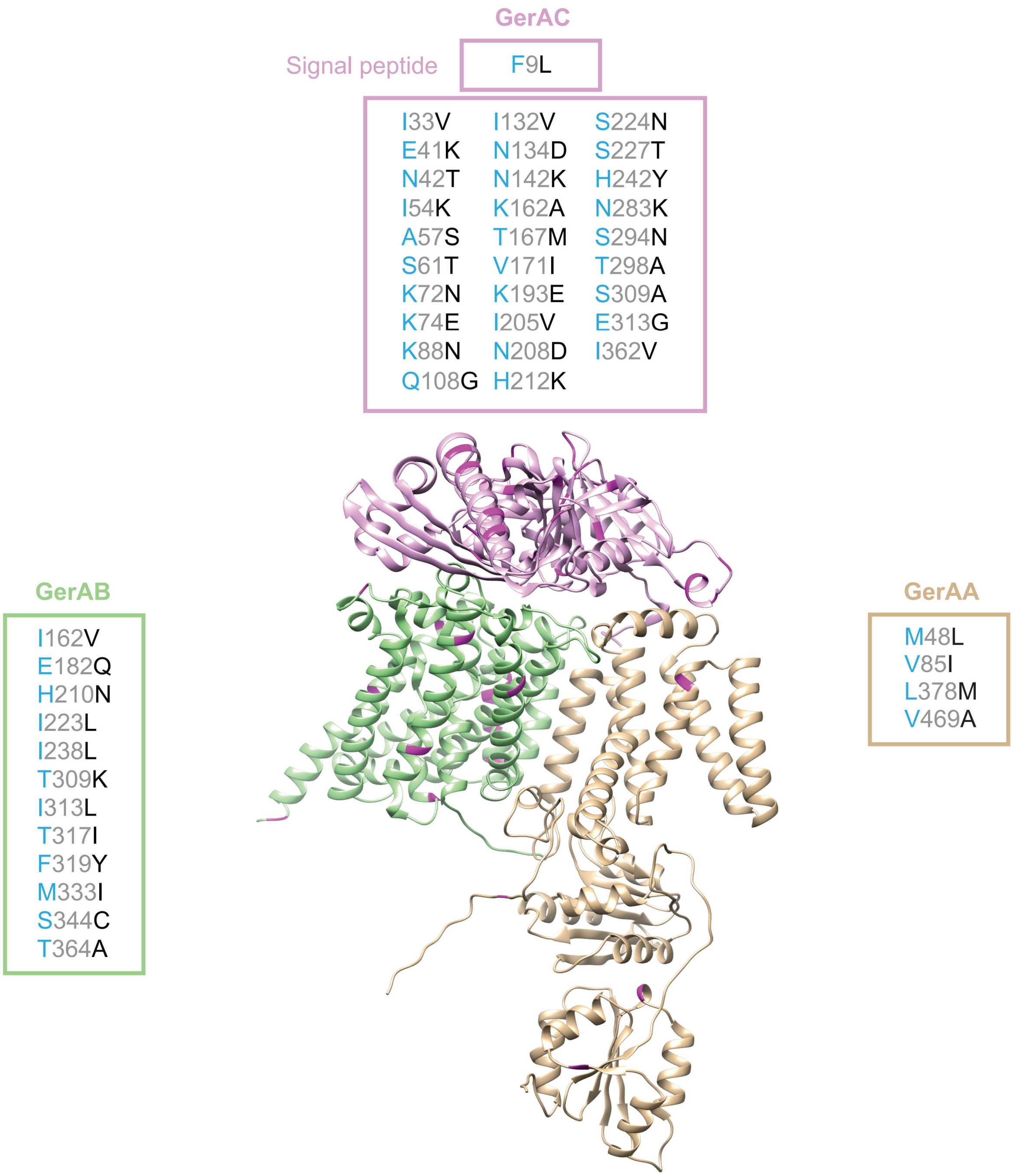

Supplementary Fig. 10: Overview of amino acid differences in the GerAA, GerAB, and GerAC proteins between *B. subtilis* RO-NN-1 and DK1042. The AlphaFold2 model of the GerAA (tan), GerAB (green), and GerAC (light pink) trimer was obtained from [2]. Amino acids that differ between strain DK1042 and RO-NN-1 are highlighted in dark pink. Blue and black letters indicate amino acid variants in DK1042 and RO-NN-1, respectively.

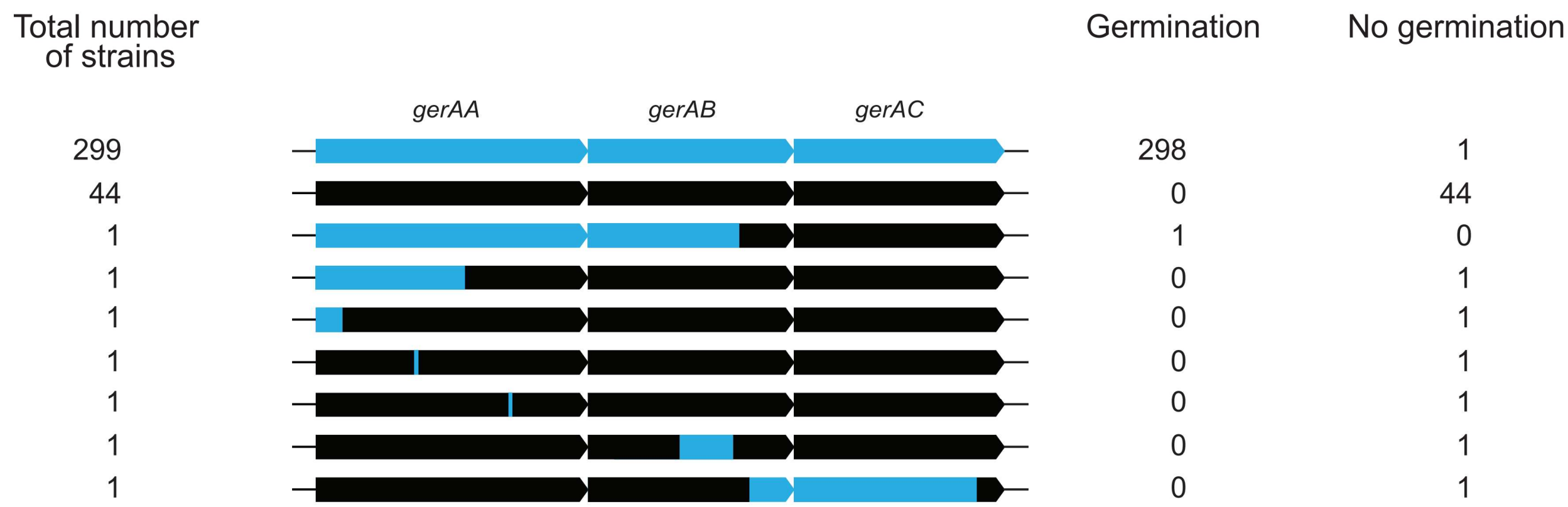

Supplementary Fig. 11: Distribution of candidate causative spore germination loci in the *B. subtilis* QTL mapping population. Blue and black regions indicate sequences originating from *B. subtilis* DK1042 and RO-NN-1, respectively. The number of strains from each genotype and their respective phenotypes are shown.

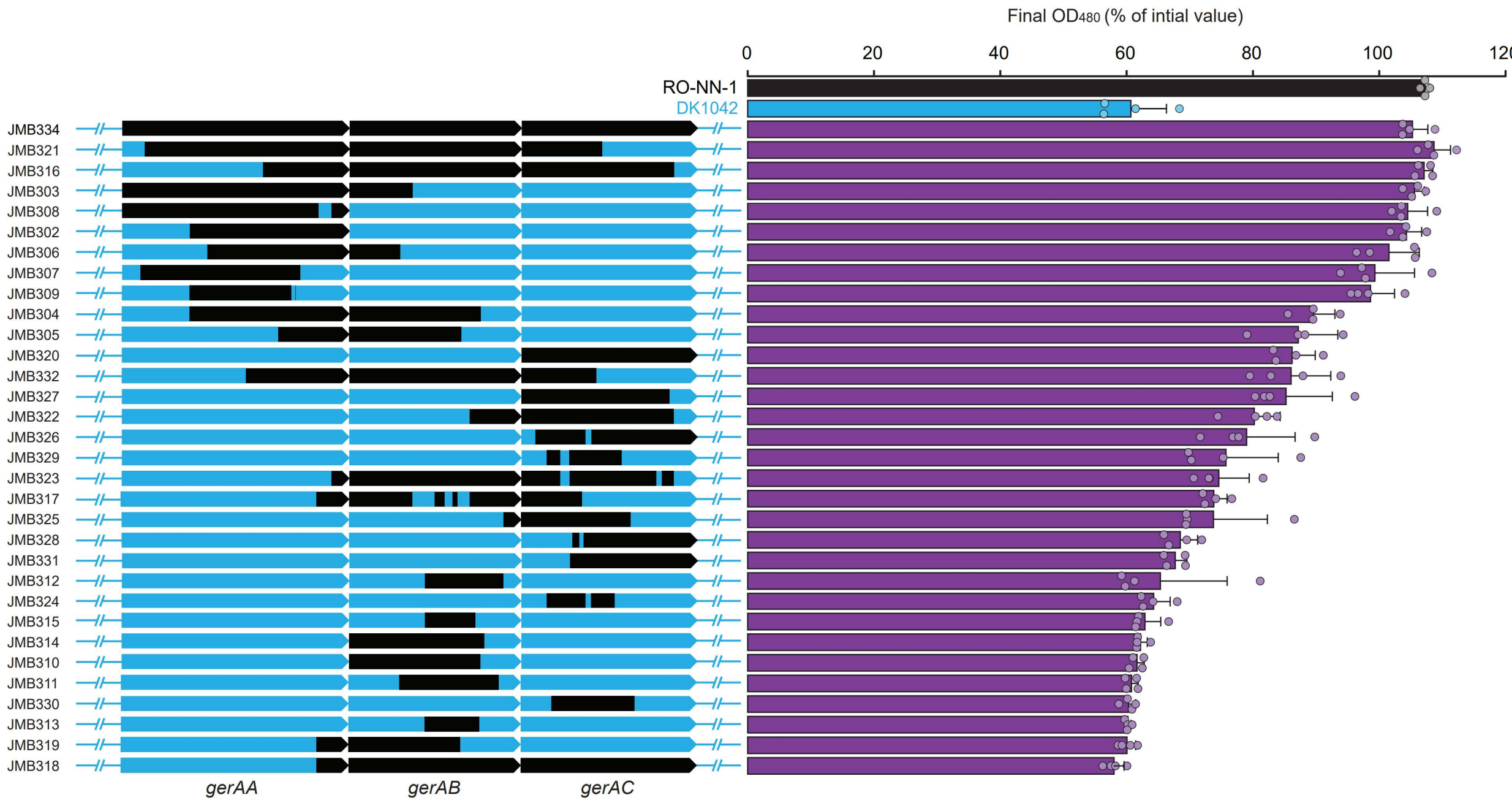

Supplementary Fig. 12: Genetic swaps validate causal genetic loci for spore germination in response to L-alanine. Gene segments within the *gerA* operon in strain DK1042 were replaced with corresponding segments from strain RO-NN-1 using a CRISPR-based approach and their spore germination abilities were evaluated. The bar plot shows optical densities measured after incubation of spores of each strain with 1 mM L-alanine for 120 min. Data are presented as a percentage of the initial OD_{480} of the phase-bright spores. $n = 4$ independent experiments. Values are presented as mean ± SD. Blue and black regions indicate sequences originating from *B. subtilis* DK1042 and RO-NN-1, respectively. Source data are provided as a Source Data file.

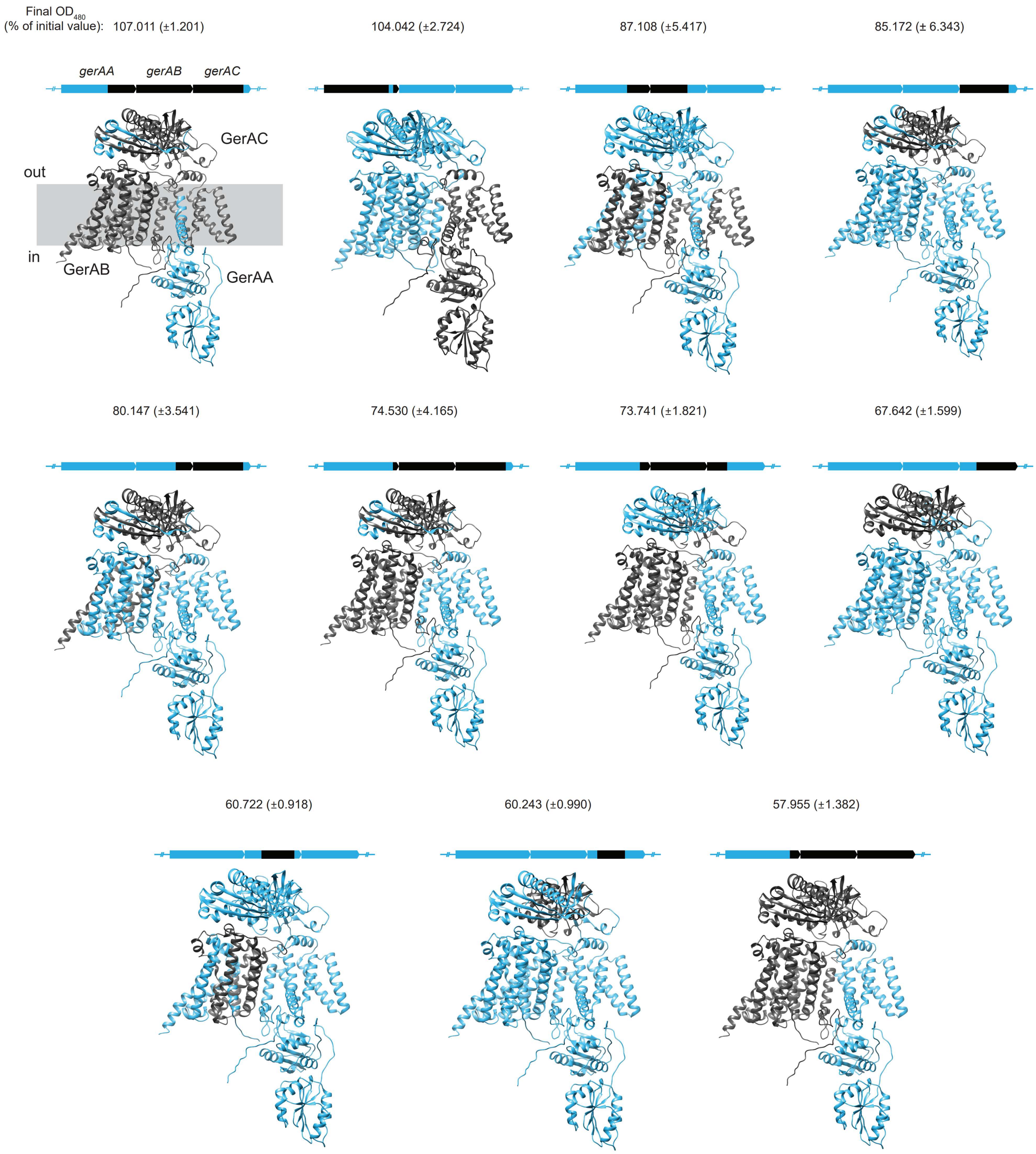

Supplementary Fig. 13: Structural models of the GerAA-GerAB-GerAC trimers resulting from genetic swaps in the *gerA* locus shown in Fig. 2D. Final OD_{480} values presented as a percentage of the initial OD_{480} of the phase-bright spores are indicated. n = 3 independent experiments. Data are presented as mean ± SD. The AlphaFold2 model of the GerAA-GerAB-GerAC trimer was obtained from [2]. Topology is based on protease accessibility studies of GerAA and GerAC [3,4]. Blue and black regions indicate sequences originating from *B. subtilis* DK1042 and RO-NN-1, respectively.

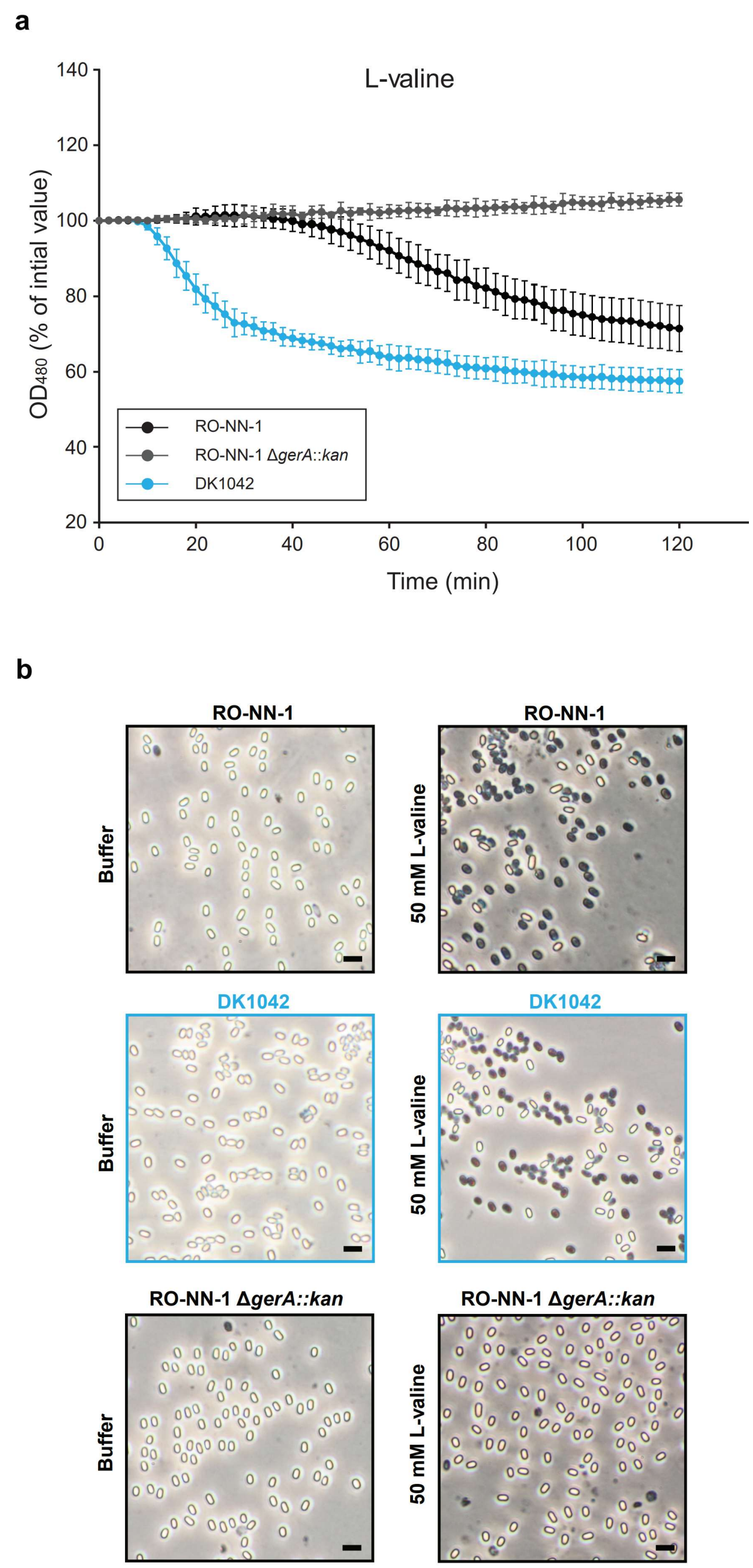

Supplementary Fig. 14: Spores of *B. subtilis* RO-NN-1 and DK1042 germinate in response to L-valine. (**a**) Spores of strains RO-NN-1, RO-NN-1 Δ*gerA*::*kan* and DK1042 were incubated with 50 mM L-valine, and spore germination was assessed as percent reduction in OD_{480}. (**b**) Representative phase-contrast images of RO-NN-1, RO-NN-1 Δ*gerA*::*kan* and DK1042 spores after 120 min incubation with buffer and 50 mM L-valine. *n* = 4 independent experiments. Values are presented as mean ± SD. Scale bar, 2 µm. Source data are provided as a Source Data file.

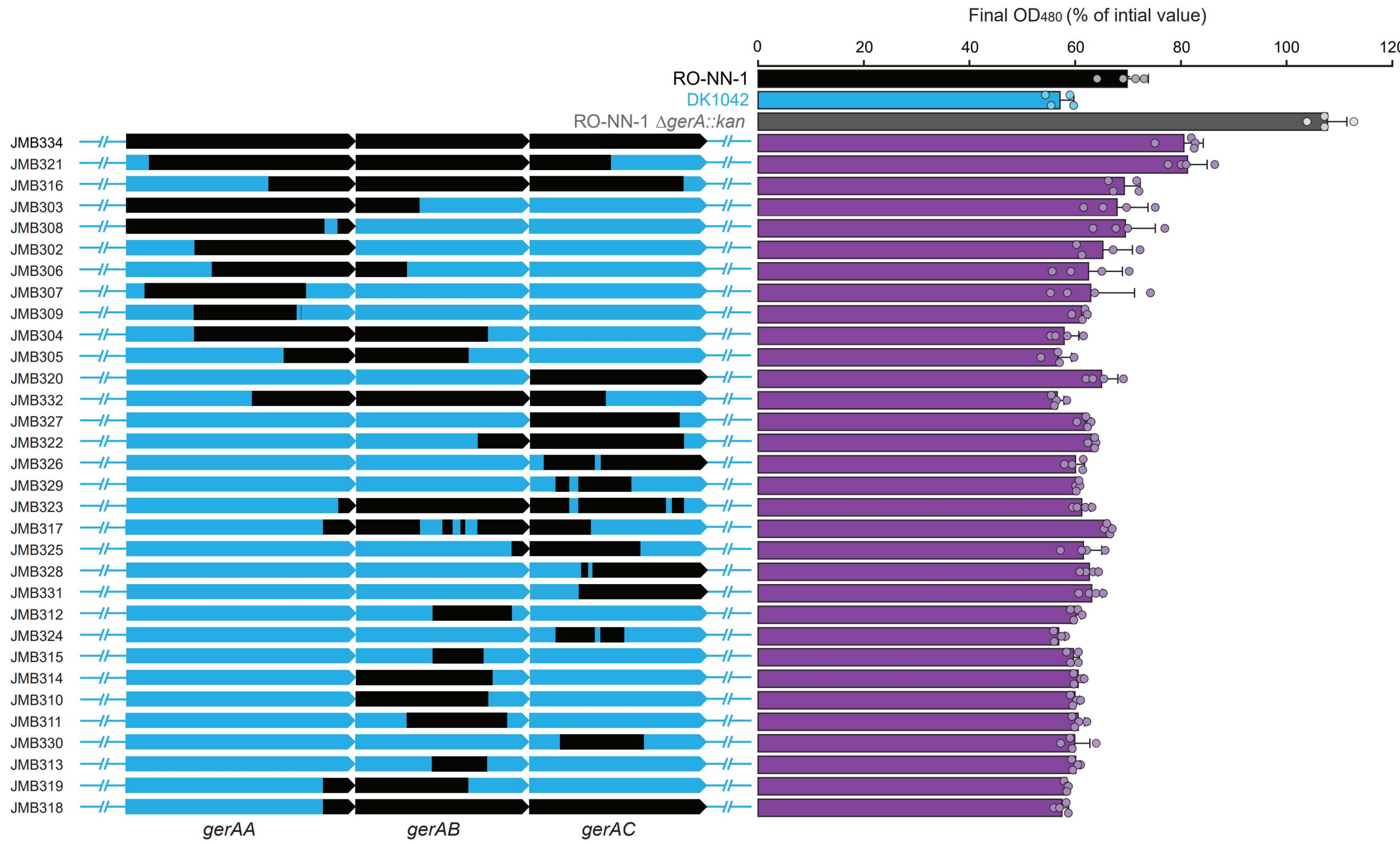

Supplementary Fig. 15: Spore germination of strains with genetic swaps within the *gerA* locus in response to L-valine. Gene segments within the *gerA* operon in strain DK1042 were replaced with corresponding segments from strain RO-NN-1 using a CRISPR-based approach and their spore germination abilities were evaluated. The bar plot shows optical densities measured after incubation of spores of each strain with 50 mM L-valine for 120 min. Data are presented as a percentage of the initial OD_{480} of the phase-bright spores. $n = 4$ independent experiments. Values are presented as mean ± SD. Blue and black regions indicate sequences originating from *B. subtilis* DK1042 and RO-NN-1, respectively. Source data are provided as a Source Data file.

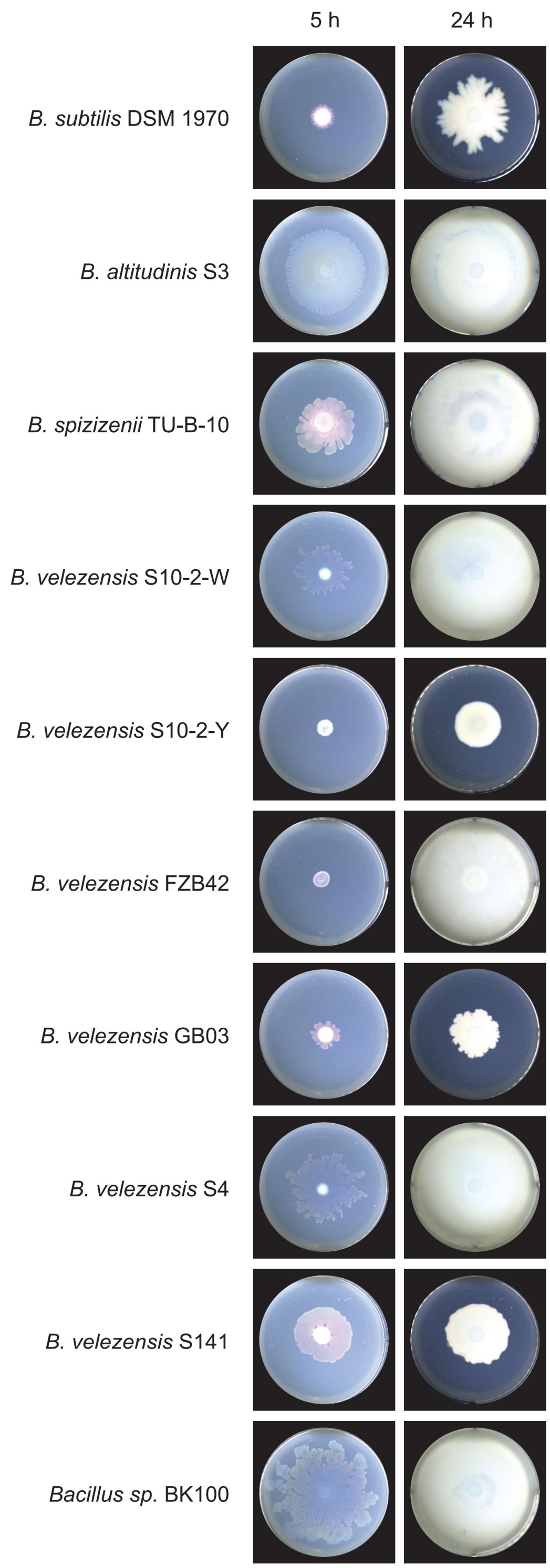

Supplementary Fig. 16: Swarming motility varies among different *Bacillus* isolates. Swarming motility of *Bacillus* isolates was tested on LB medium solidified with 0.7% agar for 5 h and 24 h. Representative images from three independent biological experiments are shown.

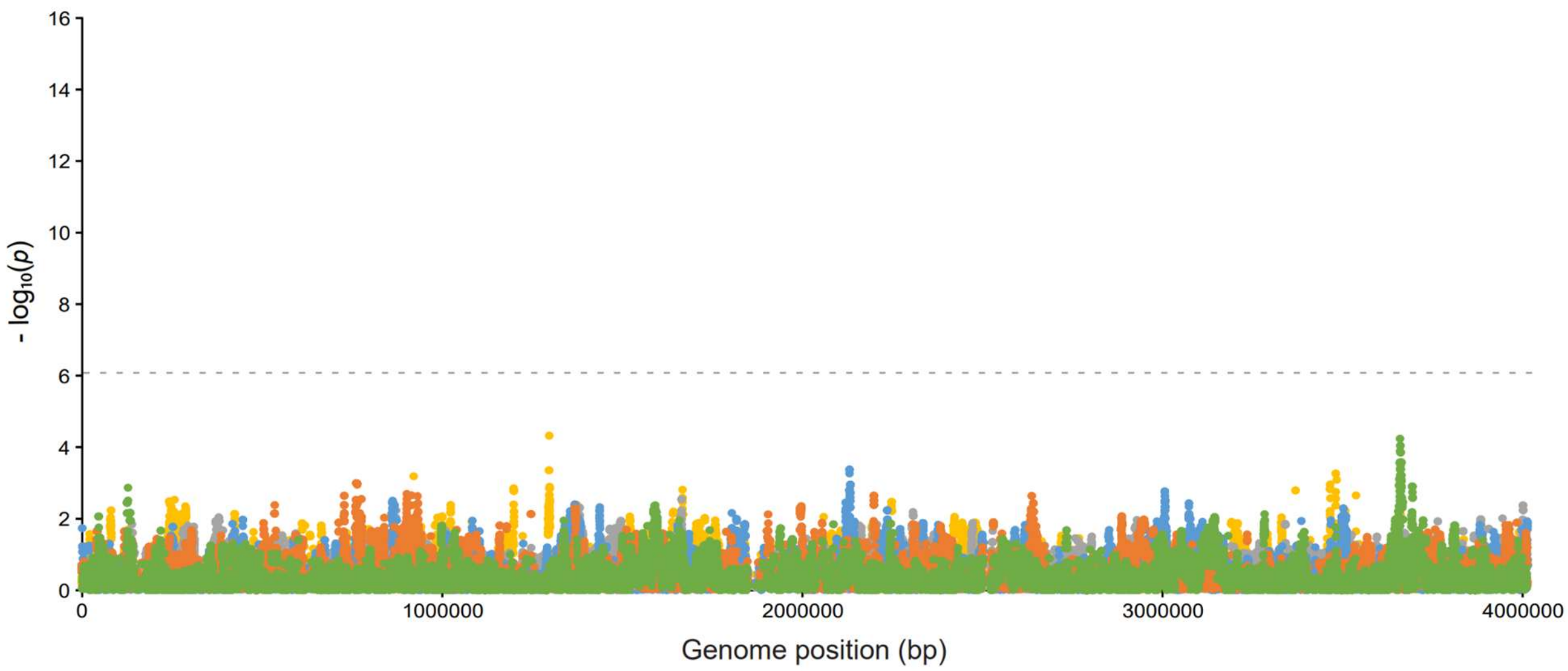

Supplementary Fig. 17: Manhattan plot using randomized swarming motility phenotypic data demonstrates no significant association in the *B. subtilis* QTL population. Intrinsic noise from genetic variation was estimated by running QTL mapping permutations with real genotypic data and randomized quantitative phenotypes. The colors indicate association results from different permutations. The gray dashed line represents the Bonferroni-corrected significance threshold ($p \leq 0.05$).

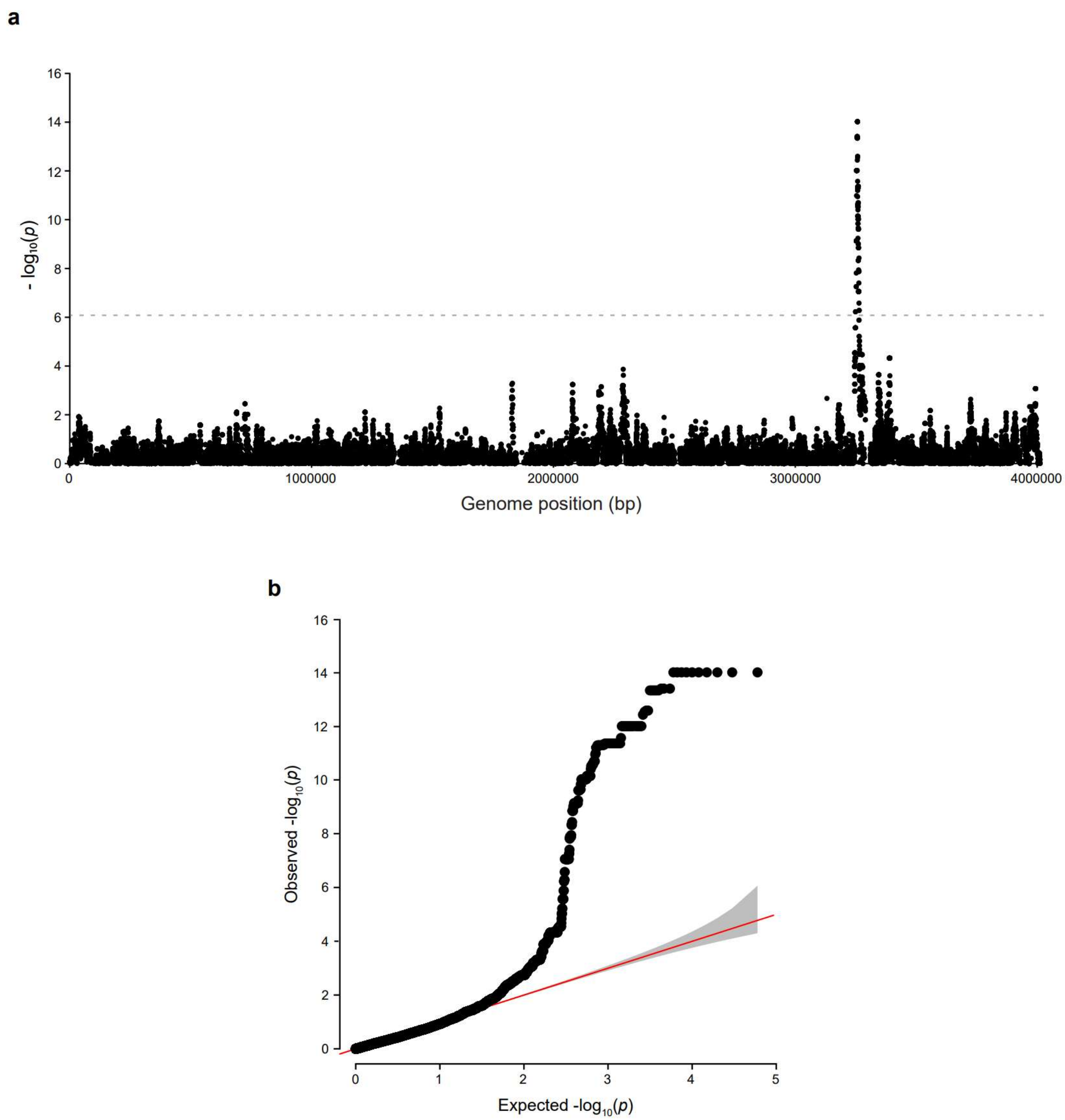

Supplementary Fig. 18: Bacterial QTL mapping identifies a highly significant genomic region associated with swarming motility. (**a**) Manhattan plot summarizing the statistical significance of associations between each genetic variant along the genome and swarming motility across the *B. subtilis* QTL mapping population. An inset showing a 50-kb genomic region including the QTL for swarming motility is presented in Fig. 3d. The gray dashed line represents the Bonferroni-corrected significance threshold ($p \leq 0.05$). (**b**) Q-Q plot corresponding to the Manhattan plot in (**a**).

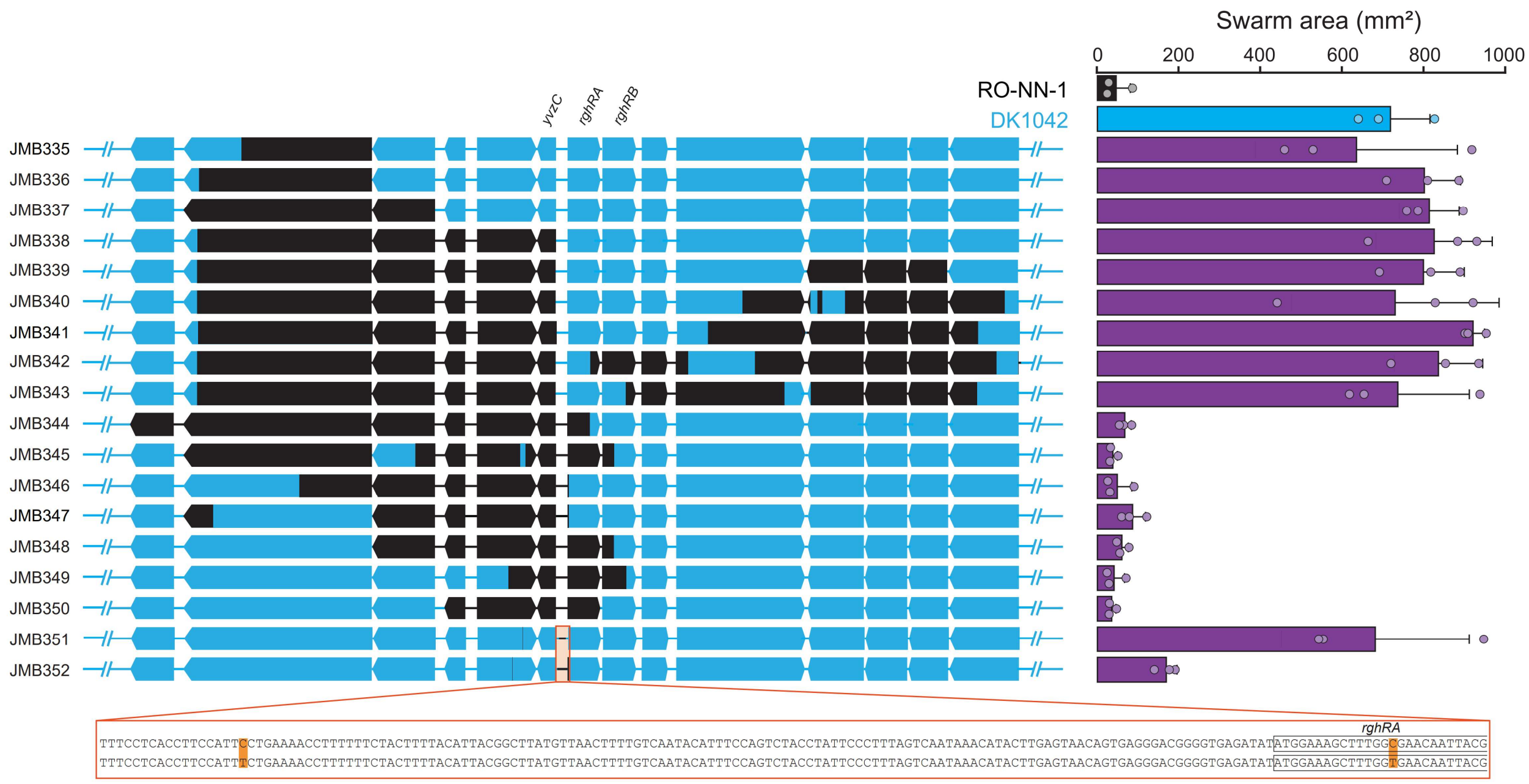

Supplementary Fig. 19: Genetic swaps validate causal genetic loci for swarming motility. Gene segments within the region *smpB*-*opuBA* in strain DK1042 were replaced with corresponding segments from strain RO-NN-1 using a CRISPR-based approach and the swarming motility areas of the resulting strains were measured. The bar plot shows swarm areas measured after incubation for 5 h. The DNA sequence of the genome region containing the two candidate causal genetic variants highlighted in orange is shown. n = 3 independent experiments. Data are presented as mean ± SD. Blue and black regions indicate sequences originating from *B. subtilis* DK1042 and RO-NN-1, respectively. Source data are provided as a Source Data file.

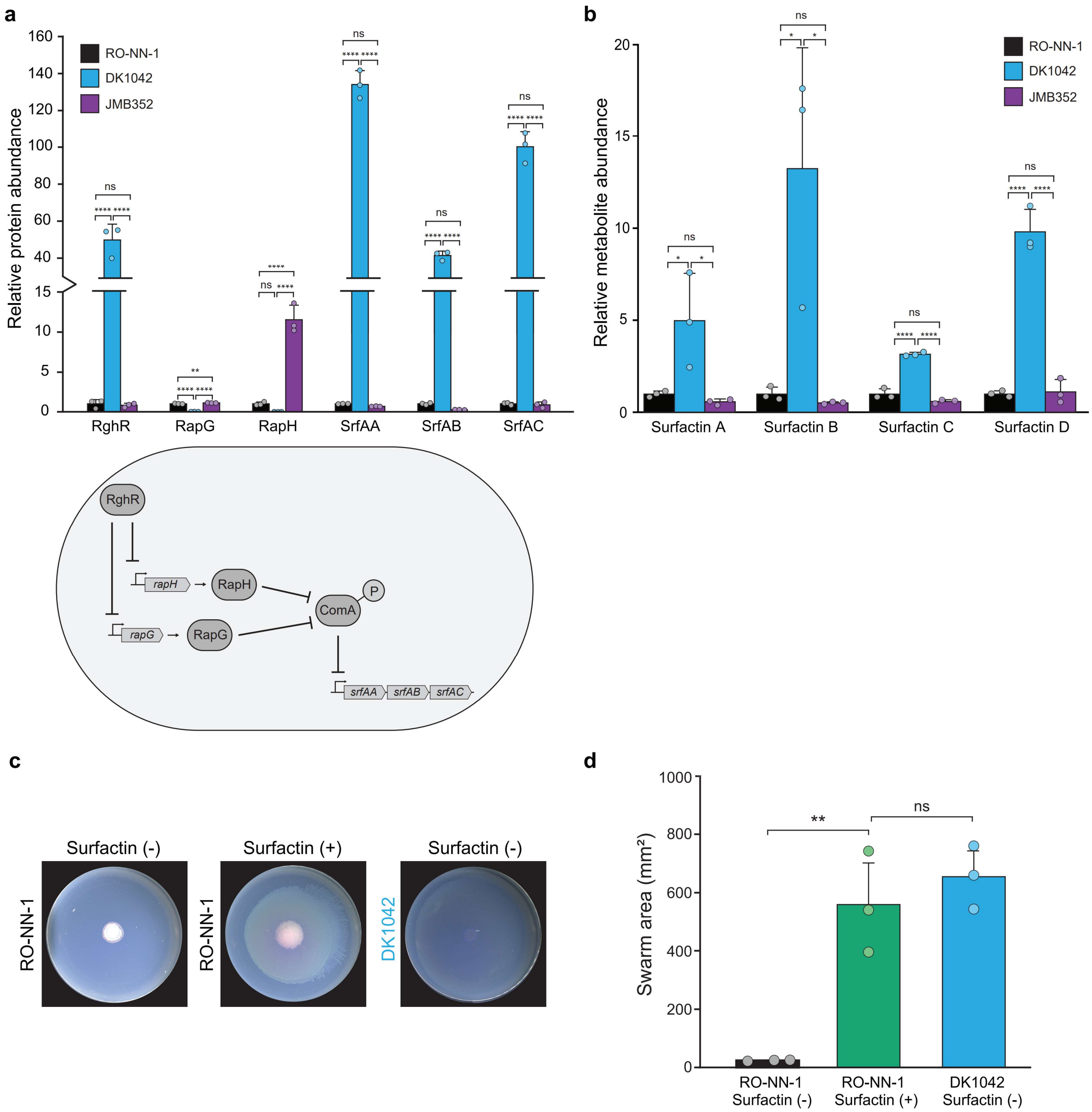

Supplementary Fig. 20: Genetic variation gives rise to differences in surfactin production affecting swarming motility in *B. subtilis* RO-NN-1 and DK1042. (**a**) Expression of surfactin-producing proteins (SrfAA, SrfAB, and SrfAC), RghR, RapG and RapH was monitored through global proteomics of *B. subtilis* RO-NN-1, DK1042 and JMB352 (Supplementary Fig. 19). *n* = 3 independent experiments. Values are presented as mean ± SD. Data were analyzed using one-way ANOVA with Tukey's correction for multiple comparisons. (**b**) Surfactin production was measured in cell pellets from RO-NN-1, DK1042 and JMB352 swarms after 5 h of incubation on LB medium solidified with 0.7% agar. *n* = 3 independent experiments. Values are presented as mean ± SD. Data were analyzed using one-way ANOVA with Tukey's correction for multiple

comparisons. ****: *p* value ≤ 0.0001, **: *p* value ≤ 0.01, *: *p* value ≤ 0.05, ns: not significant. (**c**) Supplementation of surfactin to the agar rescues the swarming-deficient phenotype of strain RO-NN-1. Comparison of swarming motility of *B. subtilis* DK1042 and RO-NN-1 with and without surfactin supplementation over the course of 5 h. Representative images from three independent experiments are shown. (**d**) Areas covered by the DK1042 and RO-NN-1 swarms with and without surfactin supplementation measured after 5 h of incubation on LB medium solidified with 0.7% agar. $n = 3$ independent experiments. Values are presented as mean ± SD. Data were analyzed using one-way ANOVA with Tukey's correction for multiple comparisons. ***p* = 0.0035, ns: not significant. Source data are provided as a Source Data file.

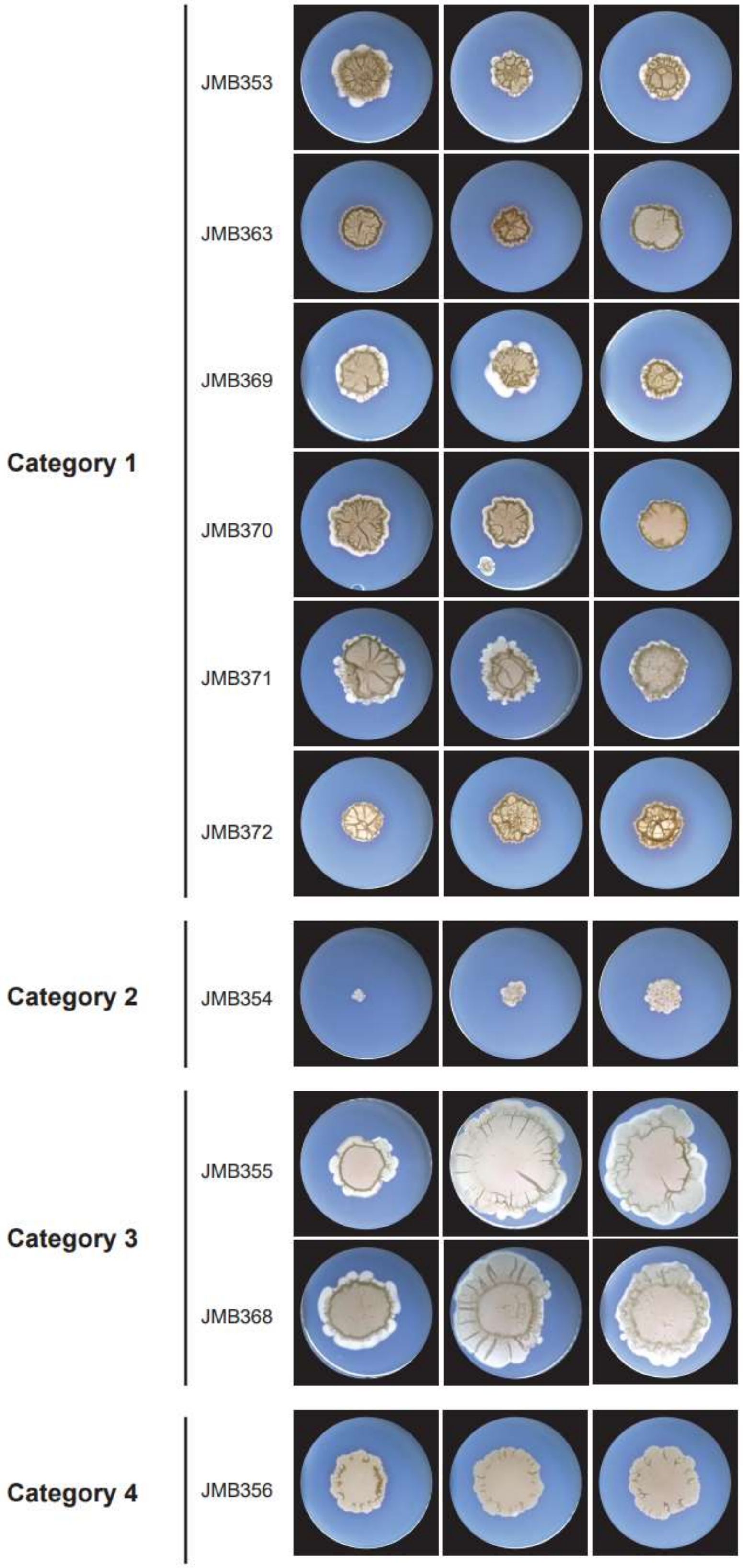

Category 1
JMB353
JMB363
JMB369
JMB370
JMB371
JMB372
Category 2
JMB354
Category 3
JMB355
JMB368
Category 4
JMB356

Continued

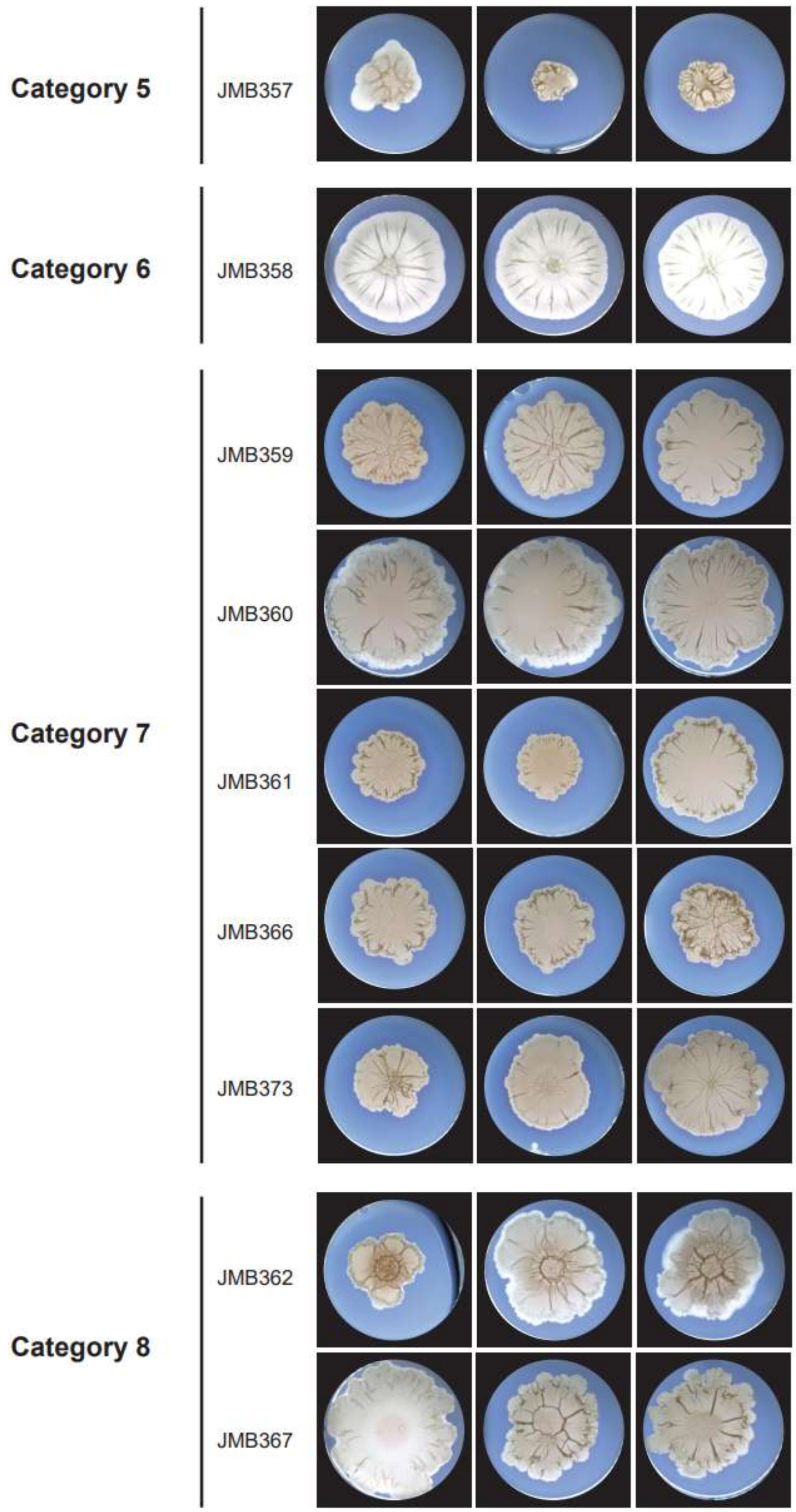
Category 5
JMB357
Category 6
JMB358
JMB359
JMB360
Category 7
JMB361
JMB366
JMB373
JMB362
Category 8
JMB367

Continued

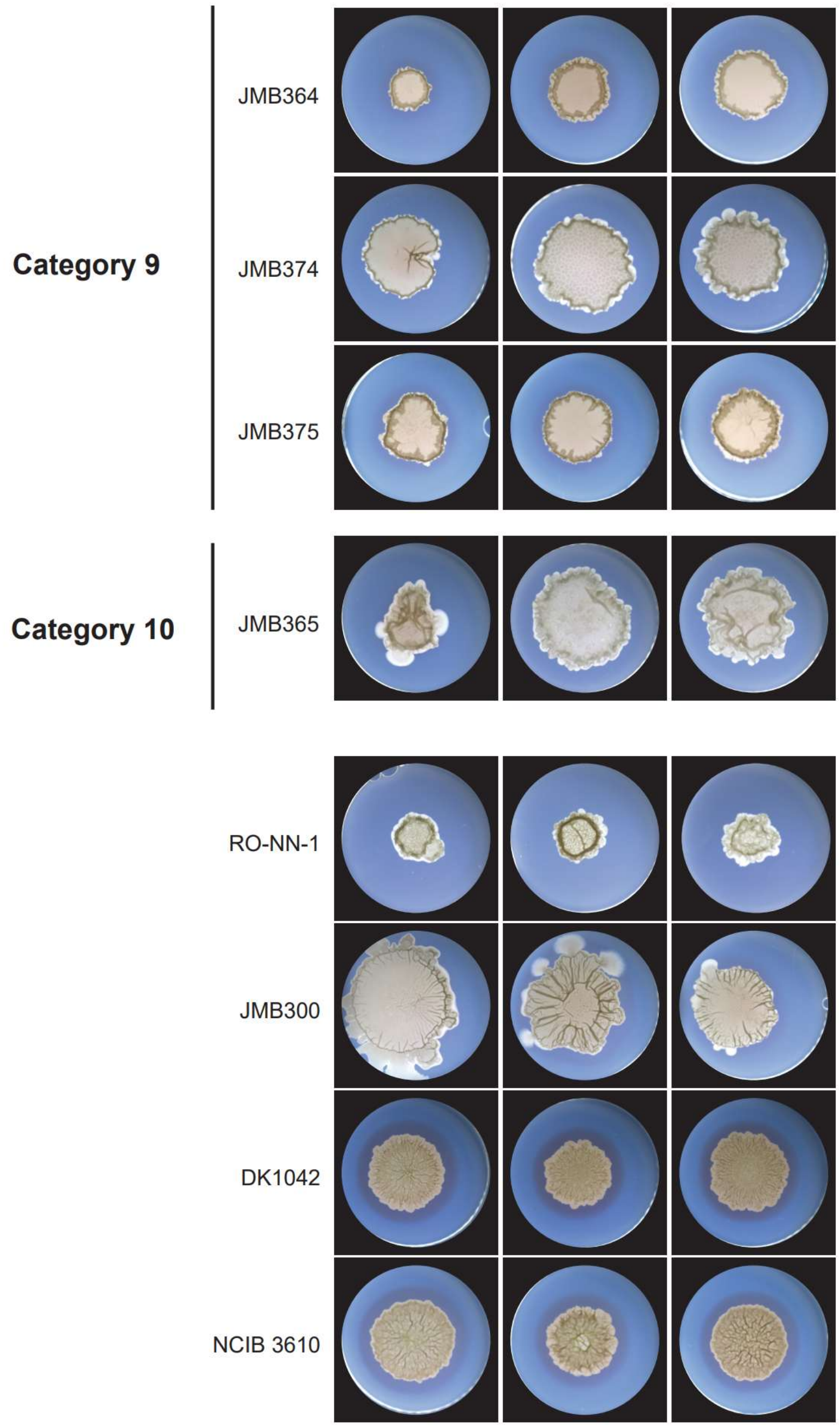

Supplementary Fig. 21: Colony morphology varies across a panel of the most genetically diverse *B. subtilis* shuffled strains from the QTL population. The 23 most genetically diverse shuffled strains (JMB353-375), *B. subtilis* RO-NN-1, JM300, DK1042 and NCIB3610 were grown on biofilm-promoting minimal agar for 72 h. Colonies from three independent biological experiments are shown. The colonies of the shuffled strains were categorized based on their morphology into 10 groups.

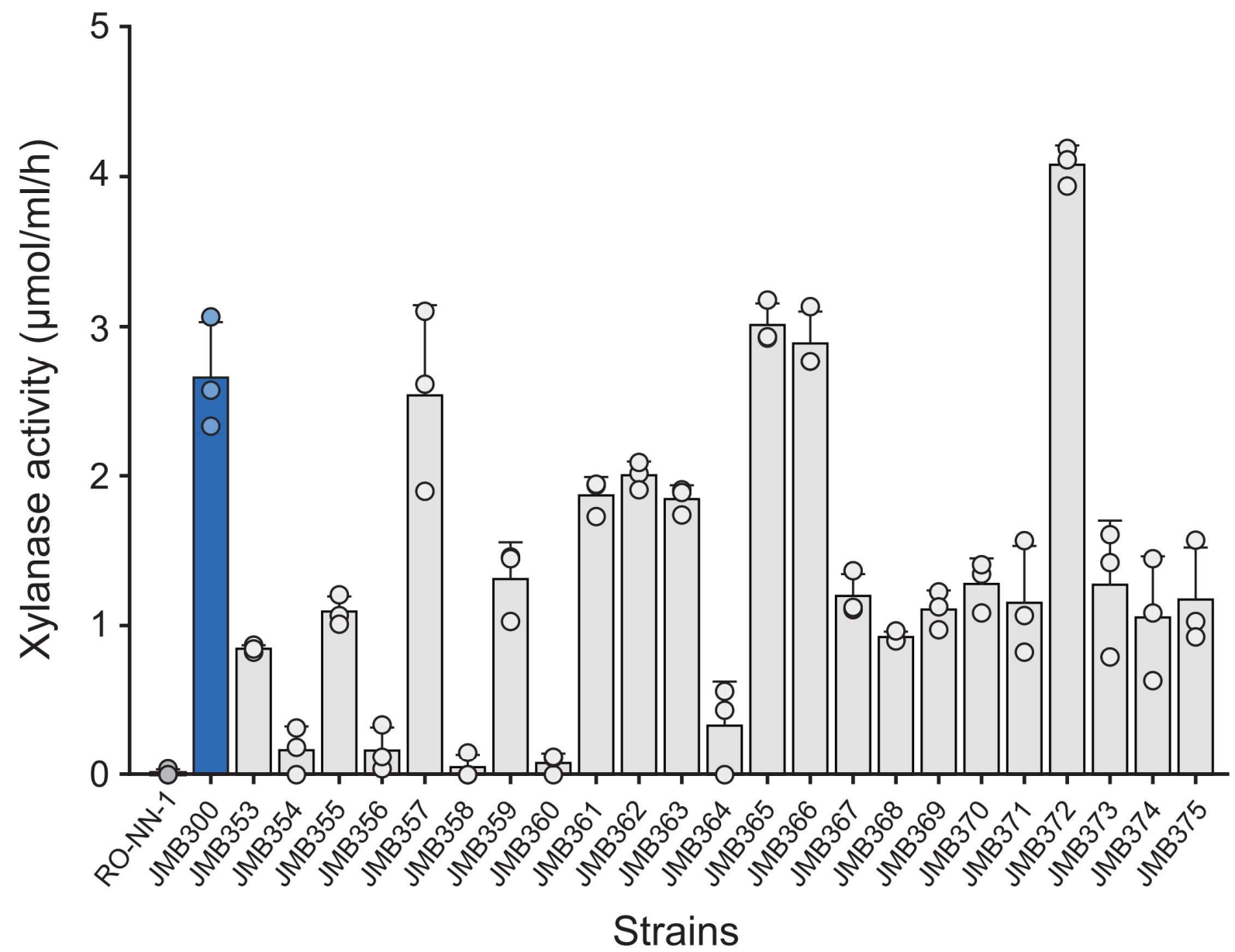

Supplementary Fig. 22: Xylanase activity varies across a panel of the most genetically diverse *B. subtilis* strains from the QTL population. Intrinsic xylanase activity was measured across the 23 most diverse recombinant strains (JMB353-375), *B. subtilis* RO-NN-1 and JMB300. $n = 3$ independent experiments. Data are presented as mean ± SD. Source data are provided as a Source Data file.

.

a

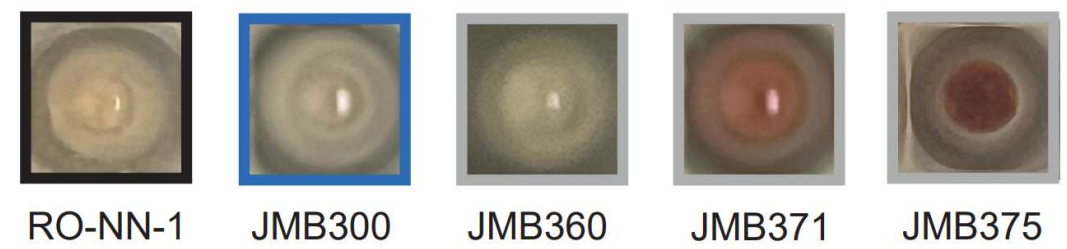

b

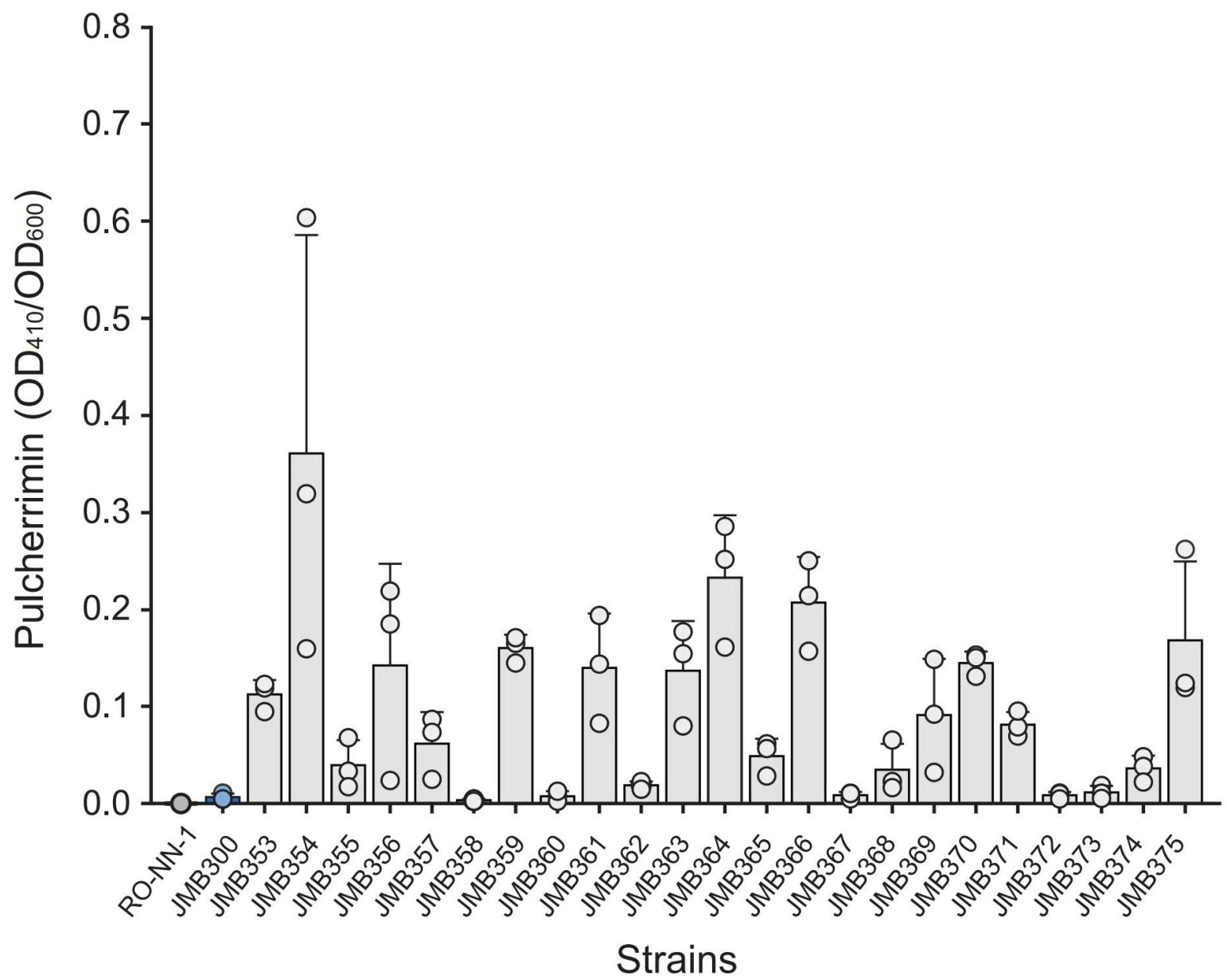

Supplementary Fig. 23: Pulcherrimin production varies across a panel of the most genetically diverse *B. subtilis* shuffled strains from the QTL population. (**a**) Representative images of cell pellets obtained after harvesting cultures from *B. subtilis* RO-NN-1, JMB300 and three recombinant strains (JMB360, JMB371 and JMB375) grown for 48 h in modified LBGM medium supplemented with 0.2 mM $FeCl_3$. Pulcherrimin is insoluble and co-precipitates with the cells after centrifugation. (**b**) Quantification of pulcherrimin production in the 23 most genetically diverse recombinant strains (JMB353-375), *B. subtilis* RO-NN-1 and JMB300. All strains, except for the parent RO-NN-1 and JMB358, carry the genes involved in pulcherrimin biosynthesis and transport (*pchR*-*yvmA* and *yvmC*-*cypX*). n = 3 independent experiments, each with 2 technical replicates. Data are presented as mean ± SD. Source data are provided as a Source Data file.

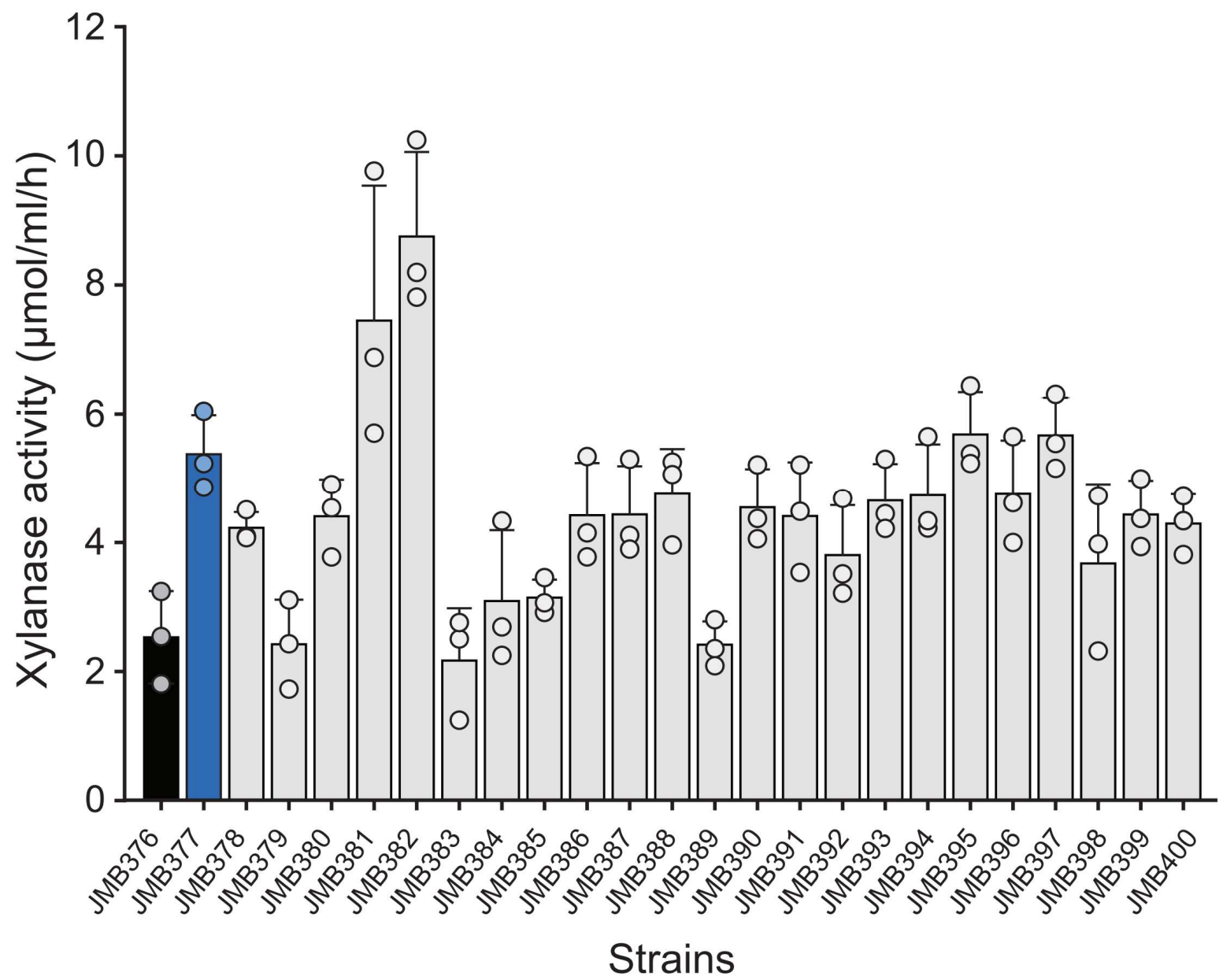

Supplementary Fig. 24: Promoter leakiness varies across a panel of the most genetically diverse shuffled *B. subtilis* strains from the QTL population. Xylanase activity was measured across the 23 most diverse shuffled strains, *B. subtilis* RO-NN-1 and JMB300 harboring pJM609 without induction. *n* = 3 independent experiments. Data are represented as mean ± SD. Source data are provided as a Source Data file.

a

b

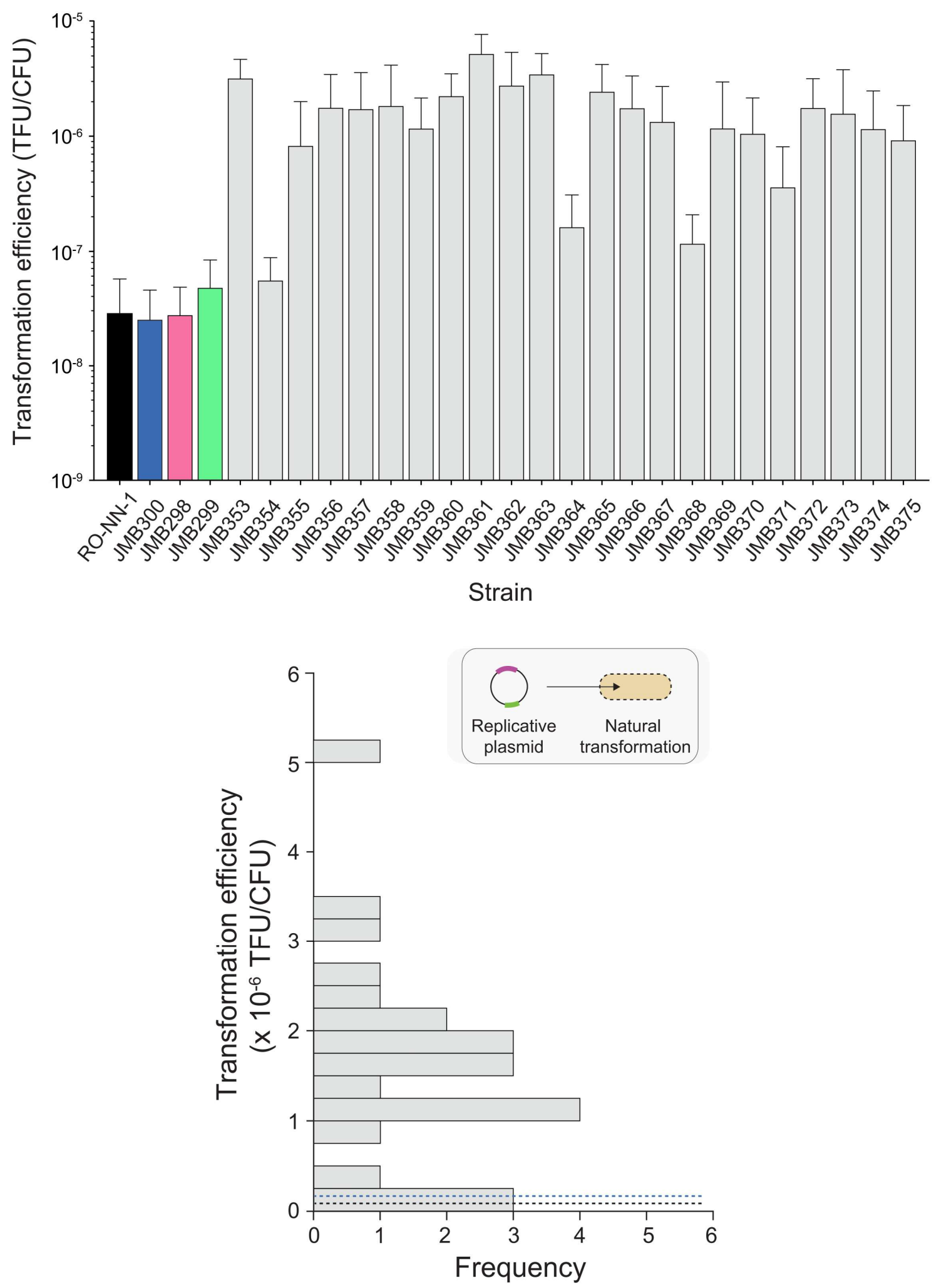

Supplementary Fig. 25: Transformation efficiency varies across a panel of the most genetically diverse *B. subtilis* shuffled strains from the QTL population. (**a**) Cells of the 23 most genetically diverse shuffled strains (JMB353-375), *B. subtilis* RO-NN-1, JMB298, JMB299, and JM300 were transformed with the replicative plasmid pIDV40 via natural competency. Transformation efficiency was calculated by dividing the number of transformants (TFU) by the total number of viable cells (CFU) per milliliter. (**b**) Distribution of transformation efficiency phenotypes. A histogram of transformation efficiencies is shown. Black and dark blue dashed lines indicate phenotypic data from RO-NN-1 and the cured DK1042 strain JM300, respectively. $n \geq 4$ independent experiments. Data are represented as mean ± SD. Source data are provided as a Source Data file.

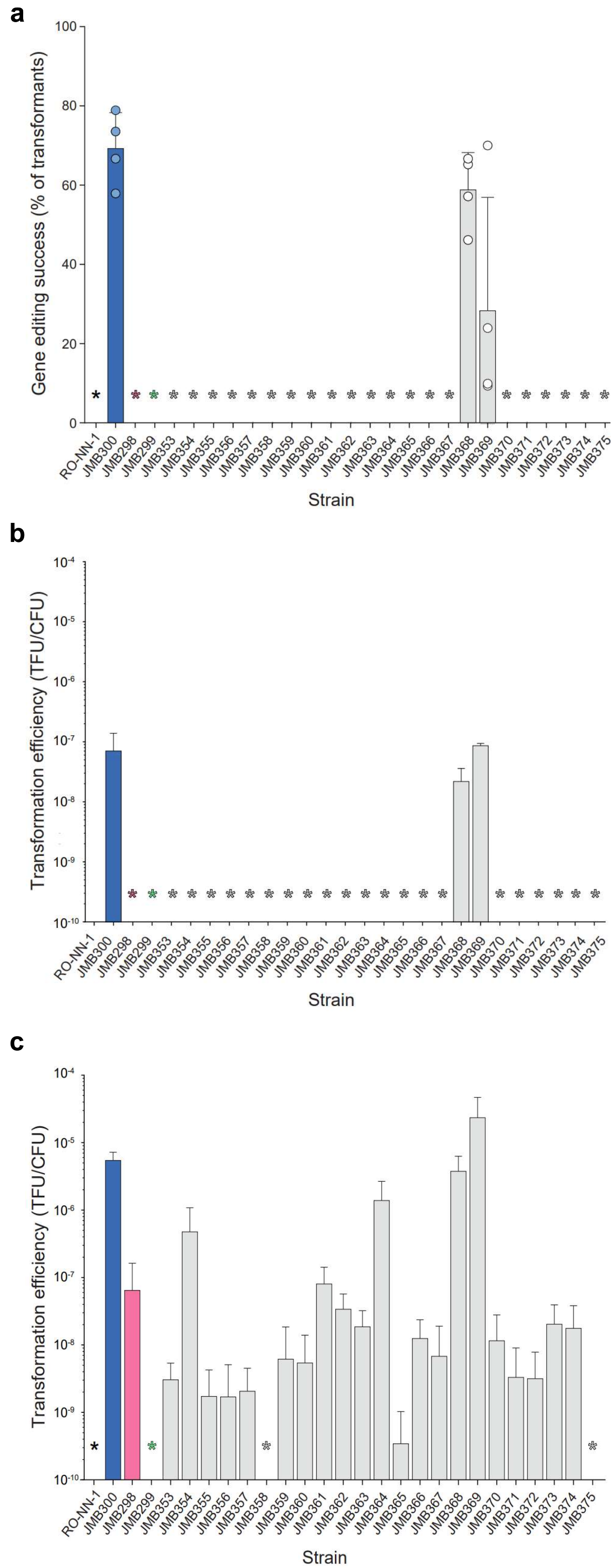

Supplementary Fig. 26: CRISPR-Cas9 gene editing varies across a panel of the most genetically diverse *B. subtilis* shuffled strains from the QTL population. Cells of the 23 most genetically diverse shuffled strains, *B. subtilis* RO-NN-1, JMB298, JMB299, and JM300 were

transformed with (**a**) pJM607 ($n \geq 3$ independent experiments), (**b**) pJM608 ($n \geq 3$ independent experiments) and (**c**) JOE9658.1 [5] ($n \geq 2$ independent experiments) via natural competence. Gene editing efficiency was determined by estimating the percentage of tryptophan auxotrophs among transformants. Transformation efficiency was calculated by dividing the number of transformants (TFU) by the total number of viable cells (CFU) per milliliter. Strains in which no transformants were detected are indicated by asterisks. Data are represented as mean ± SD. Source data are provided as a Source Data file.

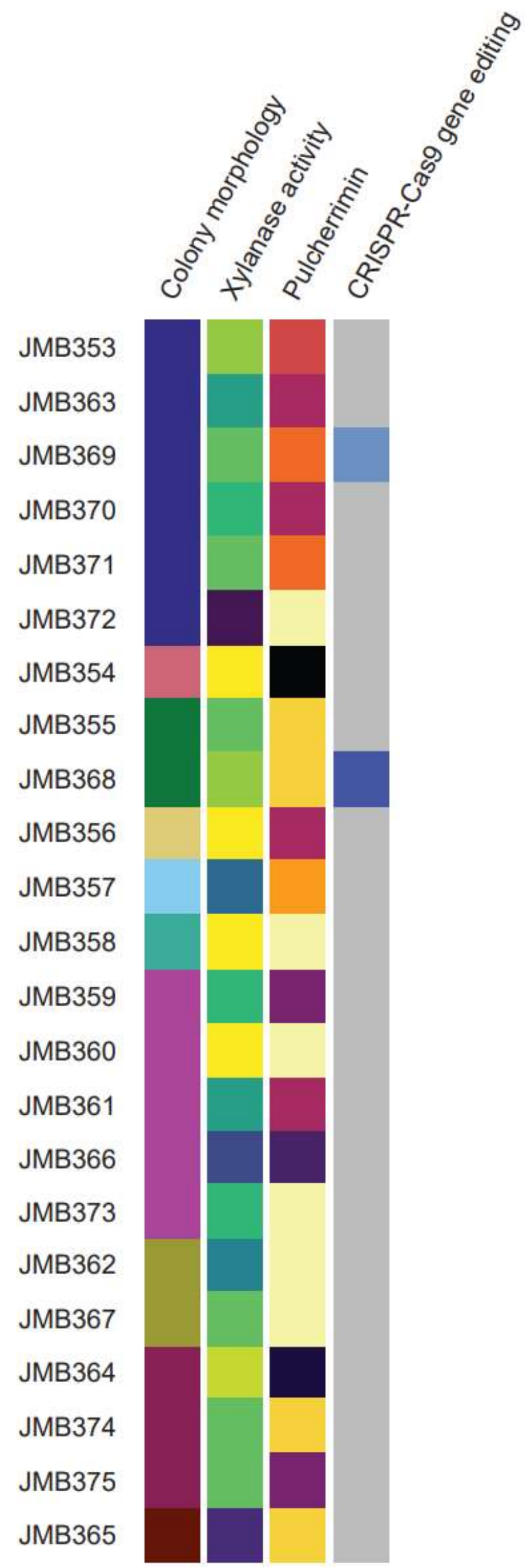

Supplementary Fig. 27: Phenotypic variability across a panel of the 23 most genetically diverse *B. subtilis* shuffled strains from the QTL population. Colors in the colony morphology panel represent phenotype categories as defined in Supplementary Fig. 21. Heat map colors indicate binned values for xylanase activity, pulcherrimin production, and CRISPR-Cas9 gene editing efficiency, categorized based on the distributions shown in the histograms in Fig. 4b–d.

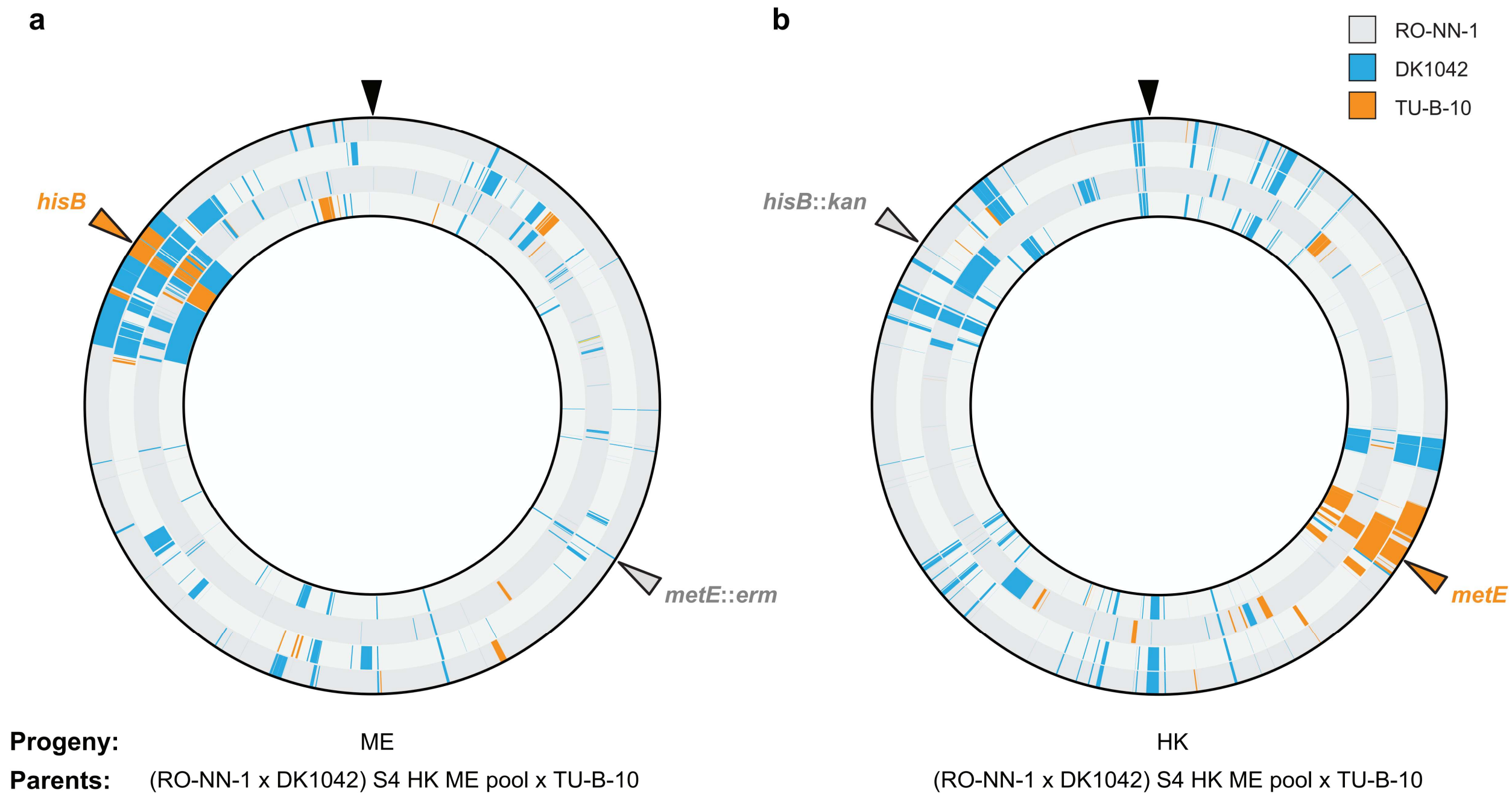

Supplementary Fig. 28: Multiparental genome shuffling can maximize allelic diversity in bacterial QTL mapping populations. Crossing the *B. subtilis* S4 HK ME pool (Supplementary Fig. 1) and *B. spizizenii* TU-B-10 generated (**a**) ME and (**b**) HK progeny. Each concentric circle represents an individual sequenced shuffled strain from that cross. Blue bars indicate recombination fragments originating from DK1042. Orange bars represent sequences recombined from strain TU-B-10. The remaining genomic sequences in gray come from strain RO-NN-1. Orange and gray arrows indicate locations of the selection markers. Black arrows indicate the origin of replication. ME: Δ*metE*::*erm*; HK: Δ*hisB*::*kan*.

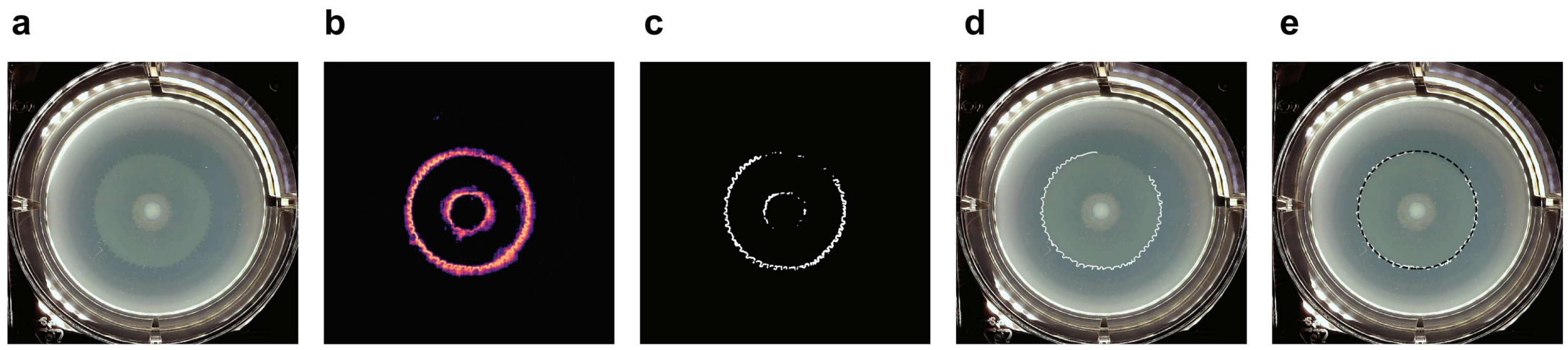

Supplementary Fig. 29: Swarm image analysis workflow. (**a**) A convolutional neural network (CNN) inputs an image of a *B. subtilis* swarm in a single well of a 6-well plate. (**b**) The CNN outputs pixel-level probabilities for swarm boundary classification. **(c)** A global threshold is applied to the probability values to create a binary mask. (**d**) The longest continuous segment of the thresholded mask is selected for circle fitting. (**e**) The best fit circle is computed based on the longest segment and subsequently used to estimate swarming phenotypes.

Supplementary Table 1. Strains used in this study.

Strain	Genotype	Reference	Figures
Bacillus subtilis subsp. *subtilis* RO-NN-1	Wild type	[6,7]	1-4, S1, S2, S3, S5, S6, S12, S14, S15, S19-25, S28
Bacillus subtilis subsp. *subtilis* NCIB3610	Wild type	[8,9]	S21
Bacillus subtilis DK1042	NCIB3610 *comI*^{Q12L}	[10]	1-3, S1, S2, S3, S5, S6, S12, S14, S15, S19, S20, S21, S28
B. subtilis DSM 1970	Wild type		S16
B. spizizenii TU-B-10	Wild type	[6,11,12]	S16, S28
B. altitudinis S3	Wild type		S16
B. velezensis S10-2-W	Wild type	[13]	S16
B. velezensis S10-2-Y	Wild type		S16
B. velezensis FZB42	Wild type	[14,15]	S16
B. velezensis GB03	Wild type	[16]	S16
B. velezensis S4	Wild type	[13]	S16
B. velezensis S141	Wild type	[17]	S16
Bacillus sp. BK100	Wild type	[18]	S16
Clostridium thermocellum DSM 1313	Wild type	[19]	
Clostridium thermocellum S14	Wild type	[20]	5
Stutzerimonas stutzeri A1501	Wild type	[21]	
Stutzerimonas stutzeri DSM 4166	Wild type	[22,23]	5
Novosphingobium aromaticivorans F199	Wild type	[24]	
Novosphingobium aromaticivorans sp. B0695	Wild type	[25]	5

BKK34900	168 Δ*hisB*::*kan*	[26]	
BKE13180	168 Δ*metE*::*erm*	[26]	
JMB1	RO-NN-1 Δ*hisB*::*kan*	[27]	
JMB3	RO-NN-1 Δ*metE*::*erm*	[27]	S1
JMB60	RO-NN-1 Δ*hisB*::*kan* Δ*metE*::*erm*	[27]	S1
JMB90	DK1042 Δ*metE*::*erm*	This study	S1
JMB91	DK1042 Δ*hisB*::*kan* Δ*metE*::*erm*	This study	S1
JMB92	DK1042 Δ*hisB*::*erm*	This study	S1
JMB298	RO-NN-1 Δ*hsdR*	[27]	S25, S26
JMB299	RO-NN-1 Δ*mrr*	[27]	S25, S26
JMC1	DSM 1313 *trpC*::*cat*	This study	
JMC2	DSM 1313 *pyrF*::*neo trpC*::*cat*	This study	5
JMN150	F199 *hisB*::*kan*	This study	
JMN154	F199 *hisB*::*kan leuB*::*cat*	This study	5
JMS1	A1501 *hisB*::*kan*	This study	
JMS2	A1501 *trpC*::*apr hisB*::*kan*	This study	5
JMB241-JMB264	S1 Δ*metE*::*erm* pool x DK1042 Δ*hisB*::*kan* → S2 $hisB^{+}$ $metE^{+}$	This study	1, S1
JMB300	NCIB 3610 lacking pBS32	This study	4, S21-23, S25, S26
JMB301	RO-NN-1 Δ*gerA*::*kan*	This study	S14, S15
JMB302	RO-NN-1 nt 3,188,758- nt 3,189,735 replaces DK1042 nt 3,391,225- nt 3,392,202 (*gerAA* nt 429- *gerAA* nt 1,406)	This study	S12, S15
JMB303	RO-NN-1 nt 3,188,407- nt 3,190,148 replaces DK1042 nt 3,390,874- nt 3,392,615 (*gerAA* nt 78- *gerAB* nt 402)	This study	S12, S15
JMB304	RO-NN-1 nt 3,188,758- nt 3,190,580 replaces DK1042 nt 3,391,225- nt 3,393,047 (*gerAA* nt 429- *gerAB* nt 834)	This study	S12, S15

JMB305	RO-NN-1 nt 3,189,323- nt 3,190,458 replaces DK1042 nt 3,391,790- nt 3,392,925 (*gerAA* nt 994- *gerAB* nt 712)	This study	2, S12, S13, S15
JMB306	RO-NN-1 nt 3,188,869- nt 3,190,070 replaces DK1042 nt 3,391,336- nt 3,392,537 (*gerAA* nt 540- *gerAB* nt 324)	This study	S12, S15
JMB307	RO-NN-1 nt 3,188,471- nt 3,189,461 replaces DK1042 nt 3,390,938- nt 3,391,928 (*gerAA* nt 142- *gerAA* nt 1,132)	This study	S12, S15
JMB308	RO-NN-1 nt 3,188,407- nt 3,189,580 replaces DK1042 nt 3,390,874- nt 3,392,047 (*gerAA* nt 78- *gerAA* nt 1,251)<br><br>RO-NN-1 nt 3,189,661- nt 3,189,735 replaces DK1042 nt 3,392,128- nt 3,392,202 (*gerAA* nt 1,332- *gerAA* nt 1,406)	This study	2, S12, S15
JMB309	RO-NN-1 nt 3,188,758- nt 3,189,406 replaces DK1042 nt 3,391,225- nt 3,391,873 (*gerAA* nt 429- *gerAA* nt 1,077)<br><br>RO-NN-1 nt 3,189,461 replaces DK1042 nt 3,391,928 (*gerAA* nt 1,132)	This study	S12, S15
JMB310	RO-NN-1 nt 3,190,064- nt 3,190,580 replaces DK1042 nt 3,392,531- nt 3,393,047 (*gerAB* nt 318- *gerAB* nt 834)	This study	S12, S15
JMB311	RO-NN-1 nt 3,190,070- nt 3,190,702 replaces DK1042 nt 3,392,537- nt 3,393,169 (*gerAB* nt 324- *gerAB* nt 956)	This study	2, S12, S15
JMB312	RO-NN-1 nt 3,190,230- nt 3,190,727 replaces DK1042 nt 3,392,697- nt 3,393,194 (*gerAB* nt 484- *gerAB* nt 981)	This study	S12, S15
JMB313	RO-NN-1 nt 3,190,230- nt 3,190,580 replaces DK1042 nt 3,392,697- nt 3,393,047	This study	S12, S15

	(*gerAB* nt 484- *gerAB* nt 834)		
JMB314	RO-NN-1 nt 3,190,064- nt 3,190,607 replaces DK1042 nt 3,392,531- nt 3,393,074 (*gerAB* nt 318- *gerAB* nt 861)	This study	S12, S15
JMB315	RO-NN-1 nt 3,190,230- nt 3,190,550 replaces DK1042 nt 3,392,697- nt 3,393,017 (*gerAB* nt 484- *gerAB* nt 804)	This study	S12, S15
JMB316	RO-NN-1 nt 3,189,223- nt 3,191,809 replaces DK1042 nt 3,391,690- nt 3,394,276 (*gerAA* nt 894- *gerAC* nt 969)	This study	2, S12, S13, S15
JMB317	RO-NN-1 nt 3,189,572- nt 3,190,148 replaces DK1042 nt 3,392,039- nt 3,392,615 (*gerAA* nt 1,243- *gerAB* nt 402)<br><br>RO-NN-1 nt 3,190,290- nt 3,190,355 replaces DK1042 nt 3,392,757- nt 3,392,822 (*gerAB* nt 544- *gerAB* nt 609)<br><br>RO-NN-1 nt 3,190,403- nt 3,190,436 replaces DK1042 nt 3,392,870- nt 3,392,903 (*gerAB* nt 657- *gerAB* nt 690)<br><br>RO-NN-1 nt 3,190,511- nt 3,191,224 replaces DK1042 nt 3,392,978- nt 3,393,691 (*gerAB* nt 765- *gerAC* nt 384)	This study	2, S12, S13, S15
JMB318	RO-NN-1 nt 3,189,572- nt 3,191,924 replaces DK1042 nt 3,392,039- nt 3,394,391 (*gerAA* nt 1,243- *gerAC* nt 1,084)	This study	2, S12, S13, S15
JMB319	RO-NN-1 nt 3,189,572- nt 3,190,458 replaces DK1042 nt 3,392,039- nt 3,392,925 (*gerAA* nt 1,243- *gerAB* nt 712)	This study	S12, S15
JMB320	RO-NN-1 nt 3,190,867- nt 3,191,924 replaces DK1042 nt 3,393,334- nt 3,394,391 (*gerAC* nt 27- *gerAC* nt 1,084)	This study	S12, S15
JMB321	RO-NN-1 nt 3,188,471- nt 3,191,315 replaces DK1042 nt 3,390,938- nt 3,393,818	This study	S12, S15

	(*gerAA* nt 142- *gerAC* nt 511)		
JMB322	RO-NN-1 nt 3,190,511- nt 3,191,809 replaces<br>DK1042 nt 3,392,987- nt 3,394,276<br>(*gerAB* nt 765- *gerAC* nt 969)	This study	2, S12, S13, S15
JMB323	RO-NN-1 nt 3,189,661- nt 3,191,086 replaces<br>DK1042 nt 3,392,128- nt 3,393,553<br>(*gerAA* nt 1,332- *gerAC* nt 246)<br><br>RO-NN-1 nt 3,191,146- nt 3,191,695 replaces<br>DK1042 nt 3,393,613- nt 3,394,162<br>(*gerAC* nt 306- *gerAC* nt 855)<br><br>RO-NN-1 nt 3,191,732- nt 3,191,809 replaces<br>DK1042 nt 3,394,199- nt 3,394,276<br>(*gerAC* nt 892- *gerAC* nt 969)	This study	2, S12, S13, S15
JMB324	RO-NN-1 nt 3,191,001- nt 3,191,248 replaces<br>DK1042 nt 3,393,468- nt 3,393,715<br>(*gerAC* nt 161- *gerAC* nt 408)<br><br>RO-NN-1 nt 3,191,284- nt 3,191,434 replaces<br>DK1042 nt 3,393,751- nt 3,393,901<br>(*gerAC* nt 444- *gerAC* nt 594)	This study	S12, S15
JMB325	RO-NN-1 nt 3,190,727- nt 3,191,536 replaces<br>DK1042 nt 3,393,194- nt 3,394,003<br>(*gerAB* nt 981- *gerAC* nt 696)	This study	S12, S15
JMB326	RO-NN-1 nt 3,190,927- nt 3,191,248 replaces<br>DK1042 nt 3,393,394- nt 3,393,715<br>(*gerAC* nt 87- *gerAC* nt 408)<br><br>RO-NN-1 nt 3,191,284- nt 3,191,924 replaces<br>DK1042 nt 3,393,751- nt 3,394,391<br>(*gerAC* nt 444- *gerAC* nt 1084)	This study	S12, S15
JMB327	RO-NN-1 nt 3,190,867- nt 3,191,782 replaces<br>DK1042 nt 3,393,334- nt 3,394,249<br>(*gerAC* nt 27- *gerAC* nt 942)	This study	2, S12, S13, S15
JMB328	RO-NN-1 nt 3,191,162- nt 3,191,209 replaces<br>DK1042 nt 3,393,629- nt 3,393,676	This study	S12, S15

	(*gerAC* nt 322- *gerAC* nt 369)<br><br>RO-NN-1 nt 3,191,234- nt 3,191,924 replaces<br>DK1042 nt 3,393,701- nt 3,394,391 (*gerAC* nt 394- *gerAC* nt 1084)		
JMB329	RO-NN-1 nt 3,191,001- nt 3,191,086 replaces<br>DK1042 nt 3,393,468- nt 3,393,553 (*gerAC* nt 161- *gerAC* nt 246)<br><br>RO-NN-1 nt 3,191,146- nt 3,191,476 replaces<br>DK1042 nt 3,393,613- nt 3,393,943 (*gerAC* nt 306- *gerAC* nt 636)	This study	2, S12, S13, S15
JMB330	RO-NN-1 nt 3,391,035- nt 3,191,564 replaces<br>DK1042 nt 3,393,502- nt 3,394,031 (*gerAC* nt 195- *gerAC* nt 724)	This study	2, S12, S13, S15
JMB331	RO-NN-1 nt 3,391,146- nt 3,191,924 replaces<br>DK1042 nt 3,393,613- nt 3,394,391 (*gerAC* nt 306- *gerAC* nt 1,084)	This study	2, S12, S13, S15
JMB332	RO-NN-1 nt 3,389,121- nt 3,191,317 replaces<br>DK1042 nt 3,391,588- nt 3,393,784 (*gerAA* nt 792- *gerAC* nt 477)	This study	S12, S15
JMB333	RO-NN-1 nt 3,188,407- nt 3,191,104 replaces<br>DK1042 nt 3,390,874- nt 3,393,571 (*gerAA* nt 78- *gerAC* nt 264)	This study	S12, S15
JMB334	RO-NN-1 nt 3,188,407- nt 3,191,924 replaces<br>DK1042 nt 3,390,874- nt 3,394,391 (*gerAA* nt 78- *gerAC* nt 1,084)	This study	2, S12, S13, S15
JMB335	RO-NN-1 nt 3,253,390- nt 3,254,911 replaces<br>DK1042 nt 3,452,597- nt 3,454,118 (*rnr* nt 99 - *rnr* nt 1,620)	This study	S19
JMB336	RO-NN-1 nt 3,252,877- nt 3,254,911 replaces<br>DK1042 nt 3,452,084- nt 3,454,118 (*rnr* nt 99 - *rnr* nt 2,133)	This study	S19
JMB337	RO-NN-1 nt 3,252,787- nt 3,255,535 replaces<br>DK1042 nt 3,451,994- nt 3,454,742	This study	S19

	(*estA* nt 234- *rnr* nt 2,223)		
JMB338	RO-NN-1 nt 3,252,877- nt 3,257,184 replaces DK1042 nt 3,452,084- nt 3,456,391 (*yvzC* nt 139- *rnr* nt 2,133)	This study	3, S19
JMB339	RO-NN-1 nt 3,252,877- nt 3,257,184 replaces DK1042 nt 3,452,084- nt 3,456,391 (*yvzC* nt 139- *rnr* nt 2,133)<br><br>RO-NN-1 nt 3,260,600- nt 3,262,848 replaces DK1042 nt 3,459,816 - nt 3,462,064 (*opuBB* nt 39- 4 bp downstream of *opuBD*)	This study	S19
JMB340	RO-NN-1 nt 3,252,877- nt 3,257,184 replaces DK1042 nt 3,452,084- nt 3,456,391 (*yvzC* nt 139- *rnr* nt 2,133)<br><br>RO-NN-1 nt 3,259,739- nt 3,260,640 replaces DK1042 3,458,955- nt 3,459,856 (*yvaQ* nt 876- *opuBD* nt 645)<br><br>RO-NN-1 nt 3,260,715- nt 3,260,772 replaces DK1042 nt 3,459,931- nt 3,459,988 (*opuBD* nt 513- *opuBD* nt 570)<br><br>RO-NN-1 nt 3,261,057- nt 3,263,821 replaces DK1042 nt 3,460,273- nt 3,463,037 (*opuBA* nt 228- *opuBD* nt 228)	This study	S19
JMB341	RO-NN-1 nt 3,252,877- nt 3,257,184 replaces DK1042 nt 3,452,084- nt 3,456,391 (*yvzC* nt 139- *rnr* nt 2,133)<br><br>RO-NN-1 nt 3,259,268- nt 3,260,540 replaces DK1042 nt 3,458,484- nt 3,459,756 (*yvaQ* nt 405- *yvaQ* nt 1,677)<br><br>RO-NN-1 nt 3,260,600- nt 3,263,363 replaces DK1042 nt 3,459,816- nt 3,462,579 (36 bp downstream of *yvaQ*- *opuBA* nt 686)	This study	S19
JMB342	RO-NN-1 nt 3,252,877- nt 3,257,184 replaces DK1042 nt 3,452,084- nt 3,456,391	This study	3, S19

	(*yvzC* nt 139- *rnr* nt 2,133)<br><br>RO-NN-1 nt 3,257,750- nt 3,259,025<br>replaces<br>DK1042 nt 3,456,962- nt 3,458,241<br>(*rghRA* nt 285 - *yvaQ* nt 162)<br><br>RO-NN-1 nt 3,259,739- nt 3,263,671<br>replaces<br>DK1042 nt 3,458,955- nt 3,462,887<br>(*yvaQ* nt 876- *opuBA* nt 378)<br><br>RO-NN-1 nt 3,264,025- nt 3,264,091<br>replaces<br>DK1042 nt 3,463,241- nt 3,463,307<br>(43 bp upstream of *opuBA*- *opuBA* nt 24)		
JMB343	RO-NN-1 nt 3,252,877- nt 3,257,184<br>replaces<br>DK1042 nt 3,452,084- nt 3,456,391<br>(*yvzC* nt 139- *rnr* nt 2,133)<br><br>RO-NN-1 nt 3,258,189- nt 3,260,285<br>replaces<br>DK1042 3,457,405- nt 3,459,501<br>(*rghRB* nt 288- *yvaQ* nt 1,422)<br><br>RO-NN-1 nt 3,260,676- nt 3,263,367<br>replaces<br>DK1042 nt 3,459,892- nt 3,462,579<br>(*opuBA* nt 686- *opuBD* nt 609)	This study	3, S19
JMB344	RO-NN-1 nt 3,252,292- nt 3,257,747<br>replaces<br>DK1042 nt 3,451,499- nt 3,456,962<br>(*smpB* nt 234- *rghRA* nt 282)	This study	3, S19
JMB345	RO-NN-1 nt 3,252,787- nt 3,254,911<br>replaces<br>DK1042 nt 3,451,994- nt 3,454,118<br>(*rnr* nt 99- *rnr* nt 2,223)<br><br>RO-NN-1 nt 3,255,535- nt 3,256,708<br>replaces<br>DK1042 3,454,742- nt 3,455,915<br>(*estA* nt 234- *yvaM* nt 430)<br><br>RO-NN-1 nt 3,256,848- nt 3,258,045<br>replaces<br>DK1042 nt 3,456,055- nt 3,457,261<br>(*yvaM* nt 570- *rghRB* nt 144)	This study	S19
JMB346	RO-NN-1 nt 3,254,113-nt 3,257,480<br>replaces<br>DK1042 nt 3,453,320- nt 3,456,695	This study	S19

	(*rnr* nt 897- *rghRA* nt 15)		
JMB347	RO-NN-1 nt 3,252,787- nt 3,253,042 replaces DK1042 nt 3,451,994- nt 3,452,249 (*rnr* nt 1,968- *rnr* nt 2,223)<br><br>RO-NN-1 nt 3,255,511- nt 3,257,480 replaces DK1042 3,454,718- nt 3,456,695 (*estA* nt 258- *rghRA* nt 15)	This study	S19
JMB348	RO-NN-1 nt 3,255,511-nt 3,258,045 replaces DK1042 nt 3,454,718- nt 3,457,261 (*estA* nt 258- *rghRB* nt 144)	This study	S19
JMB349	RO-NN-1 nt 3,256,683- nt 3,258,201 replaces DK1042 nt 3,455,890- nt 3,457,417 (*yvaM* nt 405- *rghRB* nt 300)	This study	3, S19
JMB350	RO-NN-1 nt 3,256,050- nt 3,257,867 replaces DK1042 nt 3,455,257- nt 3,457,082 (*secG* nt 81- *rghRA* nt 402)	This study	S19
JMB351	RO-NN-1 nt 3,256,863 replaces DK1042 nt 3,456,070 (*yvaM* nt 585)<br><br>RO-NN-1 nt 3,257,348- nt 3,257,435 replaces DK1042 nt 3,456,555- nt 3,456,650 (126 bp upstream of *rghRA*- 31 bp upstream of *rghRA*)	This study	3, S19
JMB352	RO-NN-1 nt 3,256,740 replaces DK1042 nt 3,455,947 (*yvaM* nt 462)<br><br>RO-NN-1 nt 3,257,335- nt 3,257,480 replaces DK1042 nt 3,456,547- nt 3,456,695 (134 bp upstream of *rghRA*- *rghRA* nt 15)	This study	3, S19, S20
JMB353	*B. subtilis* P1A7 Δ*hisB*	This study	4, S22, S23, S25, S26, S27
JMB354	*B. subtilis* P1A11 Δ*hisB*	This study	4, S22, S23, S25, S26, S27

JMB355	*B. subtilis* P1B5 Δ*hisB*	This study	4, S22, S23, S25, S26, S27
JMB356	*B. subtilis* P1B12 Δ*hisB*	This study	4, S22, S23, S25, S26, S27
JMB357	*B. subtilis* P1E9 Δ*hisB*	This study	4, S22, S23, S25, S26, S27
JMB358	*B. subtilis* P1F11 Δ*hisB*	This study	4, S22, S23, S25, S26, S27
JMB359	*B. subtilis* P2B1 Δ*hisB*	This study	4, S22, S23, S25, S26, S27
JMB360	*B. subtilis* P2D3 Δ*hisB*	This study	4, S22, S23, S25, S26, S27
JMB361	*B. subtilis* P2D4 Δ*hisB*	This study	4, S22, S23, S25, S26, S27
JMB362	*B. subtilis* P2E11 Δ*hisB*	This study	4, S22, S23, S25, S26, S27
JMB363	*B. subtilis* P2G8 Δ*hisB*	This study	4, S22, S23, S25, S26, S27
JMB364	*B. subtilis* P2G10 Δ*hisB*	This study	4, S22, S23, S25, S26, S27
JMB365	*B. subtilis* P3B5 Δ*hisB*	This study	4, S22, S23, S25, S26, S27
JMB366	*B. subtilis* P3C6 Δ*hisB*	This study	4, S22, S23, S25, S26, S27
JMB367	*B. subtilis* P3D3 Δ*hisB*	This study	4, S22, S23, S25, S26, S27
JMB368	*B. subtilis* P3E3 Δ*hisB*	This study	4, S22, S23, S25,

			S26, S27
JMB369	*B. subtilis* P3F1 Δ*hisB*	This study	4, S22, S23, S25, S26, S27
JMB370	*B. subtilis* P3F10 Δ*metE*	This study	4, S22, S23, S25, S26, S27
JMB371	*B. subtilis* P3G3 Δ*metE*	This study	4, S22, S23, S25, S26, S27
JMB372	*B. subtilis* P3G12 Δ*metE*	This study	4, S22, S23, S25, S26, S27
JMB373	*B. subtilis* P3H8 Δ*metE*	This study	4, S22, S23, S25, S26, S27
JMB374	*B. subtilis* P4A9 Δ*metE*	This study	4, S22, S23, S25, S26, S27
JMB375	*B. subtilis* P4B5 Δ*metE*	This study	4, S22, S23, S25, S26, S27
JMB376	*B. subtilis* RO-NN-1 carrying pJM609	This study	S24
JMB377	JMB300 carrying pJM609	This study	S24
JMB378	*B. subtilis* P1A7 Δ*hisB* carrying pJM609	This study	S24
JMB379	*B. subtilis* P1A11 Δ*hisB* carrying pJM609	This study	S24
JMB380	*B. subtilis* P1B5 Δ*hisB* carrying pJM609	This study	S24
JMB381	*B. subtilis* P1B12 Δ*hisB* carrying pJM609	This study	S24
JMB382	*B. subtilis* P1E9 Δ*hisB* carrying pJM609	This study	S24
JMB383	*B. subtilis* P1F11 Δ*hisB* carrying pJM609	This study	S24
JMB384	*B. subtilis* P2B1 Δ*hisB* carrying pJM609	This study	S24
JMB385	*B. subtilis* P2D3 Δ*hisB* carrying pJM609	This study	S24
JMB386	*B. subtilis* P2D4 Δ*hisB* carrying pJM609	This study	S24
JMB387	*B. subtilis* P2E11 Δ*hisB* carrying pJM609	This study	S24

JMB388	*B. subtilis* P2G8 Δ*hisB* carrying pJM609	This study	S24
JMB389	*B. subtilis* P2G10 Δ*hisB* carrying pJM609	This study	S24
JMB390	*B. subtilis* P3B5 Δ*hisB* carrying pJM609	This study	S24
JMB391	*B. subtilis* P3C6 Δ*hisB* carrying pJM609	This study	S24
JMB392	*B. subtilis* P3D3 Δ*hisB* carrying pJM609	This study	S24
JMB393	*B. subtilis* P3E3 Δ*hisB* carrying pJM609	This study	S24
JMB394	*B. subtilis* P3F1 Δ*hisB* carrying pJM609	This study	S24
JMB395	*B. subtilis* P3F10 Δ*metE* carrying pJM609	This study	S24
JMB396	*B. subtilis* P3G3 Δ*metE* carrying pJM609	This study	S24
JMB397	*B. subtilis* P3G12 Δ*metE* carrying pJM609	This study	S24
JMB398	*B. subtilis* P3H8 Δ*metE* carrying pJM609	This study	S24
JMB399	*B. subtilis* P4A9 Δ*metE* carrying pJM609	This study	S24
JMB400	*B. subtilis* P4B5 Δ*metE* carrying pJM609	This study	S24
JMB401-403, JMB429	(RO-NN-1 x DK1042) S4 Δ*hisB*::*kan* Δ*metE*::*erm* pool x TU-B-10 → *hisB*$^{+}$ Δ*metE*::*erm*	This study	S28
JMB404-406, JMB430	(RO-NN-1 x DK1042) S4 Δ*hisB*::*kan* Δ*metE*::*erm* pool x TU-B-10 → *metE*$^{+}$ Δ*hisB*::*kan*	This study	S28
JMB413	*B. velezensis* S4 carrying pIDV76	This study	
JMB414	*B. velezensis* S4 Δ*hisB*::*kan*	This study	
JMB415	*B. velezensis* S4 Δ*metE*::*erm*	This study	
JMB416	*B. velezensis* S4 Δ*hisB*::*kan* Δ*metE*::*erm*	This study	5
JMB417-420, JMB427-428	GB03 x S4 Δ*metE*::*erm* Δ*hisB*::*kan* → S1 Δ*metE*::*erm*	This study	5
JMC3-10	DSM 1313 Δ*pyrF*::*neo* Δ*trpC*::*cat* x S14 → S1 *pyrF*$^{+}$ Δ*trpC*::*cat*	This study	5
JMN79, JMN80, JMN84, JMN100, JMN102, JMN103, JMN105, JMN106	F199 Δ*hisB*::*kan* Δ*leuB*::*cat* x B0695 → S1 Δ*hisB*::*kan* *leuB*$^{+}$	This study	5

JMS3-8	DSM 4166 x A1501 Δ*trpC*::*apr* Δ*hisB*::*kan* → S1 Δ*trpC*::*apr* Δ*hisB*::*kan*	This study	5

Supplementary Table 2. Plasmids used in this study.

Plasmid	Genotype	Reference
pDR244	*ori*$_{pACYC}$, *spcR*, *rep* p15A^{ts}, P$_{PA}$-*cre*	[26]
pJOE9658.1	*ori*$_{pUC18}$, *kanR*, *rep* pE194^{ts}, gRNA, P$_{tetLM}$-*tetLM-cas9*	[5]
pHT254	P$_{grac100}$-MCS-His, *cmR*, *ampR*	[28]
pIDV40	*ori*$_{ColE1}$, ,*cmR*, *ampR*, rep pE194^{ts}, P$_{dnaX}$-*mKate2*	Gift from Dr. Ilenne Del Valle
pUBXC	*ori*$_{pUC}$, *zeoR*, *xylR*, P$_{xylA}$-*comK*$_{Bsu}$	[29]
pIDV76	pUBXC, *catR*, *xylR*, P$_{xylA}$-*comK*$_{Bsu}$-*comS*	Gift from Dr. Ilenne Del Valle
pDGO145	*ori*$_{p15A}$, *ampR*, *rep* pNW33N, P$_{cbp}$-*tdk*	[30]
pAK405	Allele exchange plasmid for sphingomonads, *kanR*, *rpsL* (sm^S)	[31]
pJM598	pJOE9658-sgRNA(pBS32-*repN*)	This study
pJM599	pJOE9658-sgRNA(3610-*gerAA*)	This study
pJM600	pJOE9658-sgRNA(3610-*gerAB*)	This study
pJM601	pJOE9658-sgRNA(3610-*gerAC*)	This study
pJM602	pJOE9658-sgRNA(3610-*gerAC*)-*gerAC*(RO-NN-1)	This study
pJM603	pJOE9658-sgRNA(3610-*rnr*)	This study
pJM604	pJOE9658-sgRNA(3610-*yvaQ*)	This study
pJM605	pJOE9658-sgRNA(3610-*opuBC*)	This study
pJM606	pJOE9658-sgRNA(3610-*rghRA*)	This study
pJM607	pJOE9658-sgRNA(RO-NN-1-*trpC*)-*trpC*(RO-NN-1)	This study
pJM608	pJOE9658-sgRNA(3610-*trpC*)-*trpC*(3610)	This study
pJM609	pHT254-YmwC^{SP}-*xylA*	This study
pJM611	pDGO145, DSM 1313 *trpC*::*cat*	This study
pJM612	pDGO145, DSM 1313 *pyrF*::*neo*	This study
pJM613	pAK405, F199 *hisB*::*kan*	This study
pJM614	pAK405, F199 *leuB*::*cat*	This study

Supplementary Table 3. Oligonucleotides used in this study.

Oligo	Sequence	Used for
**hisB-FL**	CAATTGCCGGATATAATGTAAAAGCAC	Construction of DK1042 histidine auxotrophic strain
**hisB-RL**	ATATGATTGCCGGACCGAGTGAAATC	
**metE-FL**	CATGCCTGATCCTTTTAATATTCTTTCTTATTG	Construction of DK1042 methionine auxotrophic strain
**metE-RL**	GCTATGAAGAAGAATCATTTCAAAGAAAG	
**oDV001**	TATTAGCTAGAGCTGTGC	Deletion of *gerA* in strain RO-NN-1
**oDV002**	tgtatgctatacgaacggtaCAATGAGGTCACCTCTTATC	
**oDV003**	gataagaggtgacctcattgTACCGTTCGTATAGCATAC	
**oDV004**	cgtgaattaggcggctgctaTCTACCGTTCGTATAATGTATG	
**oDV005**	tacattatacgaacggtagaTAGCAGCCGCCTAATTCAC	
**oDV006**	GTCATCGGCGAAGCATCG	
**oDV007**	TACGATTGGAAAGACGAACTCGGA	pBS32(sgRNA-*repN*)
**oDV008**	AAACTCCGAGTTCGTCTTTCCAAT	
**oDV009**	TACGAGTCACCATCGAGCTGCTAA	3610(sgRNA-*gerAA*)
**oDV010**	AAACTTAGCAGCTCGATGGTGACT	
**oDV011**	TACGTAAAAAAGAGATGGAAACGA	3610(sgRNA-*gerAB*)
**oDV012**	AAACTCGTTTCCATCTCTTTTTTA	
**oDV013**	TACGTAAGGTGACGATCAGAACGA	3610(sgRNA-*gerAC*)
**oDV014**	AAACTCGTTCTGATCGTCACCTTA	
**oDV015**	AAGGCCAACGAGGCCTTGGAACAAACAGAGTTTAAGGA	RO-NN-1 *gerAA* repair template and RO-NN-1 *gerAA-gerAC* repair

**oDV016**	AAGGCCTTATTGGCCTTAAGTTTCAGTGGAGTCTGTT	template
**oDV017**	AAGGCCAACGAGGCCATGAGCCAAAAACAGACTCCAC	RO-NN-1 *gerAB* repair template
**oDV018**	AAGGCCTTATTGGCCTCATTTTGCTGTAATCCTCCTTTTG	
**oDV019**	AAGGCCAACGAGGCCATGAAAATCCGGATTTTATGTATGT	RO-NN-1 *gerAC* repair template and RO-NN-1 *gerAA-gerAC* repair template
**oDV020**	AAGGCCTTATTGGCCCTATTTGTTTGCGCCTTTCGT	
**oDV021**	TACGTGTAGCAAACGAAACAGTTG	3610(sgRNA-*rnr*)
**oDV022**	AAACCAACTGTTTCGTTTGCTACA	
**oDV023**	TACGAGCCATTTCGTTCATCGTCT	3610(sgRNA-*yvaQ*)
**oDV024**	AAACAGACGATGAACGAAATGGCT	
**oDV025**	TACGGCAGCAGGCTTTAATGAACG	3610(sgRNA-*opuBC*)
**oDV026**	AAACCGTTCATTAAAGCCTGCTGC	
**oDV027**	TACGCTAAAATATGAGTAACAGTG	3610(sgRNA-*rghRA*)
**oDV028**	AAACCACTGTTACTCATATTTTAG	
**oDV029**	TGAATGGCGTCGTGATCTGA	RO-NN-1 repair templates for construction of strains JMB335-338 and JMB344-352
**oDV030**	ACGGCTCTTTTCTCGTCTTTG	
**oDV031**	ACATTTCCAGTCTACCTATTCCC	RO-NN-1 repair templates for construction of strains JMB340-343
**oDV032**	CTCAATCAAACGGCTGAGC	
**oDV033**	TACGTACGGGAATCAAGAAGAATCGGAG	3610/RO-NN-1(sgRNA-*trpC*)
**oDV034**	AAACAAACCTCCGATTCTTCTTGATTCC	

**oAM001**	TTTTGTAAATTGCGTTAAGATCGCCATT	Construction of S4 histidine auxotrophic strain
**oAM002**	ATACGGCGGTGCCCAAAGAAG	
**oAM003**	GGGACGAATGGATTATTCAAGAGG	Construction of S4 methionine auxotrophic strain
**oAM004**	GAAGACGTCCGTGCCGCAAA	

Sequences shown in red are BsaI-compatible overhangs used for cloning 20-nucleotide sgRNA spacer sequences targeting regions of interest into pJOE9658.1. Sequences shown in blue are SfiI recognition sites used for cloning repair templates into pJOE9658.1.

References

1. Zhang, Z. *et al.* Mixed linear model approach adapted for genome-wide association studies. *Nat Genet* **42**, 355–360 (2010).
2. Gao, Y. *et al.* Bacterial spore germination receptors are nutrient-gated ion channels. *Science* **380**, 387–391 (2023).
3. Artzi, L. *et al.* Dormant spores sense amino acids through the B subunits of their germination receptors. *Nat Commun* **12**, 6842 (2021).
4. Wilson, M. J., Carlson, P. E., Janes, B. K. & Hanna, P. C. Membrane topology of the *Bacillus anthracis* GerH germinant receptor proteins. *J Bacteriol* **194**, 1369–1377 (2012).
5. Toymentseva, A. A. & Altenbuchner, J. New CRISPR-Cas9 vectors for genetic modifications of *Bacillus* species. *FEMS Microbiol. Lett.* **366**, (2019).
6. Earl, A. M. *et al.* Whole-genome sequences of *Bacillus subtilis* and close relatives. *J. Bacteriol.* **194**, 2378–2379 (2012).
7. Cohan, F. M., Roberts, M. S. & King, E. C. The potential for genetic exchange by transformation within a natural population of *Bacillus subtilis*. *Evolution* **45**, 1393–1421 (1991).
8. Zeigler, D. R. The genome sequence of *Bacillus subtilis* subsp. *spizizenii* W23: insights into speciation within the *B. subtilis* complex and into the history of *B. subtilis* genetics. *Microbiology* **157**, 2033–2041 (2011).
9. Srivatsan, A. *et al.* High-precision, whole-genome sequencing of laboratory strains facilitates genetic studies. *PLoS Genet.* **4**, e1000139 (2008).
10. Konkol, M. A., Blair, K. M. & Kearns, D. B. Plasmid-encoded ComI inhibits competence in the ancestral 3610 strain of *Bacillus subtilis*. *J Bacteriol* **195**, 4085–4093 (2013).
11. Roberts, M. S. & Cohan, F. M. Recombination and migration rates in natural populations of *Bacillus subtilis* and *Bacillus mojavensis*. *Evolution* **49**, 1081–1094 (1995).
12. Dunlap, C. A., Bowman, M. J. & Zeigler, D. R. Promotion of *Bacillus subtilis* subsp. *inaquosorum*, *Bacillus subtilis* subsp. spizizenii and *Bacillus subtilis* subsp. *stercoris* to species status. *Antonie Van Leeuwenhoek* **113**, 1–12 (2020).
13. Wockenfuss, A. *et al.* A strain shows antimicrobial activity against soilborne and foliar fungi and oomycetes. *Front Fungal Biol* **5**, 1332755 (2024).
14. Chen, X. H. *et al.* Comparative analysis of the complete genome sequence of the plant growth-promoting bacterium *Bacillus amyloliquefaciens* FZB42. *Nat Biotechnol* **25**, 1007–1014 (2007).
15. Mullins, A. J. *et al.* Reclassification of the biocontrol agents BY-2 and Tu-100 as and insights into the genomic and specialized metabolite diversity of the species. *Microbiology (Reading)* **166**, 1121–1128 (2020).
16. Choi, S.-K., Jeong, H., Kloepper, J. W. & Ryu, C.-M. Genome Sequence of *Bacillus*

amyloliquefaciens GB03, an Active Ingredient of the First Commercial Biological Control Product. *Genome Announc* **2**, (2014).

17. Sibponkrung, S. *et al.* Genome Sequence of S141, a New Strain of Plant Growth-Promoting Rhizobacterium Isolated from Soybean Rhizosphere. *Genome Announc* **5**, (2017).
18. Carper, D. L. *et al.* Cultivating the Bacterial Microbiota of Populus Roots. *mSystems* **6**, e0130620 (2021).
19. Feinberg, L. *et al.* Complete genome sequence of the cellulolytic thermophile *Clostridium thermocellum* DSM1313. *J Bacteriol* **193**, 2906–2907 (2011).
20. Tachaapaikoon, C. *et al.* Isolation and characterization of a new cellulosome-producing *Clostridium thermocellum* strain. *Biodegradation* **23**, 57–68 (2012).
21. Yan, Y. *et al.* Nitrogen fixation island and rhizosphere competence traits in the genome of root-associated *Pseudomonas stutzeri* A1501. *Proc Natl Acad Sci U S A* **105**, 7564–7569 (2008).
22. Krotzky, A. & Werner, D. Nitrogen fixation in *Pseudomonas stutzeri*. *Arch. Microbiol.* **147**, 48–57 (1987).
23. Yu, H. *et al.* Complete genome sequence of the nitrogen-fixing and rhizosphere-associated bacterium *Pseudomonas stutzeri* strain DSM4166. *J Bacteriol* **193**, 3422–3423 (2011).
24. Romine, M. F. *et al.* Complete sequence of a 184-kilobase catabolic plasmid from *Sphingomonas aromaticivorans* F199. *J. Bacteriol.* **181**, 1585–1602 (1999).
25. Fredrickson, J. K. *et al.* Aromatic-degrading *Sphingomonas* isolates from the deep subsurface. *Appl. Environ. Microbiol.* **61**, 1917–1922 (1995).
26. Koo, B.-M. *et al.* Construction and Analysis of Two Genome-Scale Deletion Libraries for *Bacillus subtilis*. *Cell Syst* **4**, 291–305.e7 (2017).
27. Vasileva, D. P. *et al.* Protoplast fusion in *Bacillus* species produces frequent, unbiased, genome-wide homologous recombination. *Nucleic Acids Res.* **50**, 6211–6223 (2022).
28. Phan, T. T. P., Tran, L. T., Schumann, W. & Nguyen, H. D. Development of Pgrac100-based expression vectors allowing high protein production levels in *Bacillus subtilis* and relatively low basal expression in *Escherichia coli*. *Microb Cell Fact* **14**, 72 (2015).
29. Chen, X.-T. *et al.* Artificial induction of genetic competence in *Bacillus amyloliquefaciens* isolates. *Biotechnol Lett* **38**, 2109–2117 (2016).
30. Hon, S. *et al.* The ethanol pathway from *Thermoanaerobacterium saccharolyticum* improves ethanol production in *Clostridium thermocellum*. *Metab Eng* **42**, 175–184 (2017).
31. Kaczmarczyk, A., Vorholt, J. A. & Francez-Charlot, A. Markerless gene deletion system for sphingomonads. *Appl. Environ. Microbiol.* **78**, 3774–3777 (2012).
