# Supplementary material for: Genome shuffling enables quantitative trait locus mapping in Bacillus subtilis
Source: Nat Commun. 2026 Aug 5;17:7852. doi: 10.1038/s41467-026-72929-0 (PMC13443641; doi:10.1038/s41467-026-72929-0)
Supplement: Supplementary file 3 [file 41467_2026_72929_MOESM3_ESM.pdf]

Description of Additional Supplementary Files:

Supplementary Data 1: Spore germination phenotypes of the *B. subtilis* QTL mapping population.

Supplementary Data 2: Swarming motility phenotypes of the *B. subtilis* QTL mapping population.

Supplementary Data 3: Global proteomic analysis of swarm cells from *B. subtilis* RO-NN-1, DK1042 and JMB352.

Supplementary Data 4: Dendrogram showing hierarchical clustering of the 350 *B. subtilis* shuffled strains based on genetic distance.
