# Supplementary material for: Genome shuffling enables quantitative trait locus mapping in Bacillus subtilis
Source: Nat Commun. 2026 Aug 5;17:7852. doi: 10.1038/s41467-026-72929-0 (PMC13443641; doi:10.1038/s41467-026-72929-0)
Supplement: Supplementary file 4 [file 41467_2026_72929_MOESM7_ESM.pdf]

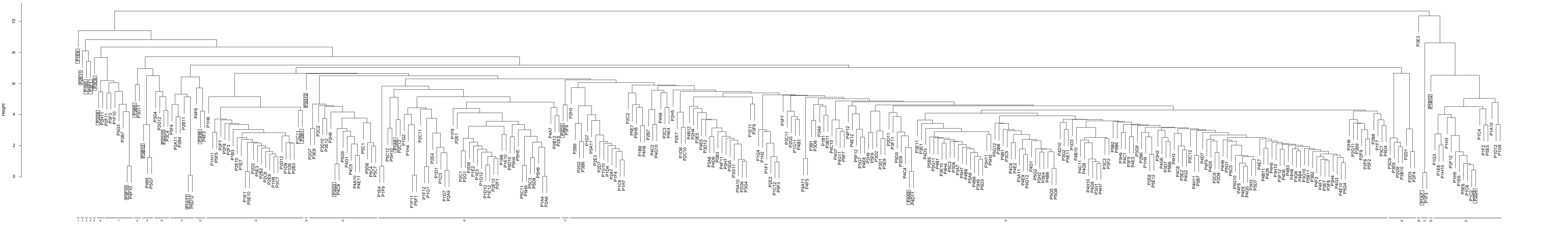
Height
10
8
6
4
2
0
