# Supplementary material for: Genome shuffling enables quantitative trait locus mapping in Bacillus subtilis
Source: Nat Commun. 2026 Aug 5;17:7852. doi: 10.1038/s41467-026-72929-0 (PMC13443641; doi:10.1038/s41467-026-72929-0)
Supplement: Supplementary file 5 [file 41467_2026_72929_MOESM8_ESM.pdf]

nature portfolio

Corresponding author(s): Daniel Jacobson
Josh Michener

Last updated by author(s): 2026-4-22

Reporting Summary

Nature Portfolio wishes to improve the reproducibility of the work that we publish. This form provides structure for consistency and transparency in reporting. For further information on Nature Portfolio policies, see our Editorial Policies and the Editorial Policy Checklist.

Statistics

For all statistical analyses, confirm that the following items are present in the figure legend, table legend, main text, or Methods section.

n/a	Confirmed	
[ ]	[x]	The exact sample size (*n*) for each experimental group/condition, given as a discrete number and unit of measurement
[ ]	[x]	A statement on whether measurements were taken from distinct samples or whether the same sample was measured repeatedly
[ ]	[x]	The statistical test(s) used AND whether they are one- or two-sided<br>*Only common tests should be described solely by name; describe more complex techniques in the Methods section.*
[x]	[ ]	A description of all covariates tested
[ ]	[x]	A description of any assumptions or corrections, such as tests of normality and adjustment for multiple comparisons
[ ]	[x]	A full description of the statistical parameters including central tendency (e.g. means) or other basic estimates (e.g. regression coefficient) AND variation (e.g. standard deviation) or associated estimates of uncertainty (e.g. confidence intervals)
[ ]	[x]	For null hypothesis testing, the test statistic (e.g. F, t, r) with confidence intervals, effect sizes, degrees of freedom and P value noted<br>*Give P values as exact values whenever suitable.*
[x]	[ ]	For Bayesian analysis, information on the choice of priors and Markov chain Monte Carlo settings
[x]	[ ]	For hierarchical and complex designs, identification of the appropriate level for tests and full reporting of outcomes
[x]	[ ]	Estimates of effect sizes (e.g. Cohen's *d*, Pearson's *r*), indicating how they were calculated

Our web collection on statistics for biologists contains articles on many of the points above.

Software and code

Policy information about availability of computer code

Data collection	Gen 5 v3.03 (BioTek, Winooski, VT, USA) was used for spectrophotometric assays. EOS Utility 2 (Canon, Tokyo, Japan) was used for microscope image acquisition.
Data analysis	Geneious Prime v2019.0, v 2023.0 and v2024.0, Trimmomatic v0.39, BWA v0.7.17 , SAMtools v1.3.1, GATK v4.1.8.1 were used for analysis of genomic data and genetic mapping. Source code of the Python script used to visualize genomes of B. subtilis recombinant strains is available on Github at https://github.com/LaneMatthewJ/heritability_illustrator/. The source code used for QTL mapping is available on Github repository at https://github.com/harichhetri/Bsubtilis_qtl_mapping. Skyline v24.1.0 was used to process LC-MS/MS data.

For manuscripts utilizing custom algorithms or software that are central to the research but not yet described in published literature, software must be made available to editors and reviewers. We strongly encourage code deposition in a community repository (e.g. GitHub). See the Nature Portfolio guidelines for submitting code & software for further information.

Data

Policy information about availability of data

All manuscripts must include a data availability statement. This statement should provide the following information, where applicable:

- Accession codes, unique identifiers, or web links for publicly available datasets
- A description of any restrictions on data availability
- For clinical datasets or third party data, please ensure that the statement adheres to our policy

Data availability:

The sequencing data generated in this study for the B. subtilis mapping population have been deposited in the NCBI BioProject database under accession code PRJNA1330897 [https://www.ncbi.nlm.nih.gov/bioproject/?term=PRJNA1330897]. The sequencing data generated in this study for the B. velezensis, C. thermocellum, N. aromaticivorans, and S. stutzeri shuffled strains, as well as recombinant B. subtilis strains generated via triparental shuffling, have been deposited in the NCBI Sequence Read Archive database under accession code PRJNA1451957 [https://www.ncbi.nlm.nih.gov/bioproject/1451957]. The Proteomics data generated in this study have been deposited in the ProteomeXchange Consortium via the MASSIVE repository (MSV000101244) under accession code PXD076164 [https://massive.ucsd.edu/ProteoSAFe/dataset.jsp?task=25bcd3fd3793438c8fdef17d84d0efbb]. Other data are included in the supplementary materials. Source data are provided with this paper.

Code availability:
The source code used to generate a recombination map of the B. subtilis QTL population is available at https://github.com/LaneMatthewJ/heritability_illustrator/ and has been deposited in the Zenodo repository at https://doi.org/10.5281/zenodo.19687706. The source code used for genetic mapping is available at https://github.com/harichhetri/Bsubtilis_qtl_mapping and has been deposited in the Zenodo repository at https://doi.org/10.5281/zenodo.19654579.

Research involving human participants, their data, or biological material

Policy information about studies with human participants or human data. See also policy information about sex, gender (identity/presentation), and sexual orientation and race, ethnicity and racism.

Reporting on sex and gender	NA
Reporting on race, ethnicity, or other socially relevant groupings	NA
Population characteristics	NA
Recruitment	NA
Ethics oversight	NA

Note that full information on the approval of the study protocol must also be provided in the manuscript.

Field-specific reporting

Please select the one below that is the best fit for your research. If you are not sure, read the appropriate sections before making your selection.

☒ Life sciences ☐ Behavioural & social sciences ☐ Ecological, evolutionary & environmental sciences

For a reference copy of the document with all sections, see nature.com/documents/nr-reporting-summary-flat.pdf

Life sciences study design

All studies must disclose on these points even when the disclosure is negative.

Sample size	No statistical method was used to predetermine sample size. For most experiments reported in this study, we performed three independent biological replicates, which is a generally accepted number of independent replicates in microbiology. For some assays, we performed more than three biological replicates as indicated in the figure legends.
Data exclusions	No data were excluded from analyses.
Replication	Independent experiments were carried out to verify the reproducibility of the experimental findings. The number of independent experiments is indicated for each figure. Details on statistics are also included in the figure legends and the methods section.
Randomization	No randomization was performed. All assays were carried out using genetically homogeneous cultures under controlled environments.
Blinding	Data collection and analysis were not carried out blindly. Assays involved automated data collection (except for colony counts) and analysis was software-supported (except for non-quantifiable microscopy and colony morphology data).

Reporting for specific materials, systems and methods

We require information from authors about some types of materials, experimental systems and methods used in many studies. Here, indicate whether each material, system or method listed is relevant to your study. If you are not sure if a list item applies to your research, read the appropriate section before selecting a response.

Materials & experimental systems

n/a	Involved in the study
☒	☐ Antibodies
☒	☐ Eukaryotic cell lines
☒	☐ Palaeontology and archaeology
☒	☐ Animals and other organisms
☒	☐ Clinical data
☒	☐ Dual use research of concern
☒	☐ Plants

Methods

n/a	Involved in the study
☒	☐ ChIP-seq
☒	☐ Flow cytometry
☒	☐ MRI-based neuroimaging

Plants

Seed stocks	NA
Novel plant genotypes	NA
Authentication	NA
